# The Caffeinated Brain Part 3: The Effect of Caffeine on Electroencephalography (EEG) During Wakefulness—A Systematic and Mechanistic Review

**DOI:** 10.3390/nu18172898

**Published:** 2026-09-03

**Authors:** James Chmiel, Aleksandra Kładna

**Affiliations:** 1 Faculty of Physical Culture and Health, Institute of Physical Culture Sciences, University of Szczecin, Al. Piastów 40B Blok 6, 71-065 Szczecin, Poland; 2 Department of the History of Medicine and Medical Ethics, Pomeranian Medical University, ul. Rybacka 1, 70-204 Szczecin, Poland; aleksandra.kladna@pum.edu.pl

**Keywords:** caffeine, adenosine receptor antagonism, electroencephalography, EEG oscillations, wakefulness, cortical arousal, vigilance, alpha rhythm, theta activity, sleep pressure

## Abstract

**Introduction:** Caffeine is a widely consumed psychoactive compound whose central pharmacological effects are mediated primarily through antagonism of adenosine receptors. Wakeful EEG provides a temporally sensitive method for examining caffeine-related changes in oscillatory activity and vigilance-related brain states. This systematic review synthesized evidence concerning the effects of acute and habitual caffeine exposure on non-ERP EEG measures during wakefulness. **Materials and Methods:** The review followed PRISMA 2020 guidelines. PubMed/MEDLINE, Scopus, Web of Science, Embase, PsycINFO, and the Cochrane Library were searched from database inception to 30 May 2026. Eligible studies included human participants exposed to caffeine, coffee, or caffeine-containing interventions and reported EEG outcomes during wakeful resting-state or task-related conditions. Risk of bias was assessed using RoB 2 for randomized trials and ROBINS-I for non-randomized studies. Findings were synthesized narratively because of substantial methodological heterogeneity. **Results:** Forty-eight studies were included. Resting-state spectral EEG was the dominant paradigm, although studies also examined task-related activity, prolonged wakefulness, sleep deprivation, withdrawal, driving-related drowsiness, coherence, nonlinear complexity, EEG microstates, and multimodal EEG–fMRI measures. Reductions in alpha power or amplitude were among the more frequently reported findings following acute caffeine administration, while attenuation of theta, delta, or other slow-frequency activity was particularly evident under conditions of sleep pressure, prolonged wakefulness, withdrawal, fatigue, or reduced vigilance. However, neither effect was universal. Beta findings were heterogeneous, and changes in alpha frequency, total EEG power, coherence, asymmetry, complexity, and microstates were less consistently examined. The direction and magnitude of EEG responses varied according to caffeine dose, habitual intake and withdrawal status, baseline vigilance, eye condition, task demands, participant characteristics, scalp region, frequency-band definition, and EEG methodology. Evidence concerning coherence, asymmetry, nonlinear complexity, microstates, and multimodal EEG–fMRI outcomes was preliminary. **Conclusions:** The available evidence does not establish a single reproducible EEG signature or validated electrophysiological biomarker of caffeine response. Reductions in alpha power and attenuation of slow-frequency activity during states of elevated sleep pressure or caffeine withdrawal were among the more frequently reported findings, but substantial methodological and state-dependent heterogeneity limits their specificity and generalizability. EEG changes should also not be interpreted as direct indicators of improved cognitive performance, because electrophysiological and behavioral effects were not consistently concordant across studies.

## 1. Introduction

Caffeine is a widely consumed psychoactive methylxanthine whose central effects are mediated primarily through competitive antagonism of adenosine A1 and A2A receptors, rather than through nonspecific stimulation alone [1,2,3]. By reducing adenosinergic inhibition, caffeine modulates neural systems involved in arousal, vigilance, attention, motor readiness, and fatigue resistance, which explains its robust acute effects on reaction time, alertness, and sustained cognitive performance [4,5,6]. Nevertheless, the interpretation of caffeine-related cognitive enhancement remains complex, because some apparent benefits may reflect reversal of withdrawal-related decrements in habitual users, while interindividual variability is additionally shaped by tolerance, baseline sleep pressure, habitual intake, and genetic factors influencing caffeine metabolism and adenosine receptor sensitivity, particularly CYP1A2 and ADORA2A-related mechanisms [7,8].

Beyond its pharmacological significance, caffeine exposure is highly relevant in everyday and operational settings because consumption is widespread and occurs through heterogeneous sources and dosing patterns. Coffee and tea remain major dietary sources, while caffeinated soft drinks and energy drinks constitute additional sources, particularly in younger populations; caffeine is also consumed in tablets, dietary and pre-workout supplements, and selected medications. Exposure may therefore range from small repeated doses distributed across the day to single moderate or high doses taken deliberately to increase alertness or performance. Caffeine is commonly used to counteract fatigue and reduced vigilance during sleep restriction, night or shift work, prolonged driving and other monotonous activities, as well as during demanding cognitive and athletic tasks. This variability in source, dose, timing, and purpose of use is directly relevant to EEG research because it modifies both the pharmacological exposure and the participant’s pre-existing level of arousal and sleep pressure.

The same pharmacological profile also explains caffeine’s double-edged physiological effects. Although caffeine can counteract sleepiness and maintain performance under fatigue, its antagonism of sleep-promoting adenosine signaling may impair subsequent sleep architecture, reducing total sleep time and sleep efficiency while increasing sleep latency and wakefulness after sleep onset, especially when consumed later in the day or at higher doses [9,10,11]. In exercise science, caffeine is among the most consistently supported ergogenic aids, with evidence indicating improvements across endurance, strength, power, and sport-specific tasks, particularly when consumed in moderate doses of approximately 3–6 mg/kg body mass, although lower doses may also be effective in selected contexts [12,13,14]. However, caffeine should not be treated as a universally beneficial stimulant: higher intake may increase anxiety-related symptoms in susceptible individuals, and its net effect depends on dose, timing, biological sensitivity, habitual use, and the cognitive or physiological domain being assessed [15].

Electroencephalography (EEG) is a non-invasive neurophysiological method that records voltage fluctuations from the scalp, reflecting the summed postsynaptic activity of large populations of cortical pyramidal neurons arranged in synchronously active networks [16,17]. Because EEG captures neural activity with millisecond temporal resolution, it is particularly suitable for studying rapid changes in brain state, vigilance, sensory processing, attention, and cognitive control during wakefulness [16,17,18,19]. In the awake brain, spontaneous EEG is commonly organized into oscillatory frequency bands, including delta, theta, alpha, beta, and gamma activity, but these rhythms should not be interpreted as fixed “states”; rather, their meaning depends on topography, task context, eye condition, arousal level, and individual neurophysiological traits [17,20,21,22]. During relaxed wakefulness with eyes closed, posterior alpha activity is usually prominent, whereas eye opening, visual engagement, and active attention are typically associated with alpha attenuation and changes in faster rhythms [18,19,21]. Wakefulness is also dynamic: prolonged time awake and sleep pressure may alter theta–alpha activity, vigilance stability, and the probability of attentional lapses, indicating that waking EEG contains graded markers of alertness rather than a simple binary distinction between wake and sleep [23,24]. For the purposes of the present review, wakeful EEG refers to EEG recorded while participants are behaviorally awake, but it is not treated as a single physiological state. It includes relaxed eyes-closed rest, eyes-open rest, active cognitive performance, sustained-attention or vigilance paradigms, driving or other monotonous tasks, rested wakefulness, sleep restriction or total sleep deprivation, caffeine abstinence or withdrawal, and wakeful recordings obtained in clinical or psychiatric populations. These conditions differ substantially in baseline arousal, sensory input, cognitive demand, and homeostatic sleep pressure and therefore cannot be assumed to yield interchangeable EEG responses. Accordingly, caffeine-related EEG findings are interpreted in this review according to the participant’s baseline physiological state, eye condition, and task context rather than being treated as observations from a single undifferentiated category of “wakefulness.”

Beyond conventional spectral analysis, wakeful EEG can also be examined through the framework of EEG microstates, defined as brief, quasi-stable configurations of the scalp electric field that persist for tens to hundreds of milliseconds before rapidly transitioning to another configuration [25,26]. Microstate analysis is especially relevant for resting-state wakefulness because it treats spontaneous EEG not as random background noise but as a structured temporal sequence of large-scale brain network states [25,26,27]. Classical microstate parameters, including duration, occurrence, coverage, global explained variance, and transition probabilities, provide information about the stability and temporal organization of wakeful brain activity [25,26,28]. Importantly, microstate features differ across eyes-open and eyes-closed conditions, cognitive demands, developmental stages, and levels of consciousness, suggesting that they may capture rapid electrophysiological correlates of perception, attention, self-referential processing, and vigilance [27,28,29,30]. Thus, wakeful EEG should be understood as a temporally rich signal in which oscillatory power, connectivity, arousal-dependent changes, and microstate dynamics jointly describe the brain’s ongoing balance between internal mentation, sensory readiness, and cognitive engagement.

Mechanistically, antagonism of adenosine A1 and A2A receptors provides a plausible link between caffeine exposure and the EEG changes reviewed below. During sustained wakefulness, increasing adenosinergic signaling contributes to neuronal inhibition and the accumulation of homeostatic sleep pressure; caffeine attenuates this influence and thereby favors arousal-promoting neural activity. At the scalp level, however, this mechanism would not be expected to produce a single receptor-specific EEG signature. Instead, altered adenosinergic tone may manifest as state-dependent attenuation of synchronized low-frequency activity, particularly theta or delta activity when sleep pressure or drowsiness is elevated, suppression or reorganization of alpha activity during resting wakefulness, and, in some contexts, a relative shift toward faster or less synchronized activity. These electrophysiological effects should therefore be interpreted as indirect network-level consequences of altered adenosinergic inhibition, cortical synchronization, and vigilance regulation rather than as direct measures of A1 or A2A receptor occupancy.

A further essential interpretative distinction concerns the type of caffeine exposure. Acute caffeine administration after controlled abstinence, habitual caffeine consumption, repeated or chronic administration, caffeine abstinence and withdrawal, and re-administration following withdrawal represent physiologically and behaviorally non-equivalent conditions. The intervention matrix is also relevant: administration of pure caffeine permits relatively direct attribution of effects to caffeine, whereas coffee and multicomponent caffeine-containing beverages introduce additional bioactive constituents and contextual factors. This distinction is particularly important in habitual consumers because an apparent improvement in vigilance or normalization of EEG activity following caffeine administration may represent reversal of withdrawal-related impairment rather than enhancement above a genuinely caffeine-free, non-withdrawn baseline. Consequently, habitual intake, duration of preceding abstinence, withdrawal status, re-administration, and caffeine formulation are treated in the present review as central interpretative axes rather than merely as secondary moderators.

The purpose of this systematic review is therefore to synthesize and critically evaluate studies examining the acute and, where available, habitual effects of caffeine on EEG during wakefulness. Specifically, the review aims to determine how caffeine influences resting-state and task-related non-ERP EEG measures, including spectral power and frequency, tonic and prestimulus oscillatory activity, time-frequency event-related oscillatory measures, functional connectivity, network organization, vigilance-related indices, nonlinear EEG measures, and microstate parameters. Event-related oscillations, event-related desynchronization/synchronization (ERD/ERS), and event-related spectral perturbations (ERSPs) were considered eligible when quantified as frequency- or time-frequency-domain EEG measures. In contrast, conventional event-related potentials (ERPs), defined here as stimulus- or response-locked time-domain voltage components obtained by trial averaging and quantified primarily by component amplitude or latency, were outside the scope of the review. A further objective is to identify methodological factors that may explain inconsistencies across studies, including caffeine dose, administration route, abstinence duration, habitual consumption, participant characteristics, recording condition, EEG preprocessing, frequency-band definitions, and outcome selection. By integrating evidence across experimental paradigms, this review seeks to clarify whether caffeine produces a reproducible electrophysiological signature of increased wakeful arousal and cognitive readiness, or whether its EEG effects are better understood as context-dependent responses shaped by baseline neurophysiology, tolerance, and withdrawal reversal. Such synthesis may help establish EEG markers that are sensitive to caffeine-induced modulation of wakefulness and guide future experimental work using more standardized protocols and advanced EEG metrics.

## 2. Materials and Methods

### 2.1. Study Design and Reporting Framework

This systematic review was designed to synthesize evidence on the effects of caffeine on electroencephalographic activity during wakefulness. The review was conducted in accordance with the Preferred Reporting Items for Systematic Reviews and Meta-Analyses (PRISMA) 2020 guidelines. The methodological approach was developed before screening and included predefined eligibility criteria, search strategy, data extraction categories, and risk-of-bias procedures. The review focused on human studies in which caffeine administration or habitual caffeine exposure was examined in relation to EEG outcomes recorded during wakeful resting-state or task-related conditions.

### 2.2. Eligibility Criteria

Studies were considered eligible if they: (1) included human participants of any age or sex; (2) examined caffeine, coffee, or another caffeine-containing intervention in which caffeine was the primary active compound or its effects could be reasonably isolated; (3) recorded EEG during wakefulness; and (4) reported at least one eligible non-ERP electrophysiological outcome. Eligible EEG outcomes included resting-state spectral power or frequency, quantitative EEG, tonic or prestimulus task-related oscillatory activity, coherence or functional connectivity, nonlinear EEG measures, steady-state or frequency-domain responses, and EEG microstate parameters.

Conventional time-domain ERPs were excluded from the review. For the purposes of eligibility, conventional ERPs were defined as transient stimulus- or response-locked voltage deflections quantified primarily by component amplitude or latency (e.g., P1, N1, P2, N2, P3/P300, or related components). Studies that collected ERP data were not automatically excluded if they additionally reported separable eligible non-ERP EEG outcomes; in such cases, only the non-ERP EEG findings were extracted and synthesized. Event-related oscillatory measures were distinguished from conventional ERPs on the basis of signal derivation. Frequency- or time-frequency-domain measures, including ERD/ERS and ERSPs, were eligible, whereas averaged time-domain ERP component amplitudes and latencies were not. Accordingly, an article reporting ERPs was excluded only when it lacked an additional eligible continuous, spectral, oscillatory, connectivity, nonlinear, or microstate EEG outcome recorded during wakefulness.

This restriction was adopted because the review was designed to characterize caffeine-related modulation of ongoing wakeful brain-state and oscillatory EEG dynamics rather than transient stimulus-locked sensory or cognitive processing. Conventional ERPs differ from ongoing EEG measures in their signal derivation, temporal reference, outcome definitions, and neurophysiological interpretation, and their inclusion would have substantially broadened the scope of the synthesis.

Studies were excluded if they were conducted exclusively in animals or in vitro models, if EEG was recorded only during sleep, anesthesia, coma, or seizure monitoring without a wakeful experimental condition, or if the caffeine effect could not be separated from other active compounds. Studies were also excluded if they did not report original empirical data, such as narrative reviews, editorials, conference abstracts without sufficient data, protocols, or commentaries. When multiple publications appeared to report overlapping samples, the most complete or methodologically relevant report was retained.

### 2.3. Information Sources and Search Strategy

A systematic literature search was conducted in PubMed/MEDLINE, Scopus, Web of Science, Embase, PsycINFO, and the Cochrane Library from database inception to 30 May 2026. The search strategy combined controlled vocabulary, where available, and free-text terms related to caffeine, electroencephalography, and wakefulness. Search syntax was adapted to the indexing system and search fields of each database. The complete search strategy for every database, including the database platform, controlled-vocabulary terms, free-text terms, field tags, Boolean operators, filters, exact date of the search, and number of records retrieved, is provided in Supplementary Table S1. Reference lists of relevant reviews and included articles were also screened manually for potentially eligible studies.

No restrictions were imposed on publication year. Eligible reports were restricted to full-text, peer-reviewed journal articles published in English. The full-article requirement was adopted because assessment of caffeine exposure, EEG acquisition and preprocessing, outcome definitions, and risk-of-bias domains required methodological detail that was generally unavailable in conference abstracts. The English-language restriction reflected practical limitations in reliable full-text assessment and data extraction; its potential implications for language bias are acknowledged below. All retrieved records were imported into reference management software, and duplicates were removed before screening. The number of records retrieved from each individual information source was recorded before deduplication and is reported in Supplementary Table S1.

### 2.4. Study Selection

Study selection was performed in two stages. Two reviewers (J.C. and A.K.) independently screened the titles and abstracts of all records remaining after deduplication against the predefined eligibility criteria. Records considered potentially eligible by either reviewer were retrieved for full-text assessment. The same two reviewers independently assessed the full texts for eligibility. Decisions were therefore duplicated at both stages. Disagreements were resolved through discussion and consensus; if consensus could not initially be reached, the eligibility criteria were re-examined jointly before a final decision was made. A specific reason for exclusion was recorded for every article excluded during full-text assessment. The complete list of full-text exclusions and reasons is provided in Supplementary Table S2.

### 2.5. Data Extraction

Data were extracted using a standardized extraction form. Data extraction was performed independently by two reviewers (J.C. and A.K.) using a standardized extraction form. Extracted data were compared between reviewers, and discrepancies were resolved through discussion and consensus. The following information was collected from each included study: author, year, country, study design, sample size, participant characteristics, caffeine dose, form of administration, timing of EEG recording after caffeine intake, abstinence or withdrawal requirements, habitual caffeine consumption, control or placebo condition, EEG recording parameters, electrode montage, eyes-open or eyes-closed condition, task paradigm, preprocessing procedures, and EEG outcomes. Extracted EEG outcomes included spectral power in conventional frequency bands, dominant or peak frequency measures, tonic and prestimulus task-related oscillatory activity, coherence or functional connectivity indices, source-localized EEG measures, nonlinear or complexity measures, graph-theoretical metrics, steady-state frequency-domain responses, and EEG microstate parameters. Conventional time-domain ERP amplitude and latency outcomes were not extracted.

Where available, behavioral and physiological outcomes were also extracted, including vigilance, attention, reaction time, working memory, fatigue, sleepiness, heart rate, blood pressure, and subjective arousal. Particular attention was paid to variables that could moderate caffeine-related EEG effects, including dose, time since ingestion, age, sex, baseline arousal, sleep deprivation, habitual caffeine intake, caffeine abstinence duration, and genetic or metabolic factors.

### 2.6. EEG Outcome Domains and Outcome-Selection Rules

Because the eligible literature used multiple non-equivalent electrophysiological measures, a single scalar primary EEG outcome was not designated. Instead, the synthesis was organized into predefined EEG outcome domains: (1) spectral power or amplitude, including delta, theta, alpha, beta, gamma, and total or broadband EEG activity; (2) spectral-frequency measures, including mean, peak, or dominant frequency; (3) tonic, prestimulus, and time-frequency task-related oscillatory measures, including ERD/ERS and ERSPs; (4) coherence, functional connectivity, and network-level measures; (5) nonlinear or complexity measures; and (6) EEG microstate parameters. Conventional stimulus- or response-locked time-domain ERP amplitudes and latencies were not eligible outcomes.

Frequency bands were classified primarily according to the definitions used in each original study because band boundaries differed substantially across the literature. For descriptive comparison, conventional labels were interpreted approximately as delta, 0.5–4 Hz; theta, 4–8 Hz; alpha, 8–13 Hz; beta, 13–30 Hz; and gamma, >30 Hz. Study-specific deviations from these boundaries, including alpha or beta sub-bands, were retained rather than recoded and are reported in Table 1. Absolute power, relative power, amplitude, and frequency measures were treated as distinct outcomes. Similarly, global or whole-scalp effects were distinguished from regional or electrode-specific effects, and resting-state, task-related, eyes-open, and eyes-closed findings were not treated as interchangeable.

To minimize selective emphasis on statistically significant findings, outcome selection followed consistent decision rules. When the original study identified a primary EEG outcome or primary post-intervention assessment, that outcome or time point was prioritized in the narrative synthesis. When no primary time point was specified, all eligible post-caffeine assessments were considered and their temporal pattern was described rather than selecting a time point according to statistical significance. Immediate and delayed post-caffeine assessments were therefore considered separately when appropriate. When both global and regional analyses were available, the overall or whole-scalp result was used to characterize the general direction of effect, while regional findings were retained as topographic modifiers. Where several analytical versions of the same EEG outcome were reported, the analysis designated as primary by the original investigators was prioritized; otherwise, adjusted analyses were given greater interpretative weight when adjustment addressed relevant confounding variables.

### 2.7. Risk-of-Bias Assessment

The methodological quality and risk of bias of included studies were assessed according to study design. Risk-of-bias assessment was performed independently by two reviewers (J.C. and A.K.). Each eligible study was assessed by both reviewers using the tool appropriate to its design. The reviewers compared their judgments after completing the assessments independently. Disagreements regarding individual domains or overall risk-of-bias classifications were resolved by discussion and consensus. Randomized controlled trials were evaluated using the revised Cochrane Risk of Bias tool for randomized trials (RoB 2), considering bias arising from the randomization process, deviations from intended interventions, missing outcome data, outcome measurement, and selection of reported results. Non-randomized experimental or observational studies were assessed using ROBINS-I, with attention to confounding, participant selection, intervention classification, deviations from intended exposure, missing data, outcome measurement, and selective reporting. Particular consideration was given to caffeine-specific sources of bias, including inadequate control of prior caffeine intake, insufficient abstinence standardization, absence of placebo control, lack of blinding, variability in caffeine dose, and failure to account for habitual consumption or withdrawal effects.

### 2.8. Data Synthesis

Findings were synthesized narratively because substantial clinical and methodological heterogeneity precluded meaningful quantitative pooling. A meta-analysis was considered both for the overall evidence base and for apparently more homogeneous subgroups, particularly studies examining acute caffeine administration and resting-state spectral EEG. However, even within these narrower groups, studies differed substantially in caffeine dose and formulation, timing of post-ingestion EEG assessment, habitual caffeine consumption and preceding abstinence, participant characteristics, eyes-open versus eyes-closed recording conditions, electrode montages and scalp regions analyzed, preprocessing procedures, and definitions of individual frequency bands. Importantly, nominally similar outcomes were frequently operationalized differently; for example, alpha activity was reported as absolute power, relative power, amplitude, alpha sub-band power, mean or peak frequency, or region-specific/topographic measures. Comparable problems occurred for theta, delta, and beta outcomes. Studies also differed in experimental design, including placebo-controlled crossover protocols, between-group comparisons, caffeine-withdrawal/reintroduction designs, sleep-deprivation paradigms, and pre–post assessments. Consequently, studies that appeared conceptually similar did not provide sufficiently comparable exposure conditions, EEG outcome definitions, and statistical contrasts to support a clinically interpretable pooled effect estimate. Subdivision according to these methodological characteristics would have resulted in very small groups of studies and would not have resolved the underlying differences in outcome definition and measurement. Therefore, meta-analysis, including meta-analysis of nominally homogeneous subgroups, was considered inappropriate, and a structured qualitative narrative synthesis was performed instead. Studies were grouped according to recording condition and EEG outcome domain, including resting-state spectral power and frequency, task-related tonic or oscillatory EEG activity, connectivity-based measures, nonlinear EEG measures, and EEG microstates. Conventional ERP outcomes were not synthesized.

### 2.9. Registration of Review

The review was registered on PROSPERO (CRD420261445522, date of registration: 8 July 2026). The registration occurred before formal screening and data extraction. The PROSPERO record is consistent with the methodology of this review.

## 3. Results

**Figure 1:**
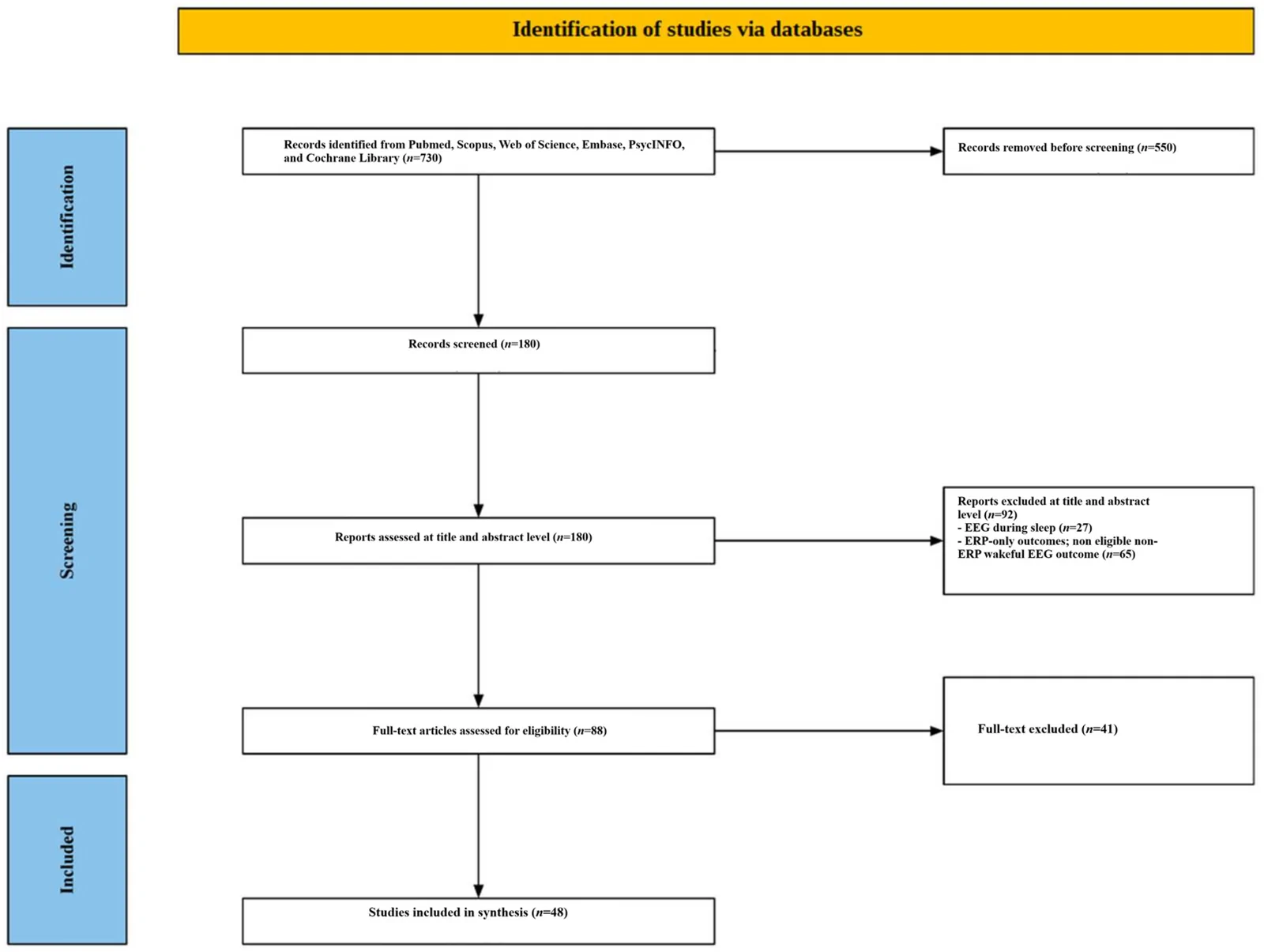
Flow chart depicting the different phases of the systematic review.

Figure 1 provides a summary of the screening process. The search for studies identified 730 publications. 550 duplicates were removed. Titles and abstracts of 180 articles were reviewed. At this stage, 92 records were excluded because they examined EEG exclusively during sleep (*n* = 27) or reported conventional ERP outcomes without any eligible non-ERP wakeful EEG measure (*n* = 65). Eighty-eight reports were assessed for eligibility at full text. Fifty-one were excluded for predefined reasons, including absence of an eligible non-ERP wakeful EEG outcome (*n* = 57), EEG recorded exclusively during sleep (*n* = 28), studies that tested caffeine wth other substances (*n* = 3). A complete article-level list of full-text exclusions, with one primary reason assigned to each report, is provided in Supplementary Table S2. Forty-eight studies were included in the final qualitative synthesis. Ultimately, 48 studies were included in the review [31,32,33,34,35,36,37,38,39,40,41,42,43,44,45,46,47,48,49,50,51,52,53,54,55,56,57,58,59,60,61,62,63,64,65,66,67,68,69,70,71,72,73,74,75,76,77,78]. The included studies are presented in Table 1.

### 3.1. Participants Characteristics

REAcross the 48 included study reports [31,32,33,34,35,36,37,38,39,40,41,42,43,44,45,46,47,48,49,50,51,52,53,54,55,56,57,58,59,60,61,62,63,64,65,66,67,68,69,70,71,72,73,74,75,76,77,78], the final study samples comprised 937 participant-study observations. Individual study samples were generally small, with a median sample size of 16 participants (IQR 12.0–24.25) and a range of 6–56 participants. After accounting for the explicitly reported overlap of one participant between the two trials in study [47] and treating studies [37,68] as reports from the same Canadian schizophrenia cohort, the estimated number of unique participants was approximately 909. The latter deduplication is supported by the virtually identical sample composition, recruitment setting, habitual-caffeine questionnaire values, and experimental procedures reported in [37,68], although the overlap was not explicitly stated by the authors. Study [68] itself inconsistently reports either 25 or 27 participants; the calculation above uses the 27-participant sample reported in its table and corresponding to [37]. Thus, a conservative unique-participant estimate is approximately 909–911.

Participants were predominantly young or early-middle-aged adults. Across the 39 non-duplicated reports for which a mean age could be derived, representing approximately 793 participants, the sample-size-weighted pooled mean age was 25.7 years; the median of the study-level mean ages was 25.3 years. Ages across the entire evidence base ranged from 8 to 55 years. Only one study specifically investigated children, enrolling 30 participants aged 8–13 years [56], and no study specifically recruited older adults aged 65 years or above. Five additional reports included participants as young as 17 years [36,44,51,52,59], including one study focused almost entirely on late adolescents aged 17–19 years [44]; because age-specific counts were not reported in the other studies, the exact number of participants younger than 18 years cannot be determined.

Sex composition varied substantially between studies. Twelve studies (25.0%) were male-only, five (10.4%) were female-only, and 30 (62.5%) included both males and females; sex composition was not reported sufficiently for one study (2.1%). Female-only samples included, among others, the menstrual-cycle study [35], studies of female habitual coffee drinkers [50,71], the smoking study [57], and the low-caffeine female sample in [62]. Male-only studies included several early pharmacological studies as well as the Taekwondo-athlete study [33], sleep-deprivation studies, and studies involving habitual smokers.

The evidence base was overwhelmingly derived from nonclinical populations. Forty-four of 48 reports (91.7%) consisted exclusively of healthy or otherwise nonclinical participants, whereas four reports (8.3%) included psychiatric clinical populations together with healthy controls; no study was restricted exclusively to a clinical psychiatric sample. Psychiatric populations consisted of early-phase schizophrenia/psychosis in [37,68], panic disorder in [55], and generalized anxiety disorder and panic disorder in [78]. Because [37,68] appear to represent the same cohort, these four reports correspond to three unique psychiatric cohorts.

Several studies intentionally recruited populations with characteristics relevant to caffeine responsiveness or vigilance. One study involved competitive athletes, specifically 13 university-level male Taekwondo athletes [33]. Three studies recruited habitual cigarette smokers [57,61,70]. Seven reports involved experimentally sleep-deprived or sleep-restricted participants [41,44,54,58,63,64,76], ranging from partial sleep restriction to approximately 24–40 h of continuous wakefulness. One additional study compared self-reported poor sleepers with normal sleepers [45], but these participants were otherwise healthy and were not classified as a clinical sleep-disorder population.

Habitual caffeine exposure varied markedly across the literature, from caffeine-naïve or very light consumers to individuals habitually consuming several hundred milligrams of caffeine or five or more cups of coffee per day. A single pooled classification of all participants as low, moderate, or high consumers cannot be calculated reliably because studies used substantially different definitions, reported intake in different units (mg/day, mg/kg/day, cups/day, beverages/week, or questionnaire scores), and frequently provided only group means or ranges rather than individual category counts. Among the two studies that explicitly provided participant numbers according to consumption strata, study [43] classified 17 participants as low consumers and 20 as high consumers using a 3 mg/kg/day threshold, whereas study [64] classified 10 as low (0–50 mg/day), 11 as moderate (51–300 mg/day), and 16 as high (>300 mg/day). Accordingly, among the 74 participants for whom explicit categorical counts were available, there were 27 low consumers, 11 moderate consumers, and 36 high consumers. These values should not be interpreted as pooled prevalence estimates because the definitions of habitual consumption differed between the two studies.

### 3.2. EEG Paradigms

The included studies used heterogeneous EEG paradigms to examine the effects of caffeine during wakefulness. The most common approach was resting-state EEG, recorded either with eyes open, eyes closed, or both. Several studies used simple resting recordings before and after acute caffeine administration, usually after oral doses ranging from low doses such as 50 mg to moderate or high doses such as 200 mg, 250 mg, 400 mg, or 500 mg. In these studies, participants were typically seated quietly and EEG was analyzed using spectral power in standard frequency bands, including delta, theta, alpha, and beta [31,34,38,45,48,51,52,55]. Some resting-state studies used only eyes-open recordings, especially when the aim was to assess vigilance or avoid the strong alpha enhancement associated with eye closure [31,32,33,41]. Other studies used eyes-closed recordings, particularly when the investigators were interested in alpha activity, arousal, or caffeine-related suppression of resting alpha power [34,35,45,46]. A third group recorded both eyes-open and eyes-closed EEG, allowing direct comparison of caffeine effects across these two baseline states [36,37,43,45,68].

Several studies explicitly contrasted eyes-open and eyes-closed resting states. In these paradigms, eye condition itself was an important manipulation because opening the eyes strongly reduced alpha activity and increased physiological arousal. One study alternated repeated two-minute eyes-closed and eyes-open resting periods after caffeine or placebo, allowing caffeine effects to be compared with the arousal effect of eye opening [36]. Another study recorded both eyes-open and eyes-closed EEG in early-phase schizophrenia and healthy controls and showed that caffeine effects depended on eye condition, with some alpha and beta effects appearing during eyes-open rest and others during eyes-closed rest [37]. The habitual caffeine-consumption study also recorded five minutes of eyes-open and five minutes of eyes-closed EEG at baseline and again one hour after caffeinated or decaffeinated coffee, making it possible to compare caffeine responses across resting states and habitual intake groups [43].

A second major category consisted of resting EEG combined with cognitive task paradigms. In one study, participants completed both relaxed eyes-open EEG and a concentration performance task before and after placebo, 200 mg caffeine, or 400 mg caffeine. This design showed that caffeine effects during relaxed rest were not identical to caffeine effects during cognitive engagement; for example, slow-wave reductions observed during rest were attenuated or modified during task performance, while caffeine affected task-related occipital power changes [32]. Another study examined dietary caffeine under rested and sleep-restricted conditions across eyes-closed, eyes-open, and task periods, allowing caffeine effects to be compared across baseline vigilance states and recording periods [44]. Task-based EEG was also used in sustained attention paradigms. In the caffeine and L-theanine study, EEG was recorded continuously during the Sustained Attention to Response Task over approximately two hours, and alpha activity was calculated from the prestimulus period before each digit. This paradigm focused on tonic attentional state during active wakefulness rather than resting EEG [77].

Several studies used sleep restriction, sleep deprivation, or prolonged wakefulness paradigms to examine whether caffeine counteracts EEG markers of sleep pressure. One study recorded waking EEG during 40 h of sustained wakefulness and administered two 200 mg caffeine doses, one after 11 h and another after 23 h of wakefulness. Waking theta activity was measured during eyes-open recordings and used as an objective marker of sleep-pressure accumulation [41]. Other studies examined caffeine after acute sleep deprivation or prolonged wakefulness, including paradigms using 400 mg caffeine after sleep deprivation [54], 600 mg slow-release caffeine during overnight or prolonged sleep loss [58], and caffeine-sensitive versus caffeine-insensitive responses during extended wakefulness [76]. These paradigms differed from ordinary resting-state studies because the EEG outcome was interpreted against a background of rising sleep pressure, reduced vigilance, and increased slow-wave activity.

Driving and monotonous vigilance paradigms were another subgroup. In an early-morning driving study, EEG was recorded during simulated or monotonous driving after partial sleep restriction or total sleep deprivation. The main EEG outcome was combined theta–alpha activity in the 4–11 Hz range, used as a marker of drowsiness during sustained driving [63]. Another study examined chronically mildly sleep-restricted drivers and compared caffeine chewing gum, bright light, their combination, and placebo during a driving-related vigilance paradigm. This design tested whether caffeine could reduce EEG signs of drowsiness in a more ecologically relevant setting, although the caffeine effect was weaker than the bright-light effect for theta activity [72].

Some studies used withdrawal, abstinence, or chronic-maintenance paradigms rather than simple acute caffeine administration. One withdrawal study recorded qEEG during normal caffeine consumption, then after one, two, and four days of caffeine abstinence, and again after caffeine reintroduction in a subset of participants. This design allowed the investigators to examine EEG changes produced by caffeine withdrawal and their reversal after caffeine was consumed again [40]. Another study used a longer crossover maintenance design, in which regular caffeine users completed chronic caffeine and chronic placebo phases, followed by acute caffeine or placebo challenge sessions within each phase. This paradigm was designed to separate acute caffeine effects from withdrawal reversal and tolerance [42]. A related dietary caffeine study also attempted to control for withdrawal and withdrawal reversal when assessing whether caffeine had genuine acute or chronic effects on EEG [59].

A smaller number of studies used sensory-stimulation or task paradigms that also incorporated event-related measures. In the present review, only their eligible non-ERP EEG outcomes were synthesized. The photic-stimulation study [49] examined spontaneous EEG power and steady-state photic-driving spectral responses. The visual selective-attention study [67] additionally collected ERP measures; however, only its background and prestimulus spectral EEG findings were included in the present synthesis, whereas the ERP findings were outside the scope of the review. Other task-related studies included attention-to-threat paradigms with frontal theta/beta ratio analysis [62], sustained attention tasks [77], and pharmacodynamic studies including psychomotor or cognitive testing alongside EEG [45,47,50,75].

Several studies used specialized EEG paradigms or analysis frameworks. One study assessed nonlinear EEG complexity during cognitive task performance using correlation dimension, making the paradigm focused on dynamic signal complexity rather than conventional frequency-band power [65]. Another used resting-state EEG microstate analysis in early-phase schizophrenia and healthy controls after caffeine or placebo, examining the duration, occurrence, and contribution of microstate classes during eyes-open and eyes-closed rest [68]. A wearable EEG pilot study combined resting EEG with machine-learning analysis of pre- and post-caffeine states, using band-power features from a small number of electrodes to classify caffeine-related EEG changes [39]. Another study combined EEG with fMRI and related caffeine-induced EEG changes to global BOLD signal and vigilance measures, providing a multimodal resting-state paradigm rather than a purely EEG-only design [73].

Overall, the EEG paradigms varied substantially across the included studies. Most evidence came from resting-state spectral EEG, especially eyes-open and/or eyes-closed recordings before and after acute caffeine administration. However, a substantial subset of studies examined caffeine under conditions of cognitive task performance, sustained attention, sleep deprivation, prolonged wakefulness, driving-related drowsiness, withdrawal, chronic caffeine maintenance, sensory stimulation, EEG microstates, nonlinear EEG complexity, or multimodal EEG–fMRI vigilance assessment. This methodological diversity is important because caffeine effects were often paradigm-dependent. Resting-state studies commonly emphasized alpha suppression or broad EEG desynchronization, prolonged-wakefulness studies emphasized theta or slow-wave attenuation, driving paradigms focused on theta–alpha drowsiness activity, withdrawal paradigms showed increased slow-wave activity during abstinence and partial normalization after caffeine reintroduction, and task paradigms often revealed more selective or state-dependent EEG effects rather than uniform changes across all frequency bands.

### 3.3. Delta Activity

Delta activity showed heterogeneous responses to acute caffeine administration under non-sleep-deprived conditions, and the available evidence does not support a consistent reduction in waking delta power. Studies involving caffeine withdrawal or reintroduction, experimentally induced sleep deprivation or prolonged wakefulness, chronic or habitual exposure comparisons, coffee-based interventions, and caffeine combined with other interventions are considered separately in the exposure-specific sections below.

In an early topographic qEEG study using 200 mg caffeine in healthy young men, delta activity was not among the frequency bands most clearly affected by caffeine. Although total EEG power decreased during eyes-open rest, the band-specific changes were concentrated primarily in alpha and beta ranges, with relatively few significant delta effects [31]. A more evident delta response was observed in the study comparing 200 and 400 mg caffeine. During relaxed eyes-open rest, caffeine reduced spectral power, and after 400 mg the decrease became more generalized within the delta range. However, this effect was not maintained during the concentration task, indicating that even under acute administration the delta response depended strongly on behavioral state [32].

A clearer acute reduction in delta activity was reported in university-level taekwondo athletes given 200 mg caffeine. Delta power decreased after supplementation, particularly at frontal and posterior midline electrodes, including Fpz, Fz, Cz, Pz, POz, and Oz. However, after the subsequent combined mental and physical fatigue protocol, delta activity partially rebounded and the caffeine-related reduction was no longer clearly sustained [33]. In contrast, 50 mg caffeine did not produce a specific delta effect in healthy young adults: parietal and occipital delta power decreased over time in both caffeine and placebo conditions, suggesting a procedural or time-related change rather than a pharmacological effect [34].

Habitual caffeine intake also complicated the interpretation of some nominally acute experiments. In the menstrual-cycle study, caffeine initially appeared to reduce parietal delta power, but this effect disappeared after habitual weekly caffeine intake was included as a covariate. The apparent reduction therefore could not be considered a robust independent acute caffeine effect [35]. Similarly, in participants with early-phase schizophrenia and healthy controls, 200 mg caffeine did not significantly alter delta power during either eyes-open or eyes-closed resting EEG [37]. Although additional associations with habitual intake and withdrawal symptoms were reported in that study, these are not considered evidence of a direct acute delta effect and are therefore interpreted separately from the acute-administration findings.

Several other acute-administration studies likewise found little evidence for a reproducible delta response. In poor and normal sleepers receiving 2.5 mg/kg caffeine, resting delta power did not change significantly after caffeine in either group, despite baseline electrophysiological differences between the sleeper phenotypes [45]. In the panic-disorder study, caffeine did not significantly alter central delta amplitude [55]. These null findings indicate that acute caffeine does not necessarily suppress delta activity even in populations differing in baseline arousal or clinical characteristics.

Some acute studies nevertheless reported decreases in delta activity in more specific populations. In sleep-sensitive and non-sensitive regular coffee consumers, 140 mg caffeine reduced slower EEG activity, including delta power, across the sample. The reduction appeared to persist longer in caffeine-sensitive participants, suggesting that individual caffeine sensitivity may modify the duration of the electrophysiological response [46]. In children, 80 mg caffeine also reduced delta power, but this effect was topographically restricted, particularly to a left-posterior region, rather than representing a generalized suppression across the scalp [56].

A multimodal resting-state EEG–fMRI study provided additional evidence that acute caffeine-related vigilance changes can involve delta activity. Caffeine-related reductions in the fMRI global signal during eyes-closed rest were associated with reduced slow-wave EEG activity, including delta, whereas the relationship was weaker during eyes-open recordings [73]. This finding suggests that acute delta modulation may emerge particularly when delta covaries with fluctuations in vigilance, but it does not establish a uniform direct suppression of delta power across waking states.

A small number of acute findings were opposite to the expected stimulant-like pattern. In an older psychopharmacological study, 100 mg caffeine produced a transient increase in delta-band energy approximately 2.8 h after administration, with no significant EEG effect remaining by approximately 6.1 h [47]. This result further demonstrates that delta does not show a simple unidirectional response to acute caffeine exposure.

Overall, the direct acute-administration evidence indicates that delta activity is not a stable or universal EEG marker of caffeine during ordinary wakefulness. Some studies demonstrated reductions in delta power, particularly at higher doses or in specific populations and recording conditions [32,33,46,56], whereas several others found weak, focal, transient, or absent effects [31,34,35,37,45,47,55]. Therefore, the evidence from this exposure category supports a context-dependent rather than generalized acute delta-suppressing effect. Stronger evidence for attenuation of delta under elevated sleep pressure derives from the separate sleep-deprivation and prolonged-wakefulness literature and should not be used to strengthen conclusions regarding acute caffeine effects in rested participants.

Certainty of evidence is low. Confidence in a direct acute effect of caffeine on waking delta activity is limited by inconsistent direction and magnitude of effects, small samples, heterogeneity in dose, eye condition, participant characteristics and EEG methodology, and risk-of-bias concerns across several contributing studies. Accordingly, the evidence supports only a low-certainty conclusion that acute caffeine can reduce delta activity under selected conditions rather than a general delta-suppressing action.

Among 12 studies contributing direct acute, non-sleep-deprived delta outcomes, three reported a predominant decrease in delta activity [33,46,56], one reported an increase [47], five reported no significant caffeine-related effect [31,34,37,45,55], and three produced mixed or condition-dependent findings [32,35,73]. The principal experimental context was resting-state EEG or qEEG, with variability arising particularly from task engagement, eye condition, habitual caffeine intake, participant phenotype, and vigilance state. The risk-of-bias profile was mixed but unfavorable: seven studies were judged to have some concerns under RoB 2, two were at high RoB 2 risk, two non-randomized studies had serious ROBINS-I risk, and one had critical ROBINS-I risk. Thus, five of the 12 contributing studies had high, serious, or critical overall risk of bias, reinforcing the interpretation of delta attenuation as a context-dependent and low-certainty effect rather than a general acute caffeine signature.

Certainty of evidence is low. Although several studies indicated that caffeine reduces waking delta activity, particularly during fatigue, sleep deprivation, low vigilance, or low habitual caffeine intake, the direction and magnitude of the effect were not consistent across studies. In addition, the relevant evidence included studies with high, serious, or critical risk-of-bias concerns as well as numerous randomized studies with some concerns. Therefore, confidence is limited in a generalized delta-reducing effect of caffeine. The evidence is more convincing for the narrower interpretation that caffeine may attenuate delta activity when delta reflects elevated sleep pressure or reduced vigilance.

### 3.4. Theta Activity

Theta activity showed heterogeneous responses to acute caffeine administration under non-sleep-deprived conditions. Although several studies reported reductions in theta power, the effect was considerably less consistent than the acute reduction in alpha activity and depended on dose, behavioral state, participant characteristics, recording condition, and EEG methodology. Studies examining caffeine during sleep deprivation or prolonged wakefulness, caffeine withdrawal and reintroduction, chronic or habitual exposure, coffee-based interventions, and caffeine combined with other interventions are considered separately in the exposure-specific sections and are not used here as evidence for a direct acute theta-suppressing effect.

In an early topographic qEEG study using 200 mg caffeine in healthy young men, theta was not among the frequency bands most strongly affected. Caffeine reduced total EEG power during eyes-open rest, but frequency-specific reductions were more prominent in alpha1, alpha2, and beta1 activity, with comparatively limited theta changes [31]. In contrast, a study comparing 200 and 400 mg caffeine demonstrated a clearer theta response during relaxed eyes-open rest. Both doses reduced spectral power in the theta and alpha ranges across multiple electrodes. However, this effect depended on behavioral state: during the concentration task, the resting theta reduction was no longer apparent and theta power tended instead to increase more during task performance after caffeine. Thus, the effect of acute caffeine on theta could not be characterized as uniform suppression across resting and cognitively engaged states [32].

Several other acute studies failed to demonstrate a robust theta effect. In university-level taekwondo athletes, 200 mg caffeine reduced delta power but did not significantly alter theta activity [33]. Similarly, 50 mg caffeine did not produce a caffeine-specific theta response; although theta changed over time, comparable changes occurred after placebo, indicating that they were more likely related to the experimental procedure or passage of time than to caffeine itself [34]. In the menstrual-cycle study, caffeine initially appeared to reduce theta power, particularly over parietal regions, but this effect disappeared after habitual weekly caffeine intake was included as a covariate. The apparent theta reduction therefore could not be regarded as a robust independent acute caffeine effect [35].

In early-phase schizophrenia and healthy controls, acute administration of 200 mg caffeine did not significantly change theta power during eyes-closed resting EEG. Associations were nevertheless observed between symptom severity and caffeine-related theta changes within the schizophrenia group. However, because withdrawal symptoms were also associated with slow-frequency activity, these correlations could not be interpreted straightforwardly as evidence of a general acute pharmacological reduction in theta power [37]. The direct group-level finding from this study therefore remains essentially negative for theta activity.

Other resting-state studies produced similarly weak or absent findings. In poor and normal sleepers, caffeine at 2.5 mg/kg did not significantly modify resting theta power. Although poor sleepers had lower baseline theta activity than normal sleepers, this difference was not substantially altered by caffeine [45]. In contrast, sleep-sensitive and non-sensitive regular coffee consumers given an acute 140 mg caffeine challenge showed reductions in slower activity, including theta power, together with increases in faster activity. The theta reduction occurred across the sample and was interpreted as part of a shift toward increased vigilance, although the duration of the electrophysiological response differed according to caffeine sensitivity [46].

Older psychopharmacological studies likewise demonstrated substantial variability. In a study comparing caffeine with cyclizine, theta energy was lower after caffeine than after the sedating cyclizine condition at the corresponding measurement point. However, because the difference was driven partly by comparison with a sedative drug, it provides weaker evidence for an absolute theta-suppressing action of caffeine itself [47]. By contrast, a dose study using 250 and 500 mg caffeine found one of the clearest acute theta effects. Log theta power in the 4–7.5 Hz range decreased after caffeine in a dose-related manner, with the effect persisting across the five-hour observation period and being particularly pronounced after the 500 mg dose [48]. This study therefore provides comparatively strong direct evidence that sufficiently high acute doses can suppress theta activity during wakefulness.

Not all experimental paradigms reproduced this effect. During photic stimulation, caffeine did not significantly alter EEG power at 5 Hz, providing no evidence for a reliable theta-range effect in that paradigm [49]. Similarly, in a dose-response study of regular female coffee drinkers receiving isolated caffeine, relative theta power did not significantly change; the principal EEG response involved acceleration of dominant alpha and beta frequencies rather than redistribution of theta power [50]. In another resting-state arousal study, caffeine clearly reduced alpha power and increased mean alpha frequency, whereas theta activity did not change significantly [51]. A time-course study also suggested somewhat lower delta and theta power at selected post-caffeine measurements, but these changes were not statistically robust, with alpha suppression representing the principal EEG correlate of arousal [52].

More definite acute theta reductions were observed in selected clinical and developmental samples. In participants with panic disorder and healthy controls, caffeine reduced central theta amplitude, whereas theta tended to increase after placebo. This pattern was compatible with increased alertness after caffeine, although the small clinical sample limits generalization [55]. In children, 80 mg caffeine produced a global reduction in theta power. However, the relationships between theta and skin conductance were weak and inconsistent, leading the authors to suggest that the response might partly reflect developmental characteristics of childhood EEG rather than a simple adult-like index of generalized arousal [56].

A further acute study examining frontal theta in relation to attention to threat found that 200 mg caffeine significantly reduced frontal theta power compared with placebo [62]. Importantly, however, caffeine simultaneously reduced frontal beta power, so the theta/beta ratio itself remained unchanged. Thus, this study supports an acute effect on frontal theta power but not a generalized shift in the balance between theta and beta activity. The theta/beta ratio findings are considered separately in the dedicated outcome section.

Overall, the direct acute-administration evidence provides only limited support for a general theta-suppressing action of caffeine during ordinary wakefulness. Clear reductions were reported in selected studies, particularly at moderate-to-high doses or in specific participant groups [32,46,48,55,56,62], whereas several other experiments found absent, weak, comparator-dependent, or behaviorally state-dependent effects [31,33,34,35,37,45,47,49,50,51,52]. Therefore, acute caffeine should not be described as consistently reducing theta power in rested or otherwise non-sleep-deprived participants. The substantially more reproducible attenuation of theta observed when caffeine counteracts experimentally elevated sleep pressure belongs to the separate sleep-deprivation/prolonged-wakefulness evidence category, while normalization of increased theta after abstinence represents withdrawal reversal and should likewise not be interpreted as equivalent to a direct acute pharmacological effect.

Among 17 studies contributing direct acute, non-sleep-deprived theta outcomes, five reported a predominant decrease in theta activity [46,48,55,56,62], none reported a predominant increase, nine reported no significant effect [31,33,34,37,45,49,50,51,52], and three showed mixed or condition-dependent findings [32,35,47]. The main experimental context was resting-state EEG in rested participants, supplemented by selected clinical, developmental, and task-related paradigms. Clearer acute theta reductions were generally observed at moderate-to-high doses or in specific participant groups, whereas null findings were frequent in ordinary resting-state recordings. Twelve contributing studies had some concerns under RoB 2, two were at high RoB 2 risk, two non-randomized studies had serious ROBINS-I risk, and one had critical ROBINS-I risk. Accordingly, the direct acute evidence remains heterogeneous and should be distinguished from the more consistent theta attenuation observed during sleep deprivation, prolonged wakefulness, or withdrawal reversal.

Certainty of evidence is low. The direct acute evidence is limited by inconsistent findings, generally small samples, variation in caffeine dose, resting versus task conditions, participant phenotype, theta-band definitions, and risk-of-bias concerns. Accordingly, confidence is low that acute caffeine produces a generalized decrease in theta activity during ordinary wakefulness. The stronger and more consistent evidence that caffeine attenuates theta under sleep deprivation, prolonged wakefulness, or withdrawal should be assigned to those specific exposure categories rather than used to increase the certainty of the direct acute effect.

### 3.5. Alpha Activity

Among studies examining acute caffeine administration under non-sleep-deprived conditions, alpha activity was one of the most consistently affected EEG frequency bands. The predominant finding was a reduction in alpha power or amplitude, although the magnitude, topography, and affected alpha sub-band varied across studies. Several experiments also reported increases in mean, peak, or dominant alpha frequency. However, neither alpha suppression nor alpha-frequency acceleration was universal, and some studies reported absent, restricted, or state-dependent effects. Studies of caffeine withdrawal and reintroduction, chronic or habitual exposure, coffee-based interventions, sleep deprivation or prolonged wakefulness, and caffeine combined with other interventions are considered separately in the exposure-specific sections.

In an early topographic qEEG study of healthy young men, 200 mg caffeine significantly reduced absolute EEG power, with particularly prominent decreases in alpha1 and alpha2 activity during eyes-open rest. Alpha1 reductions were apparent across several regions, including frontal and occipital sites, without a sharply localized topographic pattern [31]. Similarly, in a study comparing 200 and 400 mg caffeine, alpha power decreased during relaxed eyes-open rest. After 200 mg, theta and alpha reductions were apparent across multiple electrodes, whereas after 400 mg the alpha2 decrease became more spatially widespread, including temporo-central regions. During a concentration task, caffeine also attenuated the task-related occipital increase in spectral power, particularly in the alpha range, although this effect was stronger after 200 mg than after 400 mg [32].

Not all acute studies demonstrated alpha suppression. In university-level taekwondo athletes, 200 mg caffeine significantly reduced delta power but did not significantly modify alpha activity [33]. By contrast, a low-dose experiment using 50 mg caffeine demonstrated a relatively selective alpha effect. Alpha power normally increased over time during eyes-closed rest after placebo, particularly at frontopolar, central, parietal, and occipital sites, whereas this increase was suppressed after caffeine. Percentage changes in alpha power were consequently significantly lower after caffeine at Fpz, Fz, Cz, Pz, and Oz [34]. In a study examining menstrual-cycle phase, 200 mg caffeine did not produce a uniform reduction in total alpha power. The principal effect involved alpha1 activity, particularly in parietal regions, whereas total alpha and alpha2 power were not significantly altered. Adjustment for habitual weekly caffeine intake modified the magnitude of the observed response, indicating that habitual use may moderate the electrophysiological expression of an acute caffeine challenge [35].

Several studies provided more direct evidence for broadly distributed alpha suppression. When caffeine and eye opening were examined as separate arousal manipulations, 250 mg caffeine significantly reduced alpha amplitude relative to placebo. The effect was global and additive to the much larger alpha attenuation produced by eye opening, with caffeine alone producing approximately a 12% reduction from the eyes-closed placebo condition [36]. In participants with early-phase schizophrenia and healthy controls, 200 mg caffeine reduced alpha1 power during eyes-open rest in both groups, particularly frontally. Alpha2 power was also reduced, although the distribution differed between groups. During eyes-closed recordings, alpha1 was not significantly altered, while frontal alpha2 reduction was observed in the schizophrenia group [37]. In another crossover study, 400 mg caffeine significantly decreased absolute EEG power across anterior and posterior regions, with alpha reduction representing one of the clearest qEEG effects [38].

Clinical and individual-difference studies further demonstrated that acute alpha responses depend on participant characteristics. In poor and normal sleepers, 2.5 mg/kg caffeine did not significantly alter resting alpha power or peak alpha frequency, despite baseline differences between the two groups [45]. In sleep-sensitive and non-sensitive regular coffee consumers, 140 mg caffeine produced a different pattern from the more common alpha-suppression response, increasing faster-frequency activity that included alpha and beta power while reducing slower delta and theta activity [46]. An older psychopharmacological study likewise found no significant acute alpha effect, with the principal EEG response instead consisting of a transient increase in delta-band energy [47]. In contrast, both 250 and 500 mg caffeine reduced alpha power in the 8–13.5 Hz range in another acute dose study, although the response was not clearly dose-dependent and the effect of the lower dose appeared to dissipate more rapidly [48].

Acute caffeine also influenced alpha-range activity in sensory and arousal paradigms. During photic stimulation, caffeine reduced spontaneous EEG power and attenuated the spectral response to 10-Hz visual stimulation, indicating suppression of both spontaneous and visually driven alpha-range activity rather than enhancement of the photic response [49]. In a dose–response study of regular female coffee drinkers receiving isolated caffeine, relative alpha power did not change significantly. Instead, dominant alpha frequency increased after the highest dose of 6.0 mg/kg, suggesting that caffeine may accelerate the alpha rhythm without necessarily decreasing its relative power [50].

A separate resting-state arousal study showed both effects simultaneously. Following 250 mg caffeine, global alpha power decreased from approximately 21.3 µV^2^ under placebo to 16.2 µV^2^ after caffeine, while mean alpha frequency increased from 10.16 to 10.30 Hz [51]. In a related time-course experiment, alpha power was the clearest EEG correlate of the developing caffeine response. Significant alpha suppression emerged approximately 25–33 min after ingestion, corresponded temporally with increased skin conductance, temporarily weakened after an intervening task, and subsequently reappeared [52].

Acute caffeine also altered alpha activity in several clinical and developmental samples. In participants with panic disorder and healthy controls, caffeine increased peak occipital alpha frequency by approximately 0.32 Hz at 30 min and 0.40 Hz at 75 min and simultaneously reduced alpha amplitude, particularly in the slow-alpha range. Differences in slow-alpha reduction between groups were largely attributable to baseline amplitude differences rather than a diagnosis-specific pharmacological response [55]. In children, 80 mg caffeine produced a global reduction in alpha power and a small increase in mean alpha frequency, from approximately 9.74 to 9.81 Hz, with the frequency effect more pronounced posteriorly than frontally [56].

Additional acute qEEG evidence was consistent with alpha attenuation. In a study using caffeine sodium benzoate, caffeine reduced alpha1 power along the temporal arch together with reductions in slower activity and increases in beta2/gamma coherence [60]. EEG cartography similarly showed that caffeine increased mean alpha frequency by up to approximately 0.34 Hz in 11 of 16 channels while decreasing mean alpha RMS amplitude in 11 channels and reducing relative alpha percentage in seven channels. This combination of weaker and faster alpha activity was interpreted as a desynchronized waking EEG profile [66]. During visual selective attention, caffeine reduced background EEG power most strongly in the lower alpha range, with smaller decreases in higher alpha activity, but did not significantly alter dominant alpha peak frequency [67].

Acute effects remained heterogeneous in some additional populations and paradigms. In anxiety-disorder groups and healthy controls, 250 and 500 mg caffeine generally reduced alpha-range EEG power, although the magnitude of the response differed across diagnostic groups and eye conditions [78]. In a coping-style study, 3.3 mg/kg caffeine increased dominant alpha frequency, although one subgroup showed an opposite response, indicating substantial individual variability [71]. In a simultaneous EEG–fMRI vigilance experiment, caffeine-related increases in vigilance were associated with increased rather than decreased alpha activity together with reduced slow-wave activity, representing an important exception to the more typical alpha-suppression pattern [73]. Another dose study found that both 250 and 500 mg caffeine reduced EEG amplitude within the alpha range and across the broader 4–30 Hz spectrum, particularly at central and parietal leads, without a clear dose-response relationship [74]. Finally, in a study comparing caffeine with alcohol and diphenhydramine, 200 mg caffeine did not significantly alter resting EEG but reduced parietal alpha power during task performance, indicating that the alpha effect could emerge during cognitive engagement despite a negative resting-state result [75].

Overall, the direct acute-administration evidence supports alpha suppression as the most reproducible conventional spectral response to caffeine under non-sleep-deprived wakefulness, particularly as reduced alpha power or amplitude during resting or task-related recordings [31,32,34,36,37,38,48,51,52,55,56,60,66,67,74,75,78]. A second recurring acute finding was acceleration of the alpha rhythm, including increases in mean, peak, or dominant alpha frequency [50,51,55,56,66,71]. Nevertheless, several studies reported no significant alpha effect or responses confined to particular sub-bands, eye conditions, populations, or behavioral states [33,35,45,46,47,73]. Thus, the direct acute evidence indicates that caffeine commonly reduces alpha synchronization and may increase alpha frequency, but these effects remain dependent on dose, recording state, alpha metric, and individual characteristics. Evidence derived from withdrawal reversal, sleep deprivation, habitual-intake comparisons, coffee exposure, or combined interventions should be interpreted separately and does not contribute directly to this conclusion.

Among 25 direct acute, non-sleep-deprived studies contributing alpha power or amplitude outcomes, 17 reported a predominant decrease [31,32,34,36,37,38,48,49,51,52,55,56,60,66,67,74,78], two reported an increase [46,73], four reported no significant effect on alpha power [33,45,47,50], and two showed mixed or condition-dependent findings [35,75]. The predominant experimental context was resting-state eyes-open or eyes-closed EEG, although reductions were also observed in arousal, clinical, developmental, and task-related paradigms. The principal exceptions involved participant-specific or state-specific responses, including caffeine sensitivity and vigilance-related EEG–fMRI associations. Eighteen studies had some concerns under RoB 2, three had high RoB 2 risk, three non-randomized studies had serious ROBINS-I risk, and one had critical ROBINS-I risk. Despite these limitations, the clear numerical predominance of decreases supports alpha suppression as the most reproducible conventional spectral effect of acute caffeine in the present review.

Certainty of evidence is moderate. Alpha activity is supported by a larger and more internally consistent acute evidence base than the other conventional EEG frequency bands, with repeated reductions in alpha power or amplitude across independent studies and several replications of alpha-frequency acceleration. However, confidence is limited by generally small samples, heterogeneity in caffeine dose, eye condition, EEG methodology, alpha-band definitions, participant characteristics, and the presence of risk-of-bias concerns in the contributing studies. The evidence therefore supports with moderate certainty the conclusion that acute caffeine commonly suppresses alpha power or amplitude during wakefulness, while certainty regarding alpha-frequency acceleration, topographic specificity, and individual moderators is lower.

### 3.6. Beta Activity

Beta activity showed highly heterogeneous responses to acute caffeine administration under non-sleep-deprived conditions. Unlike alpha activity, which more frequently decreased after caffeine, beta power did not show a reproducible unidirectional response. Across direct acute-administration studies, beta activity decreased in some experiments, increased in others, and frequently remained unchanged. The direction of the effect appeared to depend on beta sub-band definition, dose, recording condition, participant characteristics, and whether the outcome was expressed as absolute or relative power, amplitude, or dominant frequency. Studies involving caffeine withdrawal or reintroduction, chronic or habitual exposure, coffee-based interventions, experimentally induced sleep deprivation or prolonged wakefulness, and caffeine combined with other interventions are considered separately in the exposure-specific sections.

In an early topographic qEEG study of healthy young men, 200 mg caffeine reduced absolute power primarily in the alpha and slow-beta ranges. The beta effect was most evident for beta1 activity during eyes-open rest, where reductions occurred at several electrode positions, whereas beta2 showed fewer significant changes. Thus, this experiment did not support a generalized increase in fast-frequency activity; instead, acute caffeine reduced slow-beta power together with alpha activity [31]. In the study comparing 200 and 400 mg caffeine during relaxed rest and concentration-task performance, beta was not identified as a principal caffeine-sensitive band, with the main spectral effects involving slower and alpha-range activity [32].

Several additional acute experiments reported no clear beta effect. In university-level taekwondo athletes, 200 mg caffeine did not significantly alter beta activity despite a significant reduction in delta power [33]. Similarly, after 50 mg caffeine, beta power showed time-related changes, but these changes were broadly comparable between caffeine and placebo conditions and were not considered caffeine-specific [34]. In the menstrual-cycle study, an apparent reduction in global beta power disappeared after habitual weekly caffeine intake was entered as a covariate, indicating that the initial effect could not be regarded as a robust independent response to the acute caffeine challenge [35].

In early-phase schizophrenia and healthy controls, acute caffeine produced beta reductions, but these depended strongly on diagnostic group and eye condition. During eyes-open rest, caffeine reduced beta power overall, with the effect driven mainly by healthy controls, who showed lower frontal and parietal beta power after caffeine than placebo. During eyes-closed rest, frontal beta activity decreased in both groups. Within the schizophrenia group, greater auditory-hallucination severity was associated with smaller caffeine-related beta changes, suggesting that clinical characteristics may moderate beta responsiveness [37]. These findings support an acute beta effect in some participants but do not indicate a generalized beta-enhancing action of caffeine.

Individual caffeine sensitivity was associated with a different response. In sleep-sensitive and non-sensitive regular coffee consumers given an acute 140 mg caffeine challenge, fast-frequency activity increased, particularly in the beta range. Beta2 power increased from approximately 13.9% at baseline to more than 20% after caffeine. In non-sensitive participants the increase subsequently returned toward baseline, whereas in caffeine-sensitive individuals elevated beta activity persisted for longer, including approximately five hours after ingestion [46]. This study therefore provides evidence that acute caffeine can increase beta power, but also illustrates substantial interindividual variability in the duration of the response.

Older psychopharmacological studies further demonstrated the lack of a simple beta response. In a comparison of caffeine and cyclizine, caffeine did not significantly alter beta activity and failed to produce a clear stimulant-like enhancement of faster EEG frequencies [47]. In another study using 250 and 500 mg caffeine, beta responses were complex and non-monotonic: the 500 mg dose produced less beta change than the 250 mg dose. The authors proposed that competing processes—including broad reductions in EEG power and stimulant-related enhancement of faster activity—could contribute to this apparently paradoxical dose-response pattern [48]. In the photic-stimulation experiment, caffeine generally reduced EEG power and attenuated visually driven spectral responses rather than producing a broad increase in beta-range cortical activity [49].

Studies distinguishing beta power from beta frequency provided an important additional finding. In regular female coffee drinkers receiving experimentally administered caffeine, relative beta power did not significantly change, but dominant beta frequency increased after both 3.0 and 6.0 mg/kg doses [50]. Thus, acute caffeine could accelerate the beta rhythm without increasing its spectral contribution. In another resting-state arousal study, caffeine did not significantly alter beta power in the principal analyses despite clear effects on alpha power and alpha frequency [51]. These findings indicate that beta-frequency acceleration and beta-power enhancement should not be treated as equivalent electrophysiological responses.

Some acute studies reported beta suppression rather than enhancement. In a time-course arousal experiment, beta power decreased following caffeine, becoming significant approximately 29–33 min after ingestion. The reduction temporarily disappeared after an intervening task and subsequently re-emerged. Unlike the more global alpha suppression observed in the same experiment, the beta decrease was topographically restricted, particularly to right-frontal and posterior-lateral regions, suggesting a more selective electrophysiological response [52].

Clinical and developmental studies also tended to show decreases rather than stimulant-like beta enhancement. In participants with panic disorder and healthy controls, caffeine reduced central beta amplitude across slow-, medium-, and fast-beta components, together with reductions in slow-alpha and theta amplitudes [55]. In children, 80 mg caffeine also reduced beta power, although the effect was focal and topographically non-uniform, making it less compatible with a generalized arousal interpretation [56].

An acute qEEG study using caffeine sodium benzoate demonstrated a more localized beta-enhancing response. Caffeine increased beta2 power principally at temporal derivations F8 and T3 while simultaneously increasing beta2 and gamma coherence across a wider cortical distribution [60]. Therefore, the local increase in beta2 power formed part of a broader alteration in high-frequency synchronization rather than a generalized increase in beta amplitude across the scalp. The coherence findings are considered in greater detail in the dedicated connectivity section.

In a study of attention to threat, 200 mg caffeine did not significantly alter the frontal theta/beta ratio, but separate spectral analyses showed a significant reduction in frontal beta power compared with placebo [62]. Because frontal theta power also decreased, the reduction in beta did not translate into a significant change in the composite theta/beta ratio. Thus, this experiment provides additional evidence that acute caffeine may reduce beta power without producing the stereotypical increase in fast-frequency activity often assumed for stimulants.

Further evidence of beta suppression was found in anxiety-disorder groups and healthy controls, where 250 and 500 mg caffeine generally decreased EEG power in both alpha and beta ranges [78]. In a coping-style study, the result depended on how beta activity was quantified: caffeine increased dominant beta frequency, consistent with acceleration of the ongoing rhythm, but decreased relative beta power. Moreover, responses differed among coping-style subgroups [71]. This dissociation again indicates that faster beta frequency does not necessarily imply greater beta power.

In a simultaneous EEG–fMRI vigilance study, beta activity was primarily examined as an electrophysiological correlate of global-signal fluctuations rather than as a principal caffeine-responsive power measure. Although caffeine shifted the broader vigilance-related EEG profile, the study did not provide clear evidence for a direct caffeine-induced increase in beta power [73]. Finally, in another acute dose study, both 250 and 500 mg caffeine reduced EEG amplitude across the 4–30 Hz range and specifically within the alpha and beta ranges, particularly over central and parietal leads [74].

Overall, direct acute caffeine administration does not produce a consistent increase in beta power during ordinary wakefulness. Beta reductions were reported in several studies involving resting-state qEEG, clinical populations, time-course arousal paradigms, developmental samples, attention tasks, and broad-band amplitude analyses [31,37,52,55,56,62,74,78]. Conversely, beta enhancement occurred in selected experiments, particularly as increased beta2 activity [46,60], while several studies reported no significant beta-power effect [32,33,34,35,45,47,50,51]. Moreover, some experiments demonstrated increased dominant beta frequency without increased beta power [50,71]. The acute evidence therefore supports beta activity as a context- and metric-dependent EEG outcome rather than a reproducible marker of caffeine-induced cortical activation. Beta increases observed in sleep deprivation, caffeine withdrawal reversal, habitual-intake comparisons, coffee interventions, or nicotine/smoking paradigms should be interpreted within those respective exposure categories rather than used to support a generalized acute beta-enhancing effect.

Among 20 studies contributing direct acute beta-domain outcomes, eight reported a predominant decrease in beta power or amplitude [31,37,52,55,56,62,74,78], two reported a predominant increase [46,60], eight reported no significant beta-power effect [32,33,34,35,45,47,50,51], and two produced mixed or metric-dependent findings [48,71]. In the latter studies, beta responses varied non-monotonically with dose or differed according to whether beta was quantified as spectral power or dominant frequency. The principal experimental contexts included resting-state qEEG, clinical and developmental recordings, arousal paradigms, and frontal task-related EEG. Thirteen contributing studies had some concerns under RoB 2, two had high RoB 2 risk, four non-randomized studies had serious ROBINS-I risk, and one had critical ROBINS-I risk. The approximately balanced distribution of decreases, null findings, and smaller numbers of increases or mixed effects supports beta activity as a context- and metric-dependent outcome rather than a reproducible marker of acute caffeine-induced activation.

Certainty of evidence is low. Confidence in the direction of the acute caffeine effect on beta activity is limited by substantial inconsistency, variation in beta-band definitions and outcome metrics, small sample sizes, differences in dose and recording conditions, clinical and individual-difference effects, and risk-of-bias concerns. Consequently, the evidence supports with low certainty the conclusion that acute caffeine modifies beta activity, but does not establish a reproducible direction of change.

### 3.7. EEG Frequency Measures

EEG frequency measures, including peak, mean, and dominant frequency, were examined less frequently than spectral power. Among studies of acute caffeine administration under non-sleep-deprived conditions, the available evidence generally suggested a shift toward faster ongoing rhythms, particularly within the alpha and beta ranges. However, this effect was not universal, and several studies found substantial power changes without corresponding changes in peak or dominant frequency. Findings derived from caffeine withdrawal or reintroduction, sleep deprivation, habitual-exposure comparisons, and combined-intervention paradigms are considered separately in the exposure-specific sections.

One of the clearest dose-dependent frequency effects was observed in regular female coffee drinkers who received experimentally administered caffeine at 1.5, 3.0, or 6.0 mg/kg. Caffeine did not significantly alter relative power in the delta, theta, alpha, or beta bands. Instead, the principal electrophysiological changes involved dominant frequency. Dominant alpha frequency increased significantly only after the highest dose of 6.0 mg/kg, whereas dominant beta frequency increased after both 3.0 and 6.0 mg/kg. Thus, acute caffeine shifted the dominant frequencies of alpha and beta activity upward without necessarily increasing their spectral power [50].

A similar pattern was observed in a resting-state arousal study using 250 mg caffeine. Mean alpha frequency increased from 10.16 Hz under placebo to 10.30 Hz after caffeine, and the effect was broadly distributed rather than confined to a specific scalp region. The frequency increase occurred together with a reduction in alpha power, indicating that acute caffeine produced a weaker but slightly faster alpha rhythm [51].

Acute caffeine also accelerated alpha activity in children. Following 80 mg caffeine, mean alpha frequency increased slightly from approximately 9.74 to 9.81 Hz. Unlike the relatively global adult effect reported in [51], the frequency increase in children was more pronounced posteriorly than frontally [56]. This finding suggests that alpha-frequency acceleration can also occur in younger participants, although its topographic expression may differ developmentally.

In participants with panic disorder and healthy controls, caffeine also increased peak occipital alpha frequency. Relative to placebo, the increase was approximately 0.32 Hz at 30 min and 0.40 Hz at 75 min after administration. The frequency response did not differ substantially between the panic-disorder and healthy-control groups, suggesting that the acute acceleration of peak alpha frequency was not specific to the clinical diagnosis [55].

Further evidence came from EEG cartography. Acute caffeine increased mean alpha frequency by as much as approximately 0.34 Hz, with increases observed in 11 of 16 EEG channels. This frequency acceleration occurred simultaneously with reductions in alpha RMS amplitude, relative alpha percentage, theta amplitude, and overall raw EEG RMS amplitude. Thus, the caffeine-related EEG profile combined faster alpha oscillations with reduced large-amplitude synchronized activity [66].

However, acute caffeine did not consistently alter frequency parameters. In poor and normal sleepers receiving 2.5 mg/kg caffeine, peak alpha frequency was not significantly changed despite pre-existing differences between the two groups. Poor sleepers showed a faster baseline peak alpha frequency, but acute caffeine did not further accelerate this rhythm [45]. Similarly, in a visual selective-attention experiment, caffeine reduced resting/background EEG power, particularly in the lower alpha range, but did not significantly alter dominant alpha peak frequency [67]. These studies demonstrate that substantial effects on alpha power can occur independently of measurable changes in alpha frequency.

Individual characteristics may also moderate frequency responses. In a coping-style study, caffeine increased dominant alpha frequency and dominant beta frequency, broadly consistent with faster waking EEG activity after acute administration. However, the response was not uniform across participant subgroups, and one subgroup showed an opposite alpha-frequency pattern. These findings suggest that individual psychological or physiological characteristics may contribute to variability in the frequency response to caffeine [71].

Overall, the direct acute-administration evidence suggests that caffeine can shift waking EEG rhythms toward faster mean, peak, or dominant frequencies, particularly within the alpha range. Increased alpha frequency was reported across dose-response, resting-state, clinical, developmental, and EEG-cartography studies [50,51,55,56,66], while increased dominant beta frequency was reported in selected experiments [50,71]. Nevertheless, frequency acceleration was not universal, and some studies demonstrated clear caffeine-related changes in spectral power without significant alterations in peak or dominant frequency [45,67]. Therefore, frequency acceleration should be regarded as a recurring but less consistently measured acute electrophysiological effect of caffeine rather than a universal feature of the caffeine-induced waking EEG profile. Importantly, normalization of slowed frequencies during caffeine withdrawal, tobacco/caffeine deprivation, or sleep-loss paradigms represents a distinct exposure context and should not be interpreted as equivalent evidence for acute frequency acceleration from a non-deprived baseline.

Eight studies provided direct acute, non-sleep-deprived evidence on mean, peak, or dominant EEG frequency. Five reported a predominant acceleration of EEG frequency, principally within the alpha range [50,51,55,56,66], none reported a consistent overall slowing, two reported no significant frequency change [45,67], and one showed mixed or subgroup-dependent findings [71]. Increased beta dominant frequency was additionally observed in [50,71]. The principal contexts were resting-state dose-response experiments, arousal studies, panic-disorder and developmental samples, EEG cartography, and individual-difference paradigms. Four studies had some concerns under RoB 2, two were at high RoB 2 risk, and two non-randomized studies had serious ROBINS-I risk. Thus, although the directional pattern favors frequency acceleration, the small evidence base and substantial risk-of-bias burden support only low certainty for this EEG domain.

Certainty of evidence is low. The direction of the available acute findings is relatively consistent, particularly for alpha-frequency acceleration, but the evidence is derived from substantially fewer studies than spectral-power outcomes and includes small samples, heterogeneous frequency metrics, different doses and participant populations, and risk-of-bias concerns. Confidence is therefore limited in the generalizability of caffeine-related EEG frequency acceleration, although the evidence suggests that increases in mean, peak, or dominant alpha frequency are a reproducible response in selected acute-administration paradigms.

### 3.8. EEG Coherence and Hemispheric Asymmetry

EEG coherence and hemispheric asymmetry were examined much less frequently than conventional spectral-power measures. After separating withdrawal/reintroduction and combined-intervention paradigms from direct acute caffeine administration, the evidence concerning these spatial EEG measures is limited to only a small number of studies. Consequently, conclusions regarding caffeine-related changes in functional synchronization or hemispheric organization should be considered preliminary.

The clearest direct acute finding concerned EEG coherence. In a qEEG study using caffeine sodium benzoate, caffeine increased coherence across much of the cortex, with the most prominent increases occurring in the beta2 and gamma frequency ranges. These coherence changes were accompanied by reduced integrated EEG power in parieto-occipital regions, reductions in delta, theta, and alpha1 power in selected areas, and a comparatively limited increase in beta2 power. Thus, the principal connectivity-related finding was not a generalized increase in spectral amplitude but rather enhanced synchronization of faster-frequency activity across distributed cortical regions [60].

Evidence for hemispheric asymmetry was more limited. In the study of individuals with early-phase schizophrenia and healthy controls, alpha asymmetry was calculated across frontal and parietal left–right electrode pairs. However, the principal caffeine-related findings involved changes in alpha1, alpha2, and beta power rather than a clear alteration in hemispheric asymmetry. The available results therefore do not indicate that acute caffeine produced a robust or central alpha-asymmetry effect in this sample [37].

Overall, direct acute evidence for caffeine-related changes in EEG coherence and hemispheric asymmetry is very limited. One study demonstrated increased beta2 and gamma coherence across a broad cortical distribution after acute caffeine administration [60], whereas the available asymmetry analysis did not identify a clear caffeine-sensitive left–right effect [37]. These findings suggest that acute caffeine may influence large-scale synchronization of faster EEG activity, but there is currently insufficient evidence to determine whether this represents a reproducible feature of the waking caffeine response. Evidence for withdrawal-related coherence changes and nicotine-dependent frontal asymmetry should be interpreted separately within their respective exposure categories and should not be used to strengthen conclusions regarding direct acute caffeine administration.

Direct acute evidence for EEG coherence and hemispheric asymmetry was available from only two studies. One study reported increased beta2 and gamma coherence after caffeine [60], whereas one study found no clear caffeine-related hemispheric asymmetry effect [37]; no direct acute study demonstrated a predominant decrease or a mixed directional effect within this restricted evidence set. The principal contexts were resting-state qEEG coherence and frontal/parietal asymmetry analysis. Of the two contributing studies, one had some concerns under RoB 2 and the other had serious ROBINS-I risk. Two additional studies reported condition-dependent spatial effects in distinct exposure contexts—withdrawal-related coherence changes [40] and nicotine-dependent frontal asymmetry [61]—and these were therefore not included in the direct acute counts. The very small number of independent studies and the risk-of-bias profile support a very-low-certainty conclusion.

Certainty of evidence is very low. Confidence is constrained primarily by the very small number of contributing studies, small samples, methodological heterogeneity, different connectivity and asymmetry metrics, and risk-of-bias concerns. Accordingly, the available evidence is insufficient to establish a consistent acute caffeine effect on EEG coherence or hemispheric asymmetry.

### 3.9. Total EEG Power and Global EEG Amplitude

Total EEG power, broad-band spectral power, and global EEG amplitude were examined less frequently than individual frequency bands. Among studies of acute caffeine administration under non-sleep-deprived conditions, the predominant finding was a reduction in the overall magnitude of spontaneous waking EEG activity. This pattern was generally interpreted as EEG desynchronization or cortical activation, because caffeine tended to reduce large-amplitude synchronized activity rather than produce a generalized increase in total electrophysiological power. However, the effect was not universal and varied according to eye condition, behavioral state, dose, scalp region, and the particular global or broad-band metric used. Studies involving sleep deprivation, caffeine withdrawal or chronic exposure, and caffeine combined with other interventions are considered separately in the exposure-specific sections.

In an early topographic qEEG study using 200 mg caffeine, total EEG power decreased significantly during eyes-open rest across a broad cortical distribution, including frontal, central, temporal, parietal, and occipital regions. During eyes-closed recordings, EEG power showed a similar tendency to decrease at multiple sites, but these effects did not reach statistical significance. Thus, the reduction in total EEG power was most evident during eyes-open wakefulness, indicating that the global caffeine effect depended partly on recording state [31].

A similar broad reduction was observed in a study comparing acute doses of 200 and 400 mg caffeine. During relaxed eyes-open rest, both doses produced widespread decreases in spectral power, although the topographic and frequency-specific distributions differed according to dose. The effect was therefore not characterized simply by an increase in faster EEG activity; rather, caffeine reduced overall power density during relaxed wakefulness. During concentration-task performance, however, the pattern changed and some of the broad resting-state reductions were no longer evident, demonstrating that behavioral engagement substantially modified the global EEG response [32].

In healthy volunteers receiving 400 mg caffeine, overall log absolute EEG power was also significantly lower after caffeine than after placebo. Although the reduction was particularly prominent in the alpha range, the analysis demonstrated a broader decrease in absolute EEG power. This finding was interpreted as consistent with central nervous system stimulation because the waking EEG became less dominated by high-amplitude synchronized resting activity [38]. Another acute psychopharmacological study using 250 and 500 mg caffeine similarly indicated a reduction in broad EEG power, largely reflecting decreases in slower-frequency and alpha activity. The authors proposed that caffeine could reduce power across several bands while simultaneously producing more complex effects within faster frequencies [48].

Caffeine also reduced spontaneous and visually driven EEG activity in a photic-stimulation experiment. Acute caffeine decreased resting EEG power at 10 Hz and 15 Hz and attenuated photic-driving responses at several stimulation frequencies. Thus, the electrophysiological effect was characterized by reduced spontaneous and stimulus-driven spectral magnitude rather than generalized enhancement of cortical responsiveness [49].

Broad-band amplitude reductions were also reported in dose-comparison studies. Both 250 and 500 mg caffeine decreased EEG amplitude across the 4–30 Hz range, particularly over central and parietal regions, with corresponding reductions in alpha and beta activity. However, the 500 mg dose did not produce proportionally larger changes than the 250 mg dose. Consequently, the decrease in broad-band EEG amplitude did not follow a simple monotonic dose-response relationship [74].

EEG cartography provided convergent evidence. Acute caffeine reduced overall raw EEG RMS amplitude by as much as approximately 22 µV in 6 of 16 channels. This occurred together with decreased alpha RMS amplitude, decreased theta amplitude, and increased mean alpha frequency. The combination of reduced overall amplitude and faster alpha activity was interpreted as a desynchronized waking EEG profile, in which spontaneous activity became less dominated by large-amplitude synchronized oscillations [66].

A qEEG study using caffeine sodium benzoate also demonstrated reductions in integrated EEG power, although the effect was spatially selective rather than fully global. Integrated power decreased particularly in parieto-occipital regions, together with reductions in several slower-frequency components. At the same time, caffeine increased beta2 and gamma coherence and produced a limited beta2 power increase. Thus, acute caffeine could reduce broad posterior EEG magnitude while simultaneously increasing synchronization within faster-frequency networks [60].

Clinical studies showed broadly compatible but less uniform patterns. In participants with panic disorder and healthy controls, caffeine reduced several amplitude measures, including slow-alpha, theta, and central beta activity, while central delta amplitude remained unchanged. The overall pattern therefore reflected a reduction in multiple EEG amplitudes together with an increase in posterior alpha frequency rather than a generalized increase in signal magnitude [55].

Task demands could also determine whether a broad caffeine effect was detectable. In a study comparing caffeine with alcohol and diphenhydramine, 200 mg caffeine did not significantly alter resting EEG relative to placebo. During task performance, however, caffeine significantly reduced parietal alpha power. This indicates that some acute caffeine effects may emerge as frequency- and task-specific changes without producing a detectable alteration in global resting EEG magnitude [75]. Similarly, in a visual selective-attention study, caffeine reduced resting/background EEG power across several frequency ranges before task onset, with the largest decrease occurring in lower alpha and smaller reductions in higher alpha and delta activity. This represented a broad but frequency-selective reduction in spontaneous activity rather than uniform suppression across the entire EEG spectrum [67].

Overall, the direct acute-administration evidence suggests that caffeine commonly reduces total EEG power, broad-band spectral power, or global EEG amplitude during ordinary wakefulness, particularly during relaxed and eyes-open resting conditions [31,32,38,48,49,66,74]. Rather than producing a generalized increase in electrophysiological activity, acute caffeine more often reduced the magnitude of large-amplitude synchronized EEG activity, consistent with a shift toward a more desynchronized waking pattern. However, this response was not universal: some experiments showed predominantly regional or frequency-selective effects [55,60,67], while others found no significant resting global effect despite task-related changes [75]. Therefore, reduced total or broad-band EEG magnitude appears to be a recurrent but state- and methodology-dependent feature of the acute caffeine response rather than a universal electrophysiological signature.

Eleven studies contributed direct acute evidence concerning total EEG power, broad-band spectral magnitude, or global EEG amplitude. Seven reported a predominant decrease [31,32,38,48,49,66,74], none reported an overall increase, one found no significant global resting-state effect [75], and three produced regional, frequency-selective, or otherwise condition-dependent findings rather than a uniform global response [55,60,67]. The predominant context was relaxed resting-state EEG, particularly eyes-open recordings, together with broad-band amplitude, EEG-cartography, and photic-stimulation paradigms. Seven contributing studies had some concerns under RoB 2, two had high RoB 2 risk, and two non-randomized studies had serious ROBINS-I risk. The numerical predominance of decreases supports a recurring caffeine-related reduction in broad EEG magnitude, but methodological heterogeneity and the absence of a universal response justify the current low-certainty rating.

Certainty of evidence is low. Although decreases in total or broad-band EEG magnitude were replicated across several acute-administration studies, confidence is limited by generally small samples, heterogeneous definitions of total power and global amplitude, differences in dose, eye condition, recording state and EEG methodology, and risk-of-bias concerns. The available evidence therefore supports with low certainty the conclusion that acute caffeine tends to reduce broad waking EEG magnitude, particularly during relaxed resting conditions.

### 3.10. Acute Caffeine Administration Under Rested or Non-Sleep-Deprived Conditions

Most studies examined the effects of a single acute caffeine dose under ordinary wakeful conditions without experimentally induced sleep deprivation or caffeine withdrawal. Across these studies, the most reproducible finding was a reduction in alpha power or amplitude, although the magnitude and topography of this effect varied across eyes-open, eyes-closed, and task conditions [31,34,36,37,38,48,51,52,55,56,66,67,74,75,78]. Several studies additionally reported increased mean, peak, or dominant alpha frequency [50,51,55,56,66,71]. These findings support an acute pharmacological effect of caffeine characterized primarily by attenuation of synchronized alpha activity and, in some studies, acceleration of the dominant waking rhythm.

Effects on slower and faster bands were considerably less uniform. Acute caffeine reduced theta in several studies [32,48,55,56,62], whereas other rested-state experiments reported weak or absent theta changes [31,33,34,35,45,49,50,51,52]. Delta activity was similarly inconsistent: reductions occurred in some acute paradigms [32,33,46,56], whereas other studies showed minimal, focal, or no significant changes [31,34,35,37,45,55]. Beta responses were particularly heterogeneous, with decreases [31,37,52,55,56,62,74,78], increases in selected experiments [46,57], and null or weak effects in others [33,34,35,45,47,50,51]. Thus, the direct acute evidence supports alpha suppression more consistently than a generalized decrease in delta/theta or increase in beta activity.

Less frequently examined acute EEG outcomes also suggested that caffeine can modify the organization rather than merely the magnitude of waking activity. Caffeine increased beta2/gamma coherence while reducing selected lower-frequency power in one qEEG study [60], produced a nonlinear dose- and task-dependent change in EEG complexity in one study [65], and altered resting EEG microstate dynamics in individuals with early-phase psychosis [68]. In the only study directly assessing frontal theta/beta ratio, 200 mg caffeine reduced theta and beta power separately but did not significantly change their ratio [62]. These less frequently studied effects should be regarded as preliminary because they were generally supported by single studies.

The conclusion that acute caffeine commonly reduces alpha power or amplitude, and may accelerate alpha frequency, is supported primarily by direct acute-administration studies conducted without experimentally induced sleep deprivation or caffeine withdrawal. By contrast, direct acute evidence for consistent delta, theta, or beta changes is weaker and more heterogeneous.

### 3.11. Caffeine During Sleep Deprivation, Sleep Restriction, or Prolonged Wakefulness

A separate group of studies administered caffeine in the context of experimentally increased sleep pressure or prolonged wakefulness [41,44,54,58,63,64,76]. These findings differed from ordinary acute-administration studies because caffeine was evaluated against an electrophysiological background characterized by progressive drowsiness and increasing slow-frequency activity.

The clearest finding was attenuation of sleep-loss-related theta activity. During 40 h of sustained wakefulness, two 200 mg doses of caffeine reduced waking theta power, with the effect becoming particularly evident after prolonged time awake [41]. After acute sleep deprivation, 400 mg caffeine reduced relative theta together with alpha activity [54]. Following more prolonged sleep deprivation, 600 mg slow-release caffeine reduced absolute and relative delta and theta power while increasing beta activity [58]. Caffeine also reduced sleep-deprivation-related theta/alpha activity in caffeine-sensitive and caffeine-insensitive men, with a particularly pronounced frontocentral theta effect in caffeine-sensitive participants [76].

Driving and sustained-vigilance paradigms provided related but more conditional evidence. During early-morning driving, 200 mg caffeine reduced the progressive increase in 4–11 Hz theta–alpha activity after partial sleep restriction, whereas this effect was substantially weaker after total sleep deprivation [63]. In another total sleep-deprivation experiment, acute caffeine attenuated sleep-loss-related EEG power increases across several regions during sustained attention [64]. The dietary-caffeine study including rested and sleep-restricted conditions produced only scattered delta and beta changes and no robust alpha or theta effect [44].

Evidence that caffeine attenuates theta and, under more severe sleep loss, delta activity is supported most strongly by sleep-deprivation and prolonged-wakefulness studies rather than by rested acute-administration experiments. These findings should therefore be interpreted as evidence that caffeine counteracts electrophysiological manifestations of accumulating sleep pressure, not as evidence that caffeine universally suppresses slow-frequency activity during normal rested wakefulness.

### 3.12. Caffeine Withdrawal and Reintroduction

Withdrawal and reintroduction studies produced an electrophysiological pattern that should be distinguished from direct acute caffeine effects [40,42,59]. In habitual caffeine users undergoing several days of abstinence, withdrawal increased absolute theta power broadly, particularly over frontal regions, and increased frontal absolute delta power more focally [40]. Withdrawal also decreased relative beta power and slowed mean alpha and beta frequencies. Frontal alpha and theta coherence increased, whereas posterior theta and delta coherence decreased. When caffeine was reintroduced, several of these abnormalities, including increased theta power and slowed alpha and beta frequency, returned toward baseline [40].

A second controlled study comparing chronic caffeine maintenance, acute caffeine, and abstinence similarly found that withdrawal increased theta power and decreased beta2 power, while an acute caffeine challenge increased beta2 activity [42]. Importantly, this design indicated that chronic caffeine administration produced relatively few persistent beneficial neurophysiological effects. A further dietary-caffeine study specifically designed to distinguish acute caffeine effects from withdrawal and withdrawal reversal found relatively modest EEG changes overall; alpha activity increased during withdrawal, while direct caffeine effects on several conventional spectral measures were limited [59].

Increased theta activity, frontal delta activity, slower alpha/beta frequencies, and altered coherence are supported primarily as markers of caffeine withdrawal. Their normalization after caffeine reintroduction represents withdrawal reversal and should not be interpreted as equivalent to enhancement produced by caffeine from a physiologically neutral, non-withdrawn baseline.

### 3.13. Chronic and Habitual Caffeine Exposure

Evidence concerning chronic or habitual caffeine exposure was more limited and should be distinguished from acute caffeine administration. In the controlled maintenance study, chronic administration of approximately 400 mg/day produced few persistent EEG benefits, with the clearest chronic effect involving beta2 activity [42]. The dietary-caffeine study similarly found relatively modest and inconsistent chronic effects across delta, theta, alpha, and beta activity [44], while the controlled chronic/abstinence protocol [59] provided little evidence for a large stable EEG activation effect attributable to sustained caffeine consumption.

Observational or subgroup findings suggested that habitual intake may instead modify baseline EEG characteristics and acute responsiveness. In healthy controls, greater habitual caffeine intake was associated with lower resting delta power, whereas this relationship was not apparent in the schizophrenia group [37]. In participants divided according to low and high habitual caffeine intake, baseline EEG characteristics and the response to caffeinated coffee differed substantially between consumption groups [43]. Low habitual consumers showed clearer reductions in delta and theta activity and increases in beta2 after caffeinated coffee, whereas high habitual consumers showed more prominent alpha reductions and more heterogeneous delta responses [43]. During total sleep deprivation, habitual caffeine consumption did not consistently modify acute caffeine-induced power changes, but high habitual consumers showed lower individual alpha frequency than low or moderate consumers [64].

The chronic/habitual evidence does not demonstrate a stable EEG equivalent of the acute stimulant response. Instead, habitual intake appears principally to influence baseline EEG characteristics, withdrawal vulnerability, and the magnitude or direction of subsequent acute caffeine responses. These associations should therefore be interpreted as chronic-exposure or effect-modification findings rather than direct acute pharmacological effects.

### 3.14. Coffee-Based Interventions

Coffee-based interventions were considered separately because coffee contains caffeine within a complex beverage matrix and should not automatically be treated as pharmacologically equivalent to administration of isolated caffeine. Three studies in the present synthesis used coffee-based exposure [39,43,53].

In the wearable-EEG pilot study, consumption of coffee containing approximately 162 mg caffeine was associated with reduced alpha activity and increased beta activity, particularly over central and right sensorimotor regions [39]. In the study comparing caffeinated with decaffeinated coffee in low and high habitual caffeine consumers, responses depended strongly on prior habitual intake [43]. Low habitual consumers showed lower eyes-closed delta and theta activity and greater beta2 activity after caffeinated coffee, whereas high habitual consumers showed reductions in alpha1 and alpha2 and a more heterogeneous delta response. Thus, the coffee effect was not uniform across habitual-intake groups [43].

In another resting-state coffee study using approximately 181 mg caffeine, coffee appeared to oppose the spontaneous drift toward a lower-vigilance EEG state over time. Slow theta activity remained lower in selected regions, beta relative power was higher in some centro-parietal regions, and the evolution of alpha activity differed from the control condition [53]. This pattern was interpreted primarily as maintenance of wakeful arousal rather than a uniform suppression of all alpha activity.

Coffee-based studies support a shift toward greater vigilance, often involving reduced slow or alpha activity and/or increased beta activity, but the limited number of studies and strong moderation by habitual caffeine consumption prevent these findings from being treated as interchangeable with evidence from isolated caffeine administration.

### 3.15. Caffeine Combined with Another Intervention

Several studies examined caffeine in conjunction with another pharmacological, behavioral, or environmental manipulation, and these findings should be interpreted separately because the observed EEG changes cannot always be attributed to caffeine alone [57,61,69,70,72,77].

In studies involving smoking or nicotine, interactions were complex. In regular smokers and coffee drinkers, acute caffeine increased beta and reduced theta activity, while several other spectral measures remained unchanged; the major caffeine effects were not uniformly modified by smoking [57]. In a factorial nicotine–caffeine experiment, caffeine broadly reduced power across multiple frequency bands, but its effects depended on electrode location, eye condition, time, and nicotine exposure. Caffeine and nicotine also interacted in measures of frontal theta and alpha asymmetry [61]. In nicotine- and caffeine-deprived smokers, caffeine suppressed deprivation-related frontal theta activity and, at 300 mg, prevented persistent slowing of posterior alpha frequency [70]. These findings primarily demonstrate caffeine effects in the context of nicotine exposure or tobacco withdrawal rather than an uncomplicated acute caffeine response.

Two studies investigated caffeine together with L-theanine [69,77]. In one attention experiment, 50 mg caffeine alone did not significantly reduce tonic alpha amplitude, whereas the caffeine-plus-L-theanine condition significantly reduced tonic alpha activity relative to placebo [69]. In a later sustained-attention study, caffeine reduced tonic alpha activity by approximately 12% early in the task, with the EEG effect diminishing over time despite longer-lasting behavioral effects; the experimental design additionally included theanine conditions [77]. Consequently, effects attributable specifically to the combination should be separated from effects of caffeine alone.

Bright light provided another example of interaction with a non-pharmacological intervention. In chronically mildly sleep-restricted drivers, caffeine alone did not reliably reduce theta or alter alpha activity, whereas bright light produced clearer EEG effects. The combined bright-light-plus-caffeine condition reduced alpha power more strongly than either intervention alone, while the theta response did not provide evidence for a straightforward additive caffeine effect [72].

Evidence from combined-intervention studies is heterogeneous and intervention-specific. L-theanine, nicotine or smoking state, tobacco withdrawal, and bright light can modify the electrophysiological response observed in the presence of caffeine. These studies therefore support interaction-specific conclusions and should not be used as equivalent evidence for the direct effect of caffeine alone.

### 3.16. Nonlinear EEG Complexity

Nonlinear EEG complexity was examined much less frequently than spectral power, frequency, or topographic measures, but it provided a distinct view of caffeine’s influence on wakefulness EEG. Instead of asking whether caffeine increased or decreased activity in a specific frequency band, this parameter examined whether caffeine changed the temporal structure and dynamic complexity of the EEG signal. The available evidence suggests that caffeine may alter EEG complexity in a nonlinear, dose-dependent, and task-dependent manner rather than producing a simple uniform increase or decrease in cortical activity. One study assessed EEG complexity using the correlation dimension, or D2, across repeated cognitive testing sessions and caffeine doses. The main finding was that caffeine did not change EEG complexity in a simple monotonic direction. In the placebo condition, changes in D2 across sessions were best described by a linear trend, suggesting that repeated testing or time-on-task produced relatively regular changes in EEG complexity. Under caffeine, however, linear models explained almost none of the variance in D2, whereas quadratic models explained substantially more variance. This means that caffeine changed the relationship between testing session, dose, task condition, and EEG complexity: the response was curvilinear rather than linear [65]. The direction of this nonlinear effect depended on the cognitive task. For three of the four cognitive tasks, the quadratic relationship was positive, suggesting a U-shaped pattern in which EEG complexity shifted at intermediate caffeine doses and then changed again at higher doses. For one difficult alphabetical task, the quadratic coefficient was negative, indicating an inverted pattern. Therefore, caffeine did not simply increase EEG complexity as arousal rose, nor did it simply reduce complexity through global desynchronization. Instead, the complexity response varied with task demands and caffeine dose [65].

This finding is important because it shows that caffeine’s wakefulness EEG effects cannot be fully captured by conventional spectral power measures. In several power-based studies, caffeine was described as reducing alpha, theta, delta, total EEG amplitude, or increasing beta under some conditions. However, the nonlinear complexity result suggests that caffeine can also reorganize the temporal dynamics of cortical activity in a more complex way. The EEG signal may become more or less complex depending on the interaction between pharmacological stimulation, task difficulty, and dose level [65].

Overall, nonlinear EEG complexity represents a separate and less commonly studied parameter of caffeine’s effects on wakefulness EEG. The available evidence indicates that caffeine modifies EEG complexity through a curvilinear dose-response pattern rather than a simple directional change. This suggests that caffeine’s effect on cortical dynamics may be optimal or most distinct at particular dose levels and under particular task demands, while higher or lower doses may produce different patterns. Because this parameter was reported in only one summarized study, the evidence remains preliminary, but it indicates that caffeine may influence not only EEG power and frequency, but also the nonlinear organization of ongoing brain activity [65].

Certainty of evidence is very low. Evidence for caffeine-related changes in nonlinear EEG complexity was derived from a single small study [65], which also contained an important risk-of-bias concern. Consequently, the reported nonlinear and curvilinear dose-response relationship should be considered exploratory and hypothesis-generating. Independent replication using contemporary nonlinear EEG methods is required before a reliable effect of caffeine on EEG complexity can be established.

### 3.17. EEG Microstates

Resting-state EEG microstates were examined in one study of acute caffeine effects in early-phase schizophrenia. This parameter differed from conventional spectral EEG measures because it did not quantify power in delta, theta, alpha, or beta bands. Instead, it assessed the temporal characteristics of recurring scalp-map configurations, including their duration, occurrence, and total contribution to the resting EEG signal. Therefore, microstate analysis provided information about the large-scale temporal organization of brain activity during wakefulness. The study compared 12 individuals with early-phase schizophrenia and 13 healthy controls. Each participant completed two double-blind sessions: one after 200 mg caffeine and one after placebo. Resting EEG was recorded approximately 30 min after administration during eyes-open and eyes-closed conditions. The analysis identified the standard microstate classes A, B, C, and D and compared their temporal parameters between groups and drug conditions [68]. The main baseline finding was that individuals with early-phase schizophrenia showed an abnormal microstate profile compared with healthy controls. The clearest abnormality concerned microstate class D. In the schizophrenia group, class D had shorter duration, fewer occurrences per second, and lower total contribution than in healthy controls. This is relevant because reduced class D has often been discussed as a microstate marker of schizophrenia. In this study, caffeine intensified this class D abnormality. In patients only, caffeine reduced class D duration, reduced class D occurrence, and reduced the proportion of total recording time spent in class D. For example, class D contribution decreased from about 37.6% after placebo to about 17.3% after caffeine in the schizophrenia group. This caffeine-related reduction in class D was not observed in healthy controls [68]. Caffeine also affected other microstate classes, again most clearly in the schizophrenia group. For microstate class A, patients showed longer duration and greater contribution than controls, and caffeine increased class A occurrence in the patient group only. For microstate class B, patients spent more total time in this state than controls, and caffeine increased class B occurrence across the full sample. However, the strongest drug-by-group effect again appeared in the schizophrenia group, where class B occurrence increased from about 3.34 occurrences per second after placebo to about 5.47 occurrences per second after caffeine. There were also trend-level effects suggesting that caffeine increased the total contribution of classes A and B in patients [68]. Microstate class C showed a different pattern. Patients had fewer class C occurrences than healthy controls, but caffeine did not significantly affect this class. The authors also examined whether clinical symptoms were related to microstate parameters. During placebo, class C contribution was negatively correlated with PANSS negative symptoms, meaning that greater negative symptom severity was associated with less time spent in class C. However, caffeine-induced changes in microstate duration, occurrence, or contribution were not significantly related to symptom severity measured by PSYRATS, PANSS, or BNSS. Thus, the caffeine-related microstate effects did not appear to depend directly on measured positive or negative symptom severity in this small sample [68].

Overall, the microstate findings suggest that caffeine can alter the temporal organization of resting EEG, but the available evidence is limited to one early-phase schizophrenia study. The most important result was that caffeine worsened or amplified the already abnormal microstate pattern in the schizophrenia group, especially by reducing class D duration, occurrence, and contribution. At the same time, caffeine increased the occurrence or contribution of classes A and B, mainly in patients, while class C was not significantly affected by caffeine [68]. Therefore, microstate analysis suggests that caffeine’s effects on wakefulness EEG may involve changes in large-scale brain-state dynamics, not only changes in spectral power or frequency. However, because this parameter was reported in only one summarized study and mainly in a clinical sample, it should be interpreted cautiously and not generalized to all healthy wakefulness EEG studies.

Certainty of evidence is very low. The microstate evidence was derived from only one study [68], conducted in a small sample that included individuals with early-phase schizophrenia. Although the study used a controlled caffeine/placebo design, risk-of-bias concerns remained and the clinical population limits direct generalization to healthy individuals. The observed effects on microstate classes A, B, and particularly D should therefore be considered preliminary and cannot yet be regarded as an established electrophysiological effect of caffeine.

### 3.18. Theta/Beta Ratio

The theta/beta ratio was examined directly in only one included study, making it a much less frequently reported EEG parameter than absolute or relative power in individual frequency bands. The study that assessed this parameter investigated whether acute caffeine intake modifies attention toward threatening information and whether this behavioral effect is related to trait anxiety and frontal EEG theta/beta ratio. The participants were healthy young women who were selected as low habitual caffeine users, and the experimental manipulation consisted of 200 mg caffeine compared with placebo [62].

The main EEG finding was that caffeine did not significantly change the frontal theta/beta ratio itself. Therefore, the study did not support the hypothesis that caffeine would modify TBR and that this TBR change would explain caffeine-related behavioral effects. This is important because TBR is often interpreted as a composite marker of the balance between slower theta activity and faster beta activity, but in this study the ratio was not sensitive to acute caffeine administration [62].

However, the absence of a significant TBR effect did not mean that caffeine had no effect on the underlying frequency bands. When theta and beta were analyzed separately, caffeine significantly reduced both frontal theta power and frontal beta power compared with placebo. This pattern suggests that caffeine changed frontal spectral power, but not in a way that shifted the balance between theta and beta strongly enough to alter their ratio. In other words, because both the numerator of the ratio, theta power, and the denominator, beta power, decreased after caffeine, the composite theta/beta ratio remained statistically unchanged [62].

These findings distinguish TBR from separate theta and beta measures. Across other studies, caffeine often reduced theta activity under low-vigilance, sleep-pressure, or withdrawal-related conditions, and beta activity sometimes increased, decreased, or shifted in frequency depending on the experimental context. However, most of these studies did not calculate theta/beta ratio directly. Therefore, they cannot be used as direct evidence that caffeine increases or decreases TBR. The only direct TBR evidence indicates no significant acute caffeine effect on the ratio, despite significant reductions in both frontal theta and frontal beta power [62].

Overall, theta/beta ratio appears to be a weakly supported and non-sensitive EEG marker of acute caffeine effects during wakefulness in the available evidence. The single direct study found no significant caffeine-related change in frontal TBR, even though caffeine reduced theta and beta power separately. Therefore, based on the summarized studies, caffeine should not be described as reliably altering theta/beta ratio. Instead, TBR should be treated as a secondary and currently underexplored parameter, with insufficient evidence for a consistent caffeine-related effect [62].

Certainty of evidence is very low. Only one included study directly evaluated the theta/beta ratio [62]. Although this study found no significant caffeine-related change in frontal TBR despite reductions in theta and beta power separately, the absence of independent replication and the restricted participant population substantially limit confidence in this finding. The current evidence is therefore insufficient to conclude either that caffeine meaningfully alters TBR or that TBR is definitively insensitive to caffeine.

### 3.19. EEG–Behavioral Concordance

Across studies simultaneously assessing EEG and behavioral outcomes, electrophysiological evidence of increased arousal was only partially concordant with improvements in objective performance. Importantly, the great majority of studies did not statistically correlate caffeine-induced EEG changes with caffeine-induced behavioral changes. Accordingly, the evidence primarily concerns within-study co-occurrence of neurophysiological and behavioral effects rather than demonstrated participant-level EEG–behavior relationships.

Several studies nevertheless showed clear directional concordance. Suppression of alpha power following only 50 mg caffeine was accompanied by improvements in Trail Making Test performance and forward digit span [34]. Reductions in parietal theta together with increases in beta activity were accompanied by faster numerical Stroop responses and reduced Stroop interference [57]. During prolonged sleep deprivation, marked reductions in relative delta and theta and increases in alpha and beta activity occurred alongside faster choice-reaction and Stroop responses, more correct continuous-performance responses, and reduced subjective sedation [58]. Similarly, substantial caffeine-induced reductions in delta, theta, alpha1, and beta1 power were accompanied by increased target detection during a visual vigilance task [61]. In a selective-attention paradigm, broadly reduced background EEG power, particularly in the lower-alpha range, co-occurred with a 21.7-ms reduction in reaction time and improved target detection [67]. During sustained-attention testing, decreased alpha activity was accompanied by fewer omission and commission errors and faster responses [77].

Concordance was particularly apparent in paradigms involving sleep deprivation, withdrawal, and subjective vigilance. During 40 h of wakefulness, caffeine reduced waking theta by approximately 23–32% and simultaneously reduced subjective sleepiness [41]. Conversely, caffeine withdrawal increased theta, reduced beta activity and slowed dominant frequencies [40,42], while participants reported greater tiredness, fatigue, sluggishness, weariness, drowsiness, and impaired concentration. Reintroduction of caffeine shifted the abnormal EEG measures back toward baseline [40]. Caffeine-sensitive individuals also exhibited more persistent beta enhancement and delta suppression after caffeine together with nocturnal insomnia and prolonged sleep latency [46]. During simulated driving after restricted sleep, reduced theta-plus-alpha activity was observed during part of the caffeine condition together with fewer sleep-related driving incidents and lower subjective sleepiness [63]. These observations provide particularly coherent physiological and phenomenological evidence that reductions in slow EEG activity accompany caffeine-related maintenance of wakefulness.

However, several studies showed clear EEG–behavioral dissociation. In Taekwondo athletes, caffeine decreased frontal delta activity and increased P300 amplitude after fatigue without significantly improving oddball reaction time, accuracy, errors, or agility [33]. A 400-mg dose markedly reduced total and alpha EEG power without significantly changing reaction time or Stroop performance [38]. Spectral alterations in delta and beta activity were observed without significant improvement in sustained-attention performance [44], and another sustained caffeine/withdrawal study showed modest theta and alpha effects but no significant SART effect [59]. After sleep deprivation, reductions in alpha and theta activity were accompanied only by a numerical, rather than treatment-specific, improvement in reaction time [54]. Caffeine also reduced theta and beta power in the emotional-Stroop study [62] without producing an overall improvement in attentional interference. Similarly, task-related parietal alpha suppression during working memory occurred in the absence of significant changes in reaction time or accuracy [75].

Dose-related findings further indicate that a larger electrophysiological response does not necessarily translate into superior performance. In the KLT study, 400 mg caffeine produced broader EEG suppression than 200 mg, yet behavioral performance tended to be best after 200 mg and was poorer after 400 mg than after placebo [32]. Similarly, increases in dominant alpha and beta frequency became most evident at relatively high caffeine doses in another dose-response study, whereas processing performance improved at 1.5–3.0 mg/kg but not at 6.0 mg/kg [50]. In another study, both 250 and 500 mg reduced EEG amplitude, but behavioral enhancement was greater with 250 mg and diminished at the higher dose [74]. These observations are compatible with a nonlinear relationship between central stimulation and behavioral benefit, consistent with the nonlinear caffeine dose–EEG complexity relationship reported independently [65].

Individual differences may also determine whether EEG changes translate into behavioral effects. In low habitual caffeine consumers, caffeinated coffee produced lower delta and theta and higher beta2 activity together with improved MoCA performance, whereas high habitual consumers showed substantial alpha suppression without significant cognitive improvement [43]. Baseline frontal theta/beta ratio also moderated caffeine-related changes in emotional interference as a function of trait anxiety, despite caffeine itself not significantly altering the theta/beta ratio [62]. Under sleep deprivation, caffeine-sensitive participants showed stronger regional theta suppression and greater improvement in vigilant attention than caffeine-insensitive participants [76]. However, the reported correlation in that study linked caffeine-related behavioral improvement to the magnitude of preceding sleep-deprivation impairment rather than directly to the magnitude of EEG change.

### 3.20. Risk of Bias Assessment

The bias risk assessment is presented in Table 2 (ROB2) and Table 3 (ROBINS-I).

Risk-of-bias judgments varied substantially across the included studies (Table 2 and Table 3). Among randomized studies, the most frequent concerns related to incomplete reporting of sequence generation or allocation concealment, uncertainty regarding blinding, and selective reporting of EEG outcomes. Crossover studies required additional consideration because caffeine exposure in one experimental period could potentially influence subsequent periods through residual pharmacological effects, tolerance, or withdrawal. Studies using clearly described randomized treatment sequences, blinded placebo conditions, adequate washout periods, complete repeated measurements, and paired or repeated-measures statistical analyses were considered methodologically stronger. Conversely, crossover studies in which washout procedures, treatment-order effects, or carryover effects were insufficiently reported received more cautious domain and overall judgments.

An additional caffeine-specific concern was withdrawal during placebo or abstinence conditions. In habitual caffeine consumers, abstinence may itself increase sleepiness and slow-wave EEG activity, meaning that an apparent caffeine effect may partly reflect reversal of withdrawal rather than enhancement relative to a physiologically neutral baseline. Accordingly, studies that standardized pre-session caffeine exposure, documented habitual intake and abstinence duration, assessed withdrawal symptoms, and used appropriate placebo conditions were considered less susceptible to bias.

Among non-randomized studies, confounding represented the principal methodological concern. Important potential confounders included habitual caffeine consumption, sleep duration and sleep deprivation, smoking or nicotine use, concomitant medication, time of day, baseline vigilance, and caffeine-withdrawal symptoms. Studies that did not adequately measure or statistically control these variables were assigned higher risk-of-bias judgments, particularly when caffeine exposure was based on habitual consumption rather than experimentally controlled administration.

The overall risk-of-bias profile indicated important methodological limitations across the evidence base. Among the 41 studies assessed using RoB 2, 31 (75.6%) were judged to have some concerns overall and 10 (24.4%) were judged to be at high overall risk of bias; no randomized study met the criteria for an overall low-risk judgment. High-risk classifications were primarily driven by selective reporting of EEG outcomes, missing outcome data, limitations in the randomization process, and, in some studies, multiple interacting methodological concerns. Among the seven non-randomized studies assessed using ROBINS-I, six (85.7%) were judged to have serious overall risk of bias and one study (14.3%) was judged to have critical overall risk of bias. Confounding was the principal limitation in the non-randomized evidence, particularly because habitual caffeine consumption, preceding abstinence or withdrawal, sleep status, smoking, baseline vigilance, medication, and time of day were not consistently controlled. Overall, 17 of the 48 included studies (35.4%) were classified as having high, serious, or critical overall risk of bias, while the remaining 31 studies (64.6%) retained some concerns. Accordingly, the findings from studies at high, serious, or critical risk were given less interpretative weight than findings from better-controlled randomized placebo studies when determining the consistency and certainty of the evidence.

The risk-of-bias assessment indicates that the certainty of the electrophysiological evidence ranges from moderate to very low depending on the EEG outcome. No outcome was considered to have high-certainty evidence because the randomized studies generally contained at least some risk-of-bias concerns, several contained explicit high-risk domains, and the non-randomized studies were affected by serious or, in one case, critical risk-of-bias concerns. The greatest confidence was assigned to the repeatedly observed reduction in alpha activity and to attenuation of theta activity under conditions of elevated sleep pressure, withdrawal, or reduced vigilance. Certainty was lower for delta activity, beta activity, EEG frequency measures, and global EEG power because of inconsistency and methodological heterogeneity, and very low for coherence/asymmetry, nonlinear complexity, EEG microstates, and theta/beta ratio because these outcomes were supported by only a small number of studies and/or studies with substantial risk-of-bias concerns. Accordingly, studies at high, serious, or critical risk of bias were given less interpretative weight than better-controlled placebo studies when formulating the overall conclusions.

**Figure 2:**
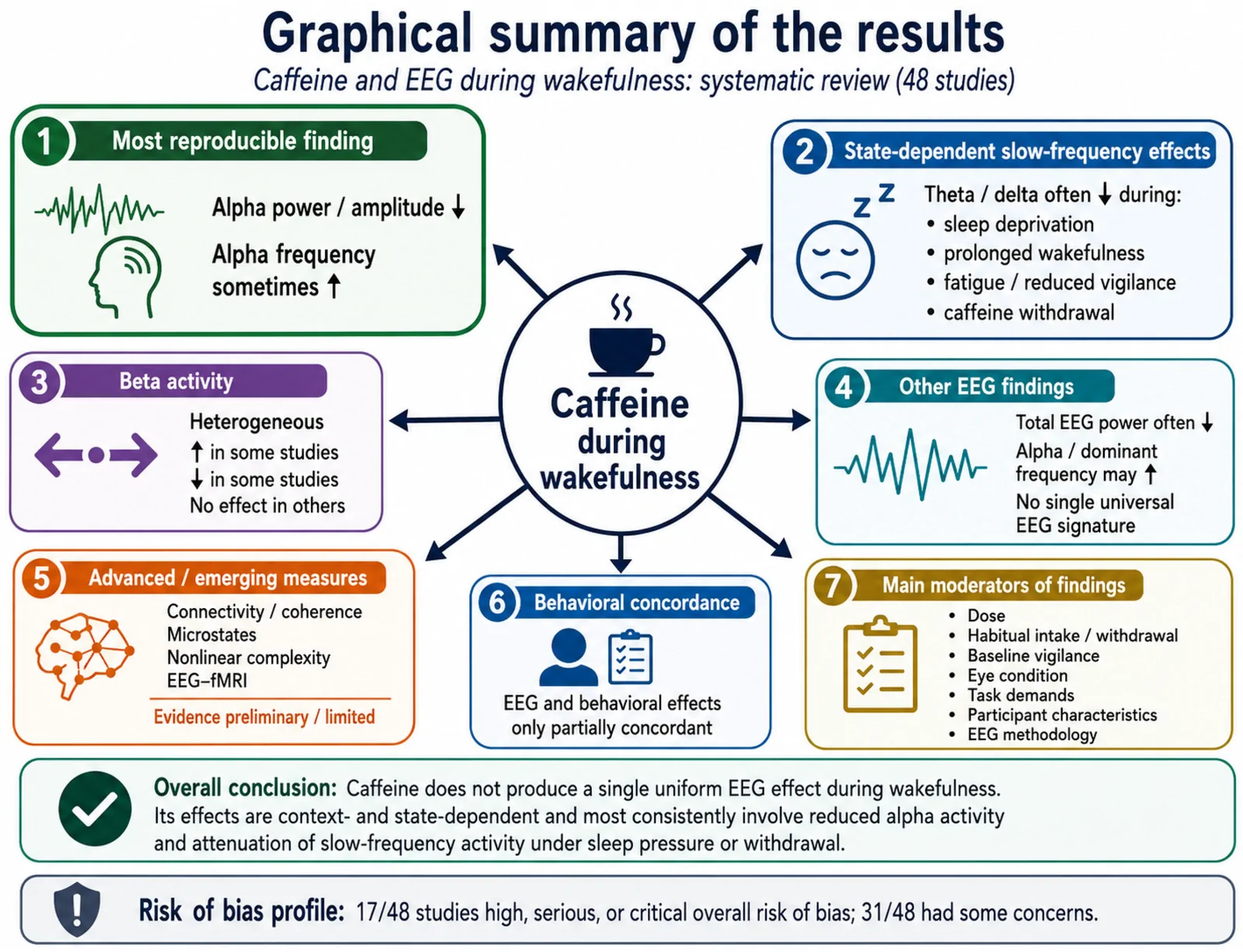
Graphical summary of the results.

Across the 48 included studies, reductions in alpha power or amplitude were among the more frequently reported EEG changes following caffeine administration, whereas reductions in theta, delta, or other slow-frequency activity were reported particularly during sleep deprivation, prolonged wakefulness, caffeine withdrawal, fatigue, or reduced vigilance. Beta-band findings included increases, decreases, and null effects across studies. Changes in spectral frequency, total EEG power, coherence or functional connectivity, asymmetry, nonlinear complexity, EEG microstates, and theta/beta ratio were examined less frequently. EEG changes and behavioral outcomes were not consistently concordant across studies. Of the 48 included studies, 17 (35.4%) were classified as having high, serious, or critical overall risk of bias, while the remaining 31 (64.6%) were classified as having some concerns. Figure 2 provides a graphical summary of the results.

## 4. Discussion

The present review indicates that caffeine does not produce a single, uniform EEG effect during wakefulness. Instead, its electrophysiological profile is best understood as a state-dependent shift in cortical arousal and vigilance. Across the reviewed results, the most reproducible finding was reduced alpha activity, often accompanied by reduced theta or delta activity when participants were sleepy, fatigued, sleep-restricted, undergoing withdrawal, or exposed to monotonous resting conditions. Beta activity was more heterogeneous, sometimes increasing as part of a stimulant-like pattern, but in other studies decreasing together with broader reductions in total EEG power. Additional findings, including increased alpha peak frequency, reduced global spectral power, altered EEG vigilance indices, and partially reversible withdrawal-related slowing, suggest that caffeine mainly reorganizes the waking EEG toward a more activated, less synchronized, and less sleep-prone state rather than simply increasing “fast” activity across all conditions. This interpretation is consistent with the results summarized in the present article, especially the recurring pattern of alpha suppression, theta reduction under low-vigilance conditions, prevention of slow-wave drift, and withdrawal-related EEG slowing.

### 4.1. Comparison with Previous Reviews and Contribution of the Present Review

The present review was conducted as an independently registered systematic review and was not produced by subdividing a single broader systematic-review dataset. Each review in the series had a distinct predefined electrophysiological outcome domain and a separate PROSPERO registration: the ERP review was registered as CRD420261336020, the sleep-related EEG review as CRD420261354163, and the present wakefulness EEG review as CRD420261445522. The reviews also used separate literature searches and eligibility procedures appropriate to their respective outcome domains. The present review therefore extracted only eligible non-ERP EEG outcomes recorded during wakefulness. Conventional ERP amplitude and latency outcomes were reserved for Part 1 [79], whereas EEG and polysomnographic outcomes recorded during sleep were reserved for Part 2 [80]. Where a primary study reported more than one electrophysiological outcome type, only the outcome relevant to the predefined scope of the present review was synthesized here. This outcome-level separation was applied to minimize duplication of evidence, mechanistic interpretation, and conclusions across the three reviews.

Previous reviews of caffeine have primarily focused on behavioral performance, attention, vigilance, sleep, or selected neurophysiological outcomes rather than systematically synthesizing non-ERP EEG recorded during wakefulness. Reviews of cognitive performance have generally concluded that caffeine can improve vigilance, attention, reaction time, and performance, particularly under conditions of fatigue or sleep loss, while also emphasizing that withdrawal reversal may contribute to apparent benefits in habitual users [4,5,7]. The present findings extend this literature by showing that these behavioral effects are not accompanied by a single electrophysiological signature. In particular, reductions in alpha power or amplitude were more consistently observed than generalized increases in fast-frequency activity, whereas EEG changes and behavioral improvement were only partially concordant across studies.

The alpha finding cannot be regarded simply as a replication of an established conclusion from previous reviews because earlier caffeine reviews did not systematically evaluate wakeful non-ERP spectral EEG as a distinct evidence base. Nevertheless, the predominance of alpha suppression is compatible with earlier experimental accounts of caffeine-related increases in arousal and reduced cortical synchronization. The present review strengthens this interpretation by showing that alpha suppression occurred across a range of doses and paradigms, although it remained dependent on eye condition, baseline vigilance, task demands, habitual caffeine use, and scalp region. By contrast, the evidence does not support an equally general reduction in delta or theta activity during ordinary rested wakefulness or a universal increase in beta activity.

An important distinction also emerges when the present findings are compared with the sleep literature. Previous systematic evidence indicates that caffeine impairs subsequent sleep continuity and reduces deep sleep [11], while Part 2 of The Caffeinated Brain found that the most reproducible sleep-EEG effect was attenuation of low-frequency NREM activity, particularly slow-wave and low-delta activity, accompanied in several studies by increases in faster sigma or beta activity [80]. The present wakefulness review identifies a related but not identical pattern. During rested wakefulness, alpha suppression was more reproducible than suppression of slow-frequency activity, whereas theta and delta reductions became more evident during prolonged wakefulness, sleep deprivation, withdrawal, fatigue, or declining vigilance. Thus, sleep and wake EEG findings converge on attenuation of electrophysiological expressions of sleep pressure but differ in the frequency ranges and physiological states in which this effect is most consistently expressed.

Previous experimental work had already identified increasing waking theta activity as a correlate of accumulating sleep pressure and caffeine abstinence, with caffeine re-administration capable of reversing or attenuating these changes [40,41,70]. The present synthesis therefore does not introduce theta suppression as a new general marker of caffeine exposure; rather, it clarifies that theta is most informative when interpreted as a state-dependent marker of sleep pressure, drowsiness, or withdrawal. This distinction helps reconcile studies showing robust theta attenuation during prolonged wakefulness with the numerous null or task-dependent theta findings obtained during rested wakefulness.

The present review also modifies a simple interpretation of beta activity as an electrophysiological marker of stimulant action. Although increased beta or other faster-frequency activity has been reported during sleep and in selected wakefulness paradigms [58,80], the wakeful evidence included beta increases, decreases, and null findings. Beta enhancement therefore cannot be considered a general EEG signature of caffeine during wakefulness. Its direction appears to depend on vigilance state, beta sub-band, scalp region, habitual caffeine exposure, task demands, and the accompanying redistribution of spectral power. This constitutes an important refinement of models in which caffeine-related cortical activation is assumed to produce a uniform shift toward faster EEG activity.

Finally, the inclusion of coherence and functional-connectivity measures, EEG microstates, nonlinear complexity, and multimodal EEG–fMRI findings broadens the scope beyond both conventional spectral EEG and the outcome domains addressed in Parts 1 and 2 [79,80]. These emerging measures suggest that caffeine may influence not only the magnitude and frequency of oscillatory activity but also the spatial coordination and temporal organization of waking brain activity. However, because connectivity, microstate, and complexity findings were supported by only a small number of heterogeneous studies, their principal contribution at present is to identify potential network-level effects and directions for future research rather than to establish additional reproducible caffeine biomarkers.

### 4.2. Adenosine Antagonism as the Primary Neurochemical Mechanism

Adenosine receptor antagonism provides the best-established pharmacological basis for the central effects of caffeine, but it should be distinguished from the mechanisms responsible for the specific scalp EEG changes summarized in this review. At concentrations associated with ordinary human consumption, caffeine acts predominantly as a competitive antagonist at adenosine receptors, particularly A1 and A2A receptors [1,2,81,82]. This pharmacological action is well supported independently of the present EEG literature. However, the included human EEG studies generally did not directly measure adenosine receptor occupancy, receptor-specific signaling, neurotransmitter release, or neuronal firing. Consequently, they cannot establish that a particular reduction in alpha, theta, delta, or total EEG power was caused specifically by blockade of A1 or A2A receptors.

The mechanistic framework presented here therefore separates established pharmacology from proposed receptor-to-EEG pathways. A1 receptor signaling can regulate neuronal excitability and neurotransmitter release, while A2A receptor signaling participates prominently in striatal and other circuits involved in arousal, motivation, and behavioral activation. These observations make it biologically plausible that caffeine-induced adenosine receptor antagonism alters the neuronal and network conditions from which scalp oscillations emerge. Nevertheless, the intermediate steps linking receptor blockade to changes in large-scale synchronization and ultimately to a specific scalp EEG frequency band remain incompletely demonstrated in humans. Thus, adenosinergic mechanisms provide a plausible upstream framework for interpreting caffeine-related EEG modulation rather than a receptor-specific explanation established by the included EEG studies.

A1 receptors are widely distributed throughout the cortex, hippocampus, thalamus, basal forebrain, and other regions involved in arousal and cognition. Their activation generally reduces neuronal excitability and neurotransmitter release. Consequently, caffeine-induced A1 blockade can increase the responsiveness of cortical and thalamocortical neurons and reduce the tendency toward highly synchronized activity associated with lower arousal states [83,84]. At the systems level, this effect contributes to maintenance of cortical responsiveness during wakefulness rather than producing a direct pharmacological action on any single EEG frequency band.

A2A receptors provide an additional and particularly important pathway for caffeine-induced arousal. They are strongly expressed in striatal and nucleus accumbens circuits involved in motivation, motor function, and behavioral activation. Experimental evidence from receptor-knockout models indicates that A2A receptors play a major role in caffeine-induced wakefulness, with subsequent studies localizing important components of this effect to A2A receptor-expressing neurons in the nucleus accumbens shell [85,86]. Thus, caffeine’s arousing effects arise not only from diffuse cortical disinhibition but also from modulation of specific subcortical circuits controlling wakefulness and behavioral activation.

A2A signaling is also closely related to dopaminergic function. A2A receptors interact antagonistically with dopamine D2 receptors, particularly within striatal medium spiny neurons. Blockade of A2A receptors can therefore facilitate D2-related signaling involved in motivation, effort, motor readiness, and alertness. Experimental evidence demonstrates functional interactions between A2A and D2 receptors, while human PET studies have shown increased striatal D2/D3 receptor availability following caffeine administration, with these changes associated with greater subjective alertness [87,88,89,90]. Caffeine therefore differs from classical monoamine-releasing psychostimulants: it does not require direct dopamine release to produce stimulant-like effects but can instead modify dopaminergic responsiveness by reducing adenosine-mediated opposition to D2 signaling.

The basal forebrain and related ascending arousal systems provide an additional link between adenosine pharmacology and waking cortical activity. The basal forebrain supplies widespread cholinergic projections to the cortex and plays a central role in cortical activation and attention. Adenosine accumulation in this region has been proposed as one molecular signal of increasing sleep pressure during sustained wakefulness. By blocking adenosine receptors, caffeine can preserve activity within wake-promoting basal forebrain–cortical systems and thereby oppose the transition toward more synchronized and sleep-prone cortical states [86,91]. This effect is consistent with caffeine’s ability to attenuate the electrophysiological expression of accumulating sleep pressure during prolonged wakefulness [9,92].

Caffeine also indirectly influences several other neurotransmitter systems, including noradrenergic, cholinergic, glutamatergic, GABAergic, histaminergic, and serotonergic pathways. These effects arise because adenosine receptors regulate neurotransmitter release and interact with interconnected wake-promoting systems [93]. Accordingly, caffeine should be regarded as a modulator of distributed arousal networks rather than as a compound that selectively increases or decreases one EEG rhythm. The band-specific consequences of this network modulation are considered separately in the following subsections.

Caffeine’s cerebrovascular actions are particularly important when electrophysiological findings are interpreted together with hemodynamic measures. Adenosine has vasodilatory properties, whereas caffeine can reduce cerebral blood flow through adenosine receptor antagonism. Human neuroimaging studies have demonstrated reductions in baseline cerebral blood flow and alterations in BOLD responses or functional connectivity after caffeine administration [94,95,96]. These vascular effects should be distinguished from changes in EEG, which reflects electrical activity more directly. Consequently, apparent caffeine-related alterations in fMRI global signal or connectivity may contain both neuronal and vascular components, whereas concurrent EEG can provide complementary information about changes in neural synchronization and vigilance.

The temporal profile of caffeine’s electrophysiological effects is also consistent with its pharmacology. Following oral administration, caffeine is rapidly absorbed and enters the central nervous system, allowing behavioral and physiological effects to emerge within tens of minutes. Its relatively long half-life, together with substantial interindividual variability in metabolism, means that receptor blockade and associated changes in arousal may persist for several hours. The magnitude and duration of the EEG response may therefore depend not only on dose but also on individual pharmacokinetics.

Habitual caffeine exposure further complicates interpretation. Chronic intake can produce adaptive changes within adenosinergic and downstream signaling systems, although evidence does not support a simple model in which tolerance is explained solely by upregulation of a single receptor population. Instead, tolerance likely involves multiple mechanisms, including changes in receptor density or sensitivity, intracellular signaling, compensatory neurotransmitter adaptations, sleep pressure, expectation, and withdrawal state [5,97,98]. This issue is especially important in experimental studies requiring habitual caffeine users to abstain before testing, because abstinence itself may alter vigilance and EEG activity. In such circumstances, caffeine administration may partly restore the participant toward a habitual caffeinated baseline rather than producing enhancement above a physiologically neutral state.

Finally, substantial individual variability should be expected despite a common receptor-level mechanism. Genetic variation affecting adenosine receptor sensitivity, particularly ADORA2A, and caffeine metabolism through CYP1A2 may influence the intensity and duration of caffeine responses. Baseline sleep pressure, habitual intake, age, sex-related factors, psychiatric status, nicotine exposure, and individual caffeine sensitivity may further modify the resulting electrophysiological pattern. Thus, caffeine’s fundamental mechanism is relatively consistent at the molecular level, but its expression at the level of large-scale neural networks and scalp EEG is strongly dependent on the biological and behavioral state of the individual.

### 4.3. Delta and Theta: State-Dependent Interpretation and Possible Links to Sleep Pressure

The present findings indicate that caffeine-related changes in delta and theta activity are best interpreted in relation to sleep pressure, vigilance instability, and drowsiness rather than as a uniform suppression of low-frequency EEG activity. Delta responses were less consistent than theta responses and were most apparent during fatigue, sleep deprivation, caffeine withdrawal, low-vigilance rest, or other conditions in which slow activity was already elevated. This pattern is compatible with sleep-homeostasis models in which sleep pressure accumulates during wakefulness and is reflected electrophysiologically by increasing slow-wave activity [99,100]. Adenosinergic mechanisms contribute to this homeostatic process, and caffeine opposes their expression through A1 and A2A receptor antagonism [9,101,102]. Consequently, reductions in waking delta after caffeine are more plausibly interpreted as attenuation of sleep-pressure-related cortical slowing than as a direct pharmacological suppression of the delta rhythm.

The spatial distribution of delta activity may further explain why its caffeine response was inconsistent across studies. Homeostatic slow-wave responses are often particularly prominent over frontal regions [100,103], but sleep pressure does not necessarily develop uniformly across the cortex. Experimental studies indicate that local sleep-like slow waves can occur during behavioral wakefulness and may be associated with transient neuronal “off” periods, performance lapses, and reduced responsiveness [104,105,106,107]. Caffeine may therefore exert its clearest effects on delta when slow activity represents regional cortical fatigue or local sleep-like intrusion. In fully rested participants, by contrast, waking delta activity is relatively limited, leaving less opportunity for a detectable pharmacological reduction.

Theta showed a more consistent relationship with caffeine because waking theta is strongly associated with accumulating sleep pressure and declining vigilance. Theta activity increases during sustained wakefulness and is related to subjective fatigue and deterioration in alertness [108]. Waking theta has also been linked to subsequent slow-wave activity during sleep, supporting the view that the two measures reflect related aspects of homeostatic sleep regulation [109]. Sleep deprivation further increases low-frequency waking activity, particularly theta, and these changes accompany impaired reaction time, attentional instability, and vigilance lapses [108,109,110,111]. Similar increases in theta and alpha activity are observed during drowsiness in sustained-attention and driving contexts [112,113]. Thus, caffeine-related theta reductions during prolonged wakefulness or monotonous tasks are most consistently interpreted as attenuation of the electrophysiological drift toward drowsiness.

However, theta is not a unitary marker of reduced vigilance. The so-called “theta paradox” reflects the fact that theta oscillations are observed both during increasing sleep pressure and during cognitively demanding states [23]. Frontal-midline theta, in particular, is associated with cognitive control, conflict monitoring, working memory, uncertainty processing, and adaptive behavioral regulation [23,113]. The functional meaning of a caffeine-related theta change therefore depends on recording context. Reduced diffuse or posterior theta during quiet rest, sleep deprivation, or monotonous vigilance is compatible with reduced drowsiness, whereas preserved or increased frontal-midline theta during active cognitive performance may instead indicate sustained engagement of control-related networks. This distinction helps explain why caffeine reduced theta in several resting and low-vigilance paradigms but did not consistently suppress theta during task performance [23,114,115].

Caffeine withdrawal provides additional support for this state-dependent interpretation. In habitual consumers, abstinence was associated with increased theta activity, particularly over frontal and frontotemporal regions, whereas caffeine re-administration tended to normalize these changes. Such effects are compatible with adaptations in adenosinergic signaling and the emergence of greater sleepiness during caffeine deprivation [101,102]. Accordingly, some apparent acute caffeine effects may reflect reversal of withdrawal-related cortical slowing rather than enhancement beyond a non-withdrawn physiological baseline.

### 4.4. Alpha Activity: Contextual Interpretation, Cortical Desynchronization, Attentional Gating, and Thalamocortical Hypotheses

Alpha activity was the most consistently caffeine-sensitive EEG rhythm identified in the present review. Across multiple studies, caffeine reduced alpha power or amplitude, while several investigations additionally reported increases in peak, mean, or dominant alpha frequency. These effects should be considered related but physiologically distinct. Reduced alpha power primarily reflects altered oscillatory synchronization and cortical excitability, whereas increased alpha frequency may indicate a faster thalamocortical operating state associated with greater vigilance and attentional readiness [20,21,116,117,118,119].

Importantly, reduced alpha power should not be assigned a single functional interpretation across experimental paradigms. The meaning of alpha modulation depends on the recording condition, scalp region, baseline vigilance state, and temporal relationship to ongoing behavior. A reduction in posterior alpha during eyes-closed resting wakefulness may reflect a transition away from a highly synchronized resting state, whereas reduced prestimulus or task-related alpha may reflect changes in selective attention, sensory gating, anticipatory processing, or task engagement. Conversely, alpha increases may occur during internally directed attention or suppression of task-irrelevant information. Accordingly, caffeine-related alpha reductions are interpreted here as changes in oscillatory synchronization whose functional significance must be determined from the specific experimental context, rather than as a direct or universal marker of cortical activation, vigilance, or cognitive readiness.

The reduction in alpha power is consistent with the broader concept of cortical desynchronization. Alpha activity decreases when cortical networks become engaged by sensory input, movement preparation, cognitive effort, or selective attention [116,117]. Contemporary models further characterize alpha as a mechanism of functional inhibition and attentional gating rather than simply an “idling” rhythm. Higher alpha power can suppress processing in task-irrelevant cortical regions, whereas lower alpha power is generally associated with increased excitability and greater access to sensory or cognitive information [21,118,119,120]. Within paradigms in which alpha attenuation accompanies sensory engagement or attentional allocation, caffeine-related alpha suppression may be compatible with reduced large-scale synchronization or altered inhibitory gating. However, reduced alpha power is not itself a direct measure of cortical activation, and the same directional change may have different functional meanings depending on eye condition, cortical topography, baseline alpha level, and behavioral state.

Alpha activity is also spatially and temporally modulated according to behavioral demands, decreasing in some cortical regions processing attended information while increasing in regions involved in suppressing irrelevant input [119,120,121,122]. Therefore, reductions in tonic, prestimulus, and task-related alpha should not be treated as equivalent phenomena. In task paradigms, caffeine-related alpha changes may reflect altered attentional allocation, anticipatory processing, sensory gating, or task engagement, whereas resting-state reductions may primarily indicate a change in the synchronization of the ongoing resting rhythm. Consequently, caffeine-induced reductions in tonic or task-related alpha may facilitate sensory readiness or sustained engagement by reducing inhibitory gating. However, alpha suppression should not automatically be equated with improved neural efficiency. Alpha oscillations also organize the timing of cortical excitability, and their reduction may indicate greater activation without necessarily implying more efficient cognition [21,118]. This distinction may be particularly important at higher caffeine doses or in caffeine-sensitive individuals, in whom increased arousal can coexist with anxiety or excessive activation.

The thalamocortical organization of alpha provides an additional mechanistic framework. Alpha rhythms arise through interactions among cortical pyramidal neurons, thalamic relay nuclei, and the thalamic reticular nucleus [121,122,123]. These circuits can shift between relatively synchronized oscillatory states and more activated modes favoring sensory transmission and cortical responsiveness [124,125]. Through adenosine receptor antagonism and its downstream effects on wake-promoting systems, caffeine may bias thalamocortical activity toward the latter state [101,102]. This provides a plausible systems-level mechanism for the reduced prominence of synchronized alpha activity after caffeine.

The magnitude of this effect depends strongly on the baseline recording state. Eyes-closed wakefulness normally produces prominent posterior alpha activity, providing greater scope for pharmacological suppression. By contrast, eye opening, sensory stimulation, and cognitive task performance already reduce alpha activity, potentially limiting the additional effect attributable to caffeine. Scalp distribution is also relevant: posterior alpha changes may predominantly reflect modulation of visual and sensory readiness, whereas frontal or central changes may be more closely related to attentional, executive, or sensorimotor processes. Thus, differences in eye condition, baseline alpha level, and topography may account for part of the variability across studies.

Changes in alpha frequency should be distinguished from changes in alpha power. Individual alpha frequency is relatively stable but remains sensitive to vigilance, cognitive state, aging, fatigue, and attentional demands [20,126,127,128]. Faster alpha frequency has generally been associated with higher alertness and more efficient information processing, whereas slowing may occur during fatigue, drowsiness, aging, or neurological dysfunction [20,126,127,128]. The increases in peak or mean alpha frequency observed after caffeine may therefore represent an acceleration of the dominant thalamocortical rhythm and, in fatigue-related contexts, partial opposition to vigilance-related slowing.

Power and frequency may consequently provide complementary information. A reduction in alpha amplitude indicates diminished large-scale synchronization, whereas an increase in alpha frequency reflects a change in the temporal organization of the remaining rhythm. Their simultaneous occurrence may characterize a transition from relaxed, internally oriented wakefulness toward a faster and more externally responsive electrophysiological state. These outcomes should therefore be analyzed separately rather than combined under a single concept of “alpha activation.”

Habitual caffeine consumption, withdrawal, and individual sensitivity provide additional sources of variability. In habitual users, caffeine administration may partly normalize withdrawal-related changes in alpha frequency or synchronization rather than produce enhancement above a non-withdrawn baseline. Conversely, weak or absent alpha responses may occur when baseline alpha is already low, the caffeine dose is small, tolerance is substantial, or the experimental task itself strongly suppresses alpha. Occasional alpha increases are also not necessarily inconsistent with increased vigilance, because alpha can rise when attention is internally directed or when task-irrelevant information must be actively inhibited [21,118,119,120].

### 4.5. Beta Activity: Context-Dependent Modulation and Possible Corticostriatal and Sensorimotor Mechanisms

Beta activity showed one of the most heterogeneous responses to caffeine in the present review. Some studies reported increases in beta power, beta2 activity, dominant beta frequency, or higher-frequency coherence, whereas others found reductions or no significant effects. These inconsistencies indicate that beta should not be regarded as a simple electrophysiological marker of stimulation. Instead, its response appears to depend on baseline vigilance, task demands, spectral definition, scalp region, habitual caffeine exposure, and whether caffeine is counteracting fatigue or withdrawal-related changes.

This interpretation is consistent with the functional diversity of beta oscillations. Beta rhythms, conventionally spanning approximately 13–30 Hz, participate in sensorimotor, cognitive, and perceptual processes and cannot be reduced to general arousal [129,130,131]. One influential account proposes that beta activity contributes to maintenance of the current sensorimotor or cognitive state—the so-called “status quo” function [129]. From this perspective, increases in beta after caffeine may reflect stabilization of an alert task set, sustained motor readiness, or preservation of ongoing cognitive control when fatigue or reduced vigilance would otherwise destabilize performance [129,130,131,132].

Sensorimotor mechanisms are particularly relevant because beta activity is prominent over central cortical regions and is strongly modulated by movement preparation, execution, inhibition, and post-movement recovery [116,133,134,135]. Movement preparation and execution are commonly associated with beta desynchronization, whereas post-movement beta rebound has been linked to restoration or stabilization of the sensorimotor state [116,133,134,135]. Consequently, an increase in central beta after caffeine may indicate enhanced tonic readiness or stabilization of sensorimotor networks, whereas a reduction during active processing may reflect greater engagement rather than diminished activation.

A similar principle applies to cognitive beta activity. Beta oscillations have been implicated in working memory, maintenance of cognitive sets, executive control, and top-down communication between cortical regions [130,131,136,137,138,139]. Beta-range feedback signals may contribute to the maintenance of task goals and regulation of sensory processing [137,138,139]. Thus, caffeine-related enhancement of beta activity may be particularly likely when sustained attention or cognitive stability must be maintained under challenging conditions. Conversely, if caffeine promotes active updating or processing rather than maintenance of the current state, beta power may decrease through task-related desynchronization [116].

The distinction between beta sub-bands may also account for part of the apparent inconsistency. Lower beta and higher beta activity may arise from partly different functional processes, and the reviewed studies did not use uniform frequency boundaries. Some investigations reported reductions in lower beta ranges, whereas others observed increases in beta2 power, beta2 frequency, or beta2/gamma coherence. Pooling these effects within a single broad 13–30 Hz category may therefore obscure physiologically meaningful differences. Future studies should report beta sub-bands separately and provide sufficiently detailed frequency definitions to permit comparison across experiments.

Topography provides another important interpretative dimension. Central beta changes are most readily linked to sensorimotor processes, whereas frontal beta may reflect executive control, response inhibition, or maintenance of task rules. Posterior beta activity may instead contribute to top-down modulation of sensory processing [136,138,140]. Accordingly, focal caffeine-related beta changes should not automatically be interpreted as evidence of generalized fast-frequency activation, because similar changes in different cortical regions may reflect distinct functional processes.

Beta should also be distinguished from more established electrophysiological markers of vigilance. Increases in theta and alpha activity are more consistently associated with progressive sleep pressure and declining alertness, whereas beta may reflect active wakefulness, compensatory control, or sensorimotor stabilization [23,141]. This distinction may explain why beta enhancement was observed more often in some studies involving fatigue, sleep deprivation, or reduced baseline vigilance: under these conditions, increased beta may represent an active mechanism supporting maintenance of performance rather than a direct measure of arousal itself.

Caffeine pharmacology provides a plausible background for these state-dependent effects without requiring a simple one-to-one relationship between receptor blockade and beta power. By antagonizing adenosine A1 and A2A receptors, caffeine reduces adenosine-mediated inhibition of wake-promoting systems and can influence dopaminergic and cortico-basal-ganglia signaling relevant to motivation and motor readiness [9,86,132]. Because beta oscillations are strongly represented in cortico-basal-ganglia and sensorimotor networks [116,129,133,134,135], these pathways may contribute to caffeine-related beta modulation. However, the direction of the EEG response ultimately depends on whether the prevailing functional process favors stabilization of ongoing activity or desynchronization associated with active processing.

Habitual caffeine consumption and withdrawal may further modify these effects. In low habitual consumers, beta enhancement may be more apparent because tolerance is limited, whereas in regular users responses may be smaller or dominated by reversal of abstinence-related changes. Baseline vigilance and task context therefore remain essential for distinguishing a genuine fast-frequency enhancement from normalization of an initially slower or less stable electrophysiological state.

### 4.6. Total EEG Power: Caffeine Reduces Large-Amplitude Synchronized Activity More Often than It Increases Total Activation

The present review indicates that caffeine more often reduces total EEG power, broad-band amplitude, or integrated spectral power than produces a generalized increase in EEG amplitude. Although this pattern may initially appear counterintuitive for a psychostimulant, EEG power should not be equated with the overall level of neuronal activity. Wakeful cortical activation is commonly characterized by relatively low-amplitude, desynchronized activity, whereas reduced vigilance and sleep are associated with greater synchronization and higher-amplitude rhythmic activity, particularly in slower frequency ranges [22,142,143,144]. Thus, a reduction in total EEG power after caffeine is physiologically compatible with increased arousal.

This interpretation is particularly relevant when total power is dominated by alpha, theta, or delta activity. Caffeine-related suppression of synchronized alpha during relaxed wakefulness or attenuation of theta and delta during drowsiness can lower overall spectral power without implying reduced cortical responsiveness. EEG amplitude depends strongly on the temporal synchronization of neuronal populations: large scalp-recorded potentials can arise when many aligned cortical neurons fluctuate synchronously, whereas highly active but less phase-aligned populations may generate lower-amplitude scalp signals [142,143,145,146]. Total EEG power therefore reflects the organization and synchrony of neural activity rather than a direct measure of neuronal firing or metabolic “energy.”

The concept of cortical desynchronization provides a useful framework for interpreting this pattern. Sensory, cognitive, and motor engagement can reduce the power of ongoing rhythmic activity as cortical networks shift from synchronized background states toward more dynamically responsive configurations [116,144]. Caffeine may produce a comparable tonic shift in some recording conditions, particularly when baseline EEG contains prominent synchronized rhythms. Importantly, this does not imply that every frequency band must decrease simultaneously; the same activated state may involve suppression of dominant slow or alpha rhythms alongside preservation or enhancement of faster activity.

The pharmacological basis for such a shift is consistent with caffeine’s antagonism of adenosine A1 and A2A receptors and the consequent reduction of adenosine-mediated sleep-promoting influences [9,102]. Through its effects on interconnected arousal systems, including pathways relevant to cortical and subcortical activation, caffeine can favor a less synchronized waking state [22,86,143,144,147]. Because these mechanisms have been considered in detail above, the important point for total EEG power is that increased arousal need not manifest as a global increase in spectral amplitude.

At the same time, total power is an intrinsically nonspecific EEG measure. An equivalent reduction in overall power can arise from different physiological changes: reduced delta or theta may reflect attenuation of drowsiness or sleep pressure, reduced alpha may indicate altered synchronization or inhibitory gating, and reduced beta may accompany active task-related desynchronization. Conversely, caffeine can modify spectral composition without changing total power when reductions in one frequency range are offset by increases in another. Consequently, the absence of a significant total-power effect does not imply an absence of electrophysiological modulation.

Recording condition further influences this measure. Eyes-closed wakefulness typically contains prominent posterior alpha activity and therefore higher baseline spectral power than eyes-open or cognitively engaged states. Caffeine may consequently produce larger total-power reductions when baseline activity is highly synchronized, whereas additional reductions may be smaller when eye opening or task performance has already desynchronized the EEG. Regional effects should likewise be interpreted cautiously because posterior, frontal, and central reductions may arise from different combinations of alpha, slow-wave, and sensorimotor activity.

Finally, reductions in total EEG power should not automatically be interpreted as evidence of improved cognitive functioning. Increased activation and reduced synchronization may facilitate responsiveness in some circumstances, but the relationship between cortical arousal and behavioral performance is not necessarily linear [148]. The functional meaning of a power reduction therefore depends on dose, baseline vigilance, task demands, individual sensitivity, and the accompanying behavioral response.

### 4.7. Dose-Response Patterns and the Influence of Habitual Caffeine Use

Across the evidence base, caffeine dose did not show a simple or consistently monotonic relationship with wakeful EEG effects. Alpha suppression was observed after relatively low doses, including 50 mg in some paradigms [34,77], as well as after commonly studied fixed doses of approximately 200–400 mg [31,32,36,38,51,52]. Higher doses did not invariably produce proportionally larger alpha effects. In the direct comparison of 200 and 400 mg caffeine, both doses reduced theta and alpha activity during relaxed wakefulness, while some task-related alpha effects were stronger after 200 mg than after 400 mg [32]. Similarly, both 250 and 500 mg reduced broad-band, alpha, and beta amplitude in another dose-comparison study, but the 500 mg condition did not produce a proportionally greater electrophysiological response [74]. In a weight-adjusted dose-response study, relative alpha power did not change significantly, whereas dominant alpha frequency increased only at the highest dose of 6.0 mg/kg [50]. Taken together, these findings do not support a simple model in which increasing caffeine dose produces progressively greater alpha suppression.

Dose-related patterns in theta and beta activity were similarly heterogeneous. One study reported a clear dose-related reduction in theta power after 250 and 500 mg caffeine, with the larger and more persistent reduction following 500 mg [48], and broader spectral suppression was observed after 400 than 200 mg in another experiment [32]. However, such monotonic effects were not reproduced consistently across the evidence base. Beta activity did not emerge preferentially at a single high-dose threshold: beta enhancement was reported after 250 mg in regular smokers and coffee drinkers [57] and after 600 mg slow-release caffeine during prolonged sleep deprivation [58], whereas other studies using 250–500 mg reported reduced beta power or amplitude [74,78]. Therefore, higher doses cannot be interpreted as producing a general shift toward beta-dominant activity. Anxiety-related studies likewise tended to use moderate-to-high fixed doses, including 250 and 500 mg [78], but the small number of such studies and their clinical heterogeneity do not permit a conclusion that anxiety-related EEG effects become systematically more frequent as dose increases.

The available literature also does not demonstrate a systematic difference between fixed-dose and body-mass-adjusted administration. Most studies used fixed doses, whereas a smaller subset administered caffeine in mg/kg. Because dosing strategy was confounded with differences in participants, habitual intake, experimental paradigm, recording condition, and EEG outcomes, the two approaches cannot be compared directly. Habitual caffeine consumption further complicates apparent dose-response relationships. Acute effects may be attenuated by tolerance in regular consumers, while abstinence before testing can create withdrawal-related slowing that is subsequently reversed by caffeine. Consequently, the same nominal dose may represent a substantially different pharmacological challenge in a low habitual consumer than in a tolerant participant undergoing caffeine withdrawal [43,59,70].

Several findings are compatible with non-monotonic dose-response relationships, but the evidence is insufficient to establish a general inverted-U model. Behavioral performance was better after 200 than 400 mg in one study despite broader EEG suppression at 400 mg [32]; another study found behavioral improvement at 1.5–3.0 mg/kg but not at 6.0 mg/kg despite more evident changes in dominant EEG frequency at the higher dose [50]; and behavioral enhancement was greater after 250 than 500 mg in another experiment despite EEG amplitude reductions at both doses [74]. A quadratic dose-response in nonlinear EEG complexity was also reported across escalating doses of 100–600 mg [65]. However, the latter observation derives from a single study with only 10 participants and should be regarded as hypothesis-generating rather than evidence for a general inverted-U electrophysiological response to caffeine. More broadly, the included studies were generally small, with a median analyzed sample of 16 participants, and most were designed to detect caffeine-versus-placebo effects rather than adequately powered dose-by-dose differences. The available evidence therefore supports dose as an important moderator but does not establish a reproducible monotonic, threshold, or inverted-U dose-response function for wakeful EEG.

### 4.8. Coherence and Asymmetry: Caffeine May Affect Network Synchronization, but Evidence Remains Preliminary

Evidence concerning caffeine-related EEG coherence and hemispheric asymmetry should be regarded as preliminary and hypothesis-generating. Only a small number of included studies assessed these outcomes, using heterogeneous recording and analytical procedures, and scalp coherence does not provide a direct measure of anatomical or synaptic connectivity. Consequently, observed changes can demonstrate altered statistical relationships between recorded signals but cannot establish that caffeine strengthened or weakened causal communication between underlying neuronal populations.

Coherence and hemispheric asymmetry were examined far less frequently than spectral power in the included studies, and the available evidence therefore remains preliminary. Caffeine-related changes in coherence appeared to depend on frequency, scalp region, and caffeine state. Withdrawal was associated with increased anterior alpha/theta coherence and decreased posterior theta/delta coherence, with partial normalization after caffeine re-administration, whereas another study reported increased beta2/gamma coherence following acute caffeine exposure. By contrast, hemispheric asymmetry findings were inconsistent, with no clear general pattern across withdrawal, nicotine–caffeine interaction, and clinical paradigms. These observations suggest that caffeine may influence the spatial organization of waking EEG, but the current evidence is insufficient to support a general statement that caffeine uniformly increases or decreases functional connectivity.

EEG coherence quantifies the stability of frequency-specific relationships between signals recorded at different locations and is commonly used as an indirect measure of functional coupling [149,150]. Synchronization between neuronal populations has been proposed as an important mechanism for distributed neural communication, and the communication-through-coherence framework suggests that effective interaction depends partly on temporal alignment of neuronal excitability cycles [139,151]. Accordingly, caffeine-related coherence changes could reflect altered coordination between cortical regions rather than merely changes in local oscillatory amplitude. However, coherence itself is not synonymous with more efficient communication: increases may reflect stronger functional integration, but they may also indicate excessive synchronization, whereas decreases can reflect either impaired coupling or greater functional segregation [139,149,150,151,152].

The functional interpretation of coherence also depends strongly on frequency. Alpha and theta synchronization may participate in attentional gating, inhibitory regulation, and large-scale coordination [21,119,153], whereas beta-range coupling has been associated with maintenance of cognitive or sensorimotor states, top-down control, and long-range feedback communication [129,137,138]. Gamma-range coupling may contribute to local processing and feedforward communication, although scalp-recorded gamma is particularly susceptible to electromyographic and other high-frequency artifacts [139,151,154]. Consequently, the reported increase in beta2/gamma coherence after caffeine should be interpreted cautiously as a potential alteration of fast-frequency network coordination rather than as a simple marker of greater arousal.

The withdrawal findings may represent a different network state. Increased frontal alpha/theta coherence during abstinence may reflect stronger synchronization within anterior networks during a period of reduced vigilance, whereas decreased posterior slow-frequency coherence may indicate altered organization of posterior activity. Normalization after caffeine re-administration is compatible with restoration of the habitual waking network configuration rather than a uniform global increase in connectivity. Although these effects can be considered within the broader context of caffeine’s antagonism of adenosine-mediated sleep pressure [9,101,102], the present coherence data do not establish a direct receptor-to-connectivity mechanism.

Methodological limitations are particularly important when interpreting scalp EEG coherence. Volume conduction can produce apparent zero-lag correlations between electrodes even when the underlying cortical sources are not functionally interacting [155,156,157]. To reduce this problem, methods such as the imaginary component of coherency, phase-lag index, and related phase-lagged measures have been developed to emphasize interactions that cannot easily be explained by instantaneous field spread [155,156,157]. Reference choice also substantially influences coherence estimates; average reference, linked mastoids, Cz reference, Laplacian derivations, and source-space approaches can yield different patterns. These issues are especially relevant when comparing older caffeine studies conducted with heterogeneous montages and preprocessing pipelines. Future investigations should therefore favor high-density EEG, source reconstruction, and connectivity metrics that minimize zero-lag and reference-related contamination.

Hemispheric asymmetry requires a different interpretative framework. Frontal alpha asymmetry has been studied in relation to affective style, approach–withdrawal motivation, anxiety, depression vulnerability, and emotion regulation [158,159,160,161,162]. Because alpha power is often inversely related to cortical activation, relative left–right differences have traditionally been interpreted as reflecting asymmetric cortical engagement [158,159,160]. However, this interpretation is indirect and should not be treated as a simple measure of hemispheric activation.

The weak caffeine-related asymmetry findings are therefore unsurprising. Caffeine acts systemically and its primary pharmacological effects are not inherently lateralized; any asymmetry change is more likely to emerge through interactions with emotional state, motivation, nicotine exposure, task demands, or pre-existing individual differences. Moreover, resting frontal asymmetry may be less informative than asymmetry elicited during emotionally or motivationally relevant challenges. The capability model proposed by Coan and colleagues emphasizes that frontal asymmetry may become most meaningful when individuals are placed in contexts that recruit affective or motivational systems rather than during passive rest [160]. Future caffeine studies may therefore obtain more interpretable asymmetry effects from challenge paradigms than from resting EEG alone.

Reliability and preprocessing further constrain asymmetry measures. Frontal asymmetry estimates can depend on reference montage, recording duration, individual alpha frequency, artifact correction, and whether EEG is recorded at rest or during an emotional or cognitive challenge [161,162,163]. Hagemann and colleagues highlighted important reliability and validity limitations of anterior asymmetry as an individual-difference measure [161], while subsequent work has reinforced the importance of recording context and reference choice [162,163]. Thus, the absence of a consistent caffeine effect on resting asymmetry should not be interpreted as definitive evidence that caffeine cannot influence lateralized cortical processes.

### 4.9. EEG Microstates and Nonlinear Complexity: Caffeine May Reorganize Brain-State Dynamics Beyond Spectral Power

EEG microstates and nonlinear complexity provide potentially informative but currently preliminary perspectives on caffeine-related EEG modulation. These measures describe properties of scalp-field configuration and signal dynamics that are not captured by conventional spectral power; however, they should not be interpreted as direct measurements of neuronal network organization, information processing efficiency, or underlying molecular mechanisms. In the present evidence base, each of these domains was represented by very few studies, and mechanistic interpretation is therefore necessarily hypothesis-generating.

The available evidence suggests that caffeine may influence aspects of waking EEG organization that are not fully captured by conventional spectral power. EEG microstates and nonlinear complexity provide complementary approaches to this question: microstate analysis characterizes the temporal sequence of quasi-stable global scalp-field configurations, whereas complexity measures quantify the irregularity, dimensionality, or information content of the EEG signal. These approaches therefore address the temporal organization of neural activity rather than simply determining whether power within a particular frequency band increases or decreases [25,26,164,165].

EEG microstates persist for tens to hundreds of milliseconds before transitioning rapidly between relatively stable spatial configurations and have therefore been proposed as electrophysiological representations of transient large-scale brain states [25,26,164,165]. Their duration, occurrence, coverage, and transition structure provide information about the stability and sequencing of whole-brain activity. Simultaneous EEG–fMRI studies further indicate that different microstate configurations are associated with large-scale resting-state networks, while microstate sequences display structured, scale-free temporal dynamics [166,167]. Caffeine-related changes in microstate parameters could therefore indicate altered temporal organization of distributed brain networks rather than merely modulation of local oscillatory amplitude.

However, the caffeine-specific microstate evidence remains extremely limited. Only one included study examined this outcome, and it involved individuals with early-phase schizophrenia rather than a representative healthy sample. Caffeine reduced class D duration, occurrence, and contribution while increasing selected class A and B parameters. This finding is potentially important because schizophrenia itself has repeatedly been associated with abnormalities in microstate organization, including reduced class D parameters and alterations in other microstate classes [168,169,170,171]. The observed caffeine response may therefore reflect modulation of an already atypical network state rather than a general effect that can be extrapolated to healthy individuals.

Interpretation of individual microstate classes should also remain cautious. Class D has frequently been related to attention or frontoparietal control processes, whereas classes A and B have been associated with auditory/verbal and visual networks, respectively, but these functional assignments are approximate rather than one-to-one mappings [26,166,168,172]. Moreover, microstate parameters depend on methodological choices such as the number of extracted classes, clustering procedures, global-field-power peak selection, reference scheme, data length, smoothing, and artifact correction [25,26,172]. Consequently, replication using standardized pipelines, larger samples, and both healthy and clinical populations is required before a caffeine-specific microstate signature can be proposed.

Nonlinear complexity provides a different description of brain-state organization. Measures such as correlation dimension, approximate entropy, sample entropy, multiscale entropy, Lempel–Ziv complexity, permutation entropy, and related fractal or nonlinear indices quantify aspects of temporal variability, predictability, and dimensionality that are not represented directly by conventional power spectra [173,174,175,176,177,178]. Such measures have been shown to vary with cognitive load, emotional state, and other changes in mental activity, demonstrating that EEG complexity can capture state-dependent neural dynamics beyond frequency-band amplitude [179,180,181].

The caffeine-related complexity evidence in the present review suggests a nonlinear rather than monotonic response. Complexity did not simply increase with greater stimulation; instead, its direction depended on dose, task session, and cognitive demands. This is consistent with the broader observation that EEG complexity is sensitive to arousal and conscious state but does not have a universally beneficial direction. Wakefulness generally exhibits richer and more differentiated temporal dynamics than sleep, anesthesia, or other states of reduced consciousness, although the magnitude and direction of individual complexity measures depend on methodology and temporal scale [176,177,178,182]. Accordingly, an optimal caffeine response may involve reorganization of temporal dynamics rather than a simple increase in complexity.

Microstates and nonlinear complexity may therefore provide complementary information about caffeine-induced brain-state changes. Microstates characterize which global configurations occur and how the brain transitions between them, whereas complexity measures characterize how variable, predictable, or dynamically rich the underlying EEG signal is. Combining these approaches could help determine whether apparently heterogeneous changes in alpha, theta, beta, or total spectral power arise from a more coherent alteration in large-scale temporal organization. This possibility is particularly relevant because conventional spectral findings in the present review were strongly dependent on vigilance state, task demands, and methodological conditions.

## 5. Limitations and Future Directions

The present review has several limitations that should be considered when interpreting the available evidence. First, the included literature was characterized by substantial clinical and methodological heterogeneity. Studies differed in caffeine dose and formulation, timing of EEG acquisition after administration, duration of pre-study abstinence, habitual caffeine consumption, participant characteristics, vigilance state, experimental paradigm, and control condition. EEG methodology also varied considerably, including eyes-open versus eyes-closed recording, electrode number and montage, reference scheme, scalp regions analyzed, preprocessing procedures, frequency-band definitions, and whether outcomes were expressed as absolute power, relative power, amplitude, dominant frequency, or topographic measures. These differences limited direct comparability between studies and precluded a meaningful quantitative meta-analysis, including within nominally similar subgroups. Future investigations would benefit from harmonized acquisition and reporting standards that permit comparable effect estimates across laboratories.

Second, the evidence base was dominated by relatively small studies and by healthy young adults, while children, older adults, clinical populations, athletes, smokers, individuals with sleep disorders, and caffeine-sensitive participants were represented in substantially fewer investigations. Several studies also used sex-specific samples or relatively narrow participant groups. Consequently, the findings cannot automatically be generalized across age groups, clinical conditions, or levels of habitual caffeine exposure. Larger, adequately powered studies with more diverse samples are needed, together with prespecified analyses of age, sex, baseline vigilance, sleep history, and clinical status as potential moderators.

Habitual caffeine consumption and withdrawal represent particularly important sources of uncertainty. Participants described as habitual users were exposed to widely different baseline amounts of caffeine, and abstinence requirements varied substantially between studies. In regular users, pre-experimental abstinence may induce sleepiness, fatigue, and EEG slowing, meaning that subsequent caffeine administration can partly reverse withdrawal rather than enhance neural function above a genuinely caffeine-free baseline. Future placebo-controlled studies should therefore quantify habitual intake, standardize abstinence procedures, assess withdrawal symptoms directly, and distinguish pharmacological enhancement from withdrawal reversal. Designs incorporating both low- and high-habitual consumers may be especially informative.

Individual biological variability is another insufficiently characterized source of heterogeneity. Genetic variation affecting caffeine metabolism and adenosine signaling, particularly CYP1A2 and ADORA2A-related mechanisms, may influence caffeine pharmacokinetics, anxiety sensitivity, sleep disruption, and ultimately EEG responsiveness. Most of the existing EEG literature has not integrated genetic or metabolic information. Future studies combining EEG with genotyping, standardized caffeine dosing, and pharmacokinetic measures could help determine whether apparently inconsistent electrophysiological responses represent biologically distinct responder profiles rather than experimental noise.

Caffeine-induced changes in cerebral blood flow also deserve greater consideration, particularly in multimodal EEG–fMRI research. Adenosine receptor antagonism can reduce baseline cerebral blood flow and modify the vascular component of BOLD signals [92,93,94]. EEG is not dependent on the hemodynamic response in the same manner as fMRI, but interpretation becomes more complex when electrophysiological and BOLD findings are compared directly. Future multimodal studies should ideally quantify cerebral perfusion or otherwise account for caffeine-related vascular effects so that changes in BOLD connectivity or global signal can be separated more clearly from changes in neuronal oscillatory activity.

Another limitation is that conventional spectral power overwhelmingly dominates the existing literature. Connectivity, asymmetry, nonlinear complexity, microstates, and other dynamic EEG measures were examined in relatively few studies, limiting confidence in conclusions concerning large-scale network organization. These methods are also particularly sensitive to preprocessing choices, reference schemes, volume conduction, artifact contamination, and analytic pipelines. Future work should use standardized high-density EEG acquisition and reproducible preprocessing while incorporating source-space or phase-lagged connectivity, individualized frequency bands, aperiodic spectral parameters, microstate dynamics, and nonlinear complexity measures. Combining these approaches with behavioral measures of vigilance and cognitive performance may clarify whether apparently heterogeneous spectral effects reflect a more consistent underlying alteration of brain-state organization.

Finally, future research should move toward adequately powered, preregistered, placebo-controlled crossover designs with standardized caffeine doses and timing, explicit reporting of habitual consumption and withdrawal status, appropriate washout periods, and repeated EEG assessments across the caffeine time course. Simultaneous assessment of subjective alertness, vigilance performance, sleep pressure, and pharmacokinetic variables would allow electrophysiological changes to be linked more directly to functional outcomes. Such standardization is necessary to determine which EEG measures represent robust markers of caffeine exposure, which primarily reflect reversal of sleepiness or withdrawal, and which are strongly dependent on individual neurobiological characteristics.

The present review also has several methodological strengths. It was prospectively registered, used predefined eligibility and outcome-selection criteria, searched multiple major bibliographic databases from inception, and employed independent screening, data extraction, and risk-of-bias assessment by two reviewers. A further strength was the explicit separation of conventional time-domain ERP outcomes, sleep EEG, and non-ERP wakeful EEG, thereby reducing conceptual overlap with Parts 1 and 2 of The Caffeinated Brain [79,80]. The synthesis also considered both conventional spectral measures and less frequently examined outcomes such as functional connectivity, asymmetry, nonlinear complexity, microstates, and multimodal EEG–fMRI measures. Finally, habitual caffeine intake, preceding abstinence, withdrawal, vigilance state, eye condition, task context, and risk of bias were treated as interpretative moderators rather than assuming that all caffeine–EEG experiments represented an equivalent physiological condition.

## 6. Conclusions

Caffeine-related changes in waking EEG are heterogeneous and strongly dependent on experimental context. Reductions in alpha power or amplitude were among the more frequently reported findings after acute caffeine administration, although they were not universal and varied according to eye condition, scalp region, alpha sub-band, dose, baseline vigilance, task demands, habitual caffeine intake, and withdrawal status. In several studies, caffeine also increased mean, peak, or dominant alpha frequency, but these frequency changes should be considered separately from changes in alpha power.

Reductions in theta, delta, or broader slow-frequency activity were particularly evident under conditions of increased sleep pressure, prolonged wakefulness, sleep deprivation, fatigue, monotonous vigilance, or caffeine withdrawal. These findings are compatible with attenuation of drowsiness- or withdrawal-related EEG slowing, but they do not support a universal slow-frequency response to caffeine. Beta activity was considerably more heterogeneous, with increases, decreases, null effects, and condition-dependent responses reported across studies. Total EEG power also frequently decreased, although this effect depended on baseline state and spectral composition.

Overall, the available evidence does not establish a single reproducible EEG signature or validated electrophysiological biomarker of caffeine response. Alpha suppression should therefore be regarded as a recurring empirical finding rather than as a specific or validated marker of caffeine-induced cortical activation, vigilance, or cognitive readiness. The direction and magnitude of EEG effects remain dependent on study design, caffeine dose and timing, habitual consumption, abstinence and withdrawal, vigilance state, eye condition, task demands, participant characteristics, frequency-band definitions, scalp topography, and EEG methodology.

Importantly, caffeine-related EEG changes should not be interpreted as direct indicators of improved cognitive performance. Studies reporting both electrophysiological and behavioral outcomes showed only partial concordance, with EEG changes sometimes occurring without measurable behavioral improvement and with electrophysiological and behavioral effects differing in magnitude or temporal profile. Evidence concerning coherence, asymmetry, nonlinear complexity, EEG microstates, network-level organization, and EEG–fMRI relationships remains preliminary because these outcomes were examined in relatively few and heterogeneous studies. Future adequately powered and standardized studies are needed to determine whether any EEG measure demonstrates sufficient reproducibility, specificity, reliability, and functional validity to serve as a biomarker of caffeine response.

## Figures and Tables

**Table 1:** Studies included in review.

Study			Participants				Caffeine Protocol					EEG Methods				Outcomes		
Study	Study Design	Country	Total Sample Size/Analyzed Sample Size	Age and Sex Distribution	Healthy or Clinical Status	Habitual Caffeine Consumption	Abstinence Duration	Caffeine Formulation and Dose	Comparator	Randomization and Blinding	Time Between Administration and EEG Recording	Eyes-Open/Eyes-Closed/Task Condition	Number of EEG Channels	Electrode Montage and Reference	EEG Bands	Primary EEG Outcome	Effect Estimate and Measure of Precision	Principal Behavioral Outcome
[31]	Randomized, double-blind, placebo-controlled crossover	Germany	10/10	22–33 y; mean 25 ± 4; 10 male	Healthy volunteers	Moderate; 1–2 cups coffee/day	10 h	Oral caffeine powder in 150 mL water; 200 mg	Glucose placebo	Randomized; double-blind	30 min	Resting eyes open and eyes closed; 15 min each	17	International 10–20; bipolar recording; Cz physical reference	Delta: 1.25–4.50 Hz Theta: 4.75–6.75 Hz Alpha1: 7.00–9.50 Hz Alpha2: 9.75–12.50 Hz Beta1: 12.75–18.50 Hz Beta2: 18.75–35.00 Hz	Reduced total EEG power, especially alpha1, alpha2 and beta1; effects stronger with eyes open	Medians (ranges); *p* < 0.05	
[32]	Placebo-controlled within-subject pilot	Germany	10/10 EEG; 9 behavioral	Mean 21 y; 10 male	Healthy volunteers			Oral tablet with mineral water; 200 mg and 400 mg	Mineral water placebo		Rest: ~15 min; KLT: ~30 min	Relaxed eyes-open rest and Konzentrationsleistungstest (KLT)	17	International 10–20; Cz physical reference	Delta 1.25–4.50 Hz, theta 4.75–6.75 Hz, alpha1 7.00–9.50 Hz, alpha2 9.75–12.50 Hz, beta1 12.75–18.50 Hz, and beta2 18.75–35.00 Hz.	Decreased spectral power at rest, mainly theta and alpha; broader reductions at 400 mg; smaller occipital alpha increase during KLT	Post-treatment values as % of baseline; medians; >90% median-containing intervals; sign test	KLT problems solved/time and % correct; tendency best at 200 mg and lower at 400 mg than placebo
[33]	Randomized, double-blind, placebo-controlled crossover	Thailand	13/13	18–25 y; mean 20 ± 1; 13 male	Healthy university-level Taekwondo athletes	Low intake criterion <150 mg/day; Results: 3.43 ± 0.35 mg/kg	Methods: ≥24 h; Results: ≥12 h	Two oral capsules; 200 mg total; ~3 mg/kg	Visually identical placebo	Randomized; double-blind	Methods: ~60 min; abstract: 30 min	5-min resting eyes open; auditory-oddball/P300 assessments also performed	32	Midline analyses at Fpz, Fz, Cz, Pz, Oz (POz also reported)	delta 0.5–4 Hz, theta 4.5–8 Hz, alpha 8.5–13 Hz, and beta 13.5–29.5 Hz	Reduced frontal delta absolute power after caffeine; P300 amplitude changes also reported	Fpz delta interaction F(2,48) = 4.730, *p* = 0.013, partial η^2^ = 0.16; post: 74.8 ± 5.6 vs. 102.2 ± 8.8 µV^2^ (mean ± SEM); P300 between-condition *p* = 0.028 at Cz/Pz with 95% CIs reported	No significant change in oddball reaction time, accuracy/error rate, or Taekwondo agility
[34]	Randomized, double-blind, placebo-controlled crossover	Thailand	25/25	Mean 21 ± 2; 25 male	Healthy Southeast Asian university students	Low/mild; ≤~1–2 cups coffee/tea or 1 cola/day	≥12 h	Caffeine in 150 mL flavored water; 50 mg; ~0.7 mg/kg	Same flavored drink without caffeine	Randomized by coin flip; double-blind	30 min; ~35 min after post-exercise recording	Eyes open and eyes closed acquired; only eyes-closed data analyzed	32	International 10–20; average mastoids (A1 + A2)/2; analysis focused on Fpz, Fz, Cz, Pz, Oz	delta 0.5–4 Hz, theta 4.5–8 Hz, alpha 8.5–13 Hz, beta 13.5–29.5 Hz	Suppressed alpha power after caffeine versus placebo	Change from baseline (mean ± SEM): Fpz −8.5 ± 9.4% vs. +26.9 ± 13.4%; Fz −4.3 ± 10.1% vs. +45.6 ± 18.7%; Cz −1.3 ± 10.1% vs. +45.5 ± 16.7%; Pz +0.1 ± 13.4% vs. +65.4 ± 28.7%; Oz −3.3 ± 11.6% vs. +76.5 ± 26.8%; *p* = 0.01–0.04	Improved TMT-B; also TMT-A and forward digit span improved
[35]	Mixed experimental; menstrual-cycle phase between-subject, caffeine within-subject	Canada	80 recruited/56 EEG	18–40 y; mean 25.09 ± 6.28; all female	Healthy adults	Assessed with Caffeine Consumption Questionnaire (mg/week)	~9 h (from midnight)	Oral caffeine pill with water; 200 mg	Placebo pill	Randomized; double-blind; counterbalanced	~30 min	3-min resting eyes closed	32	10–10; nose online reference; offline linked mastoids TP9/TP10; mid-forehead ground	delta 1–4 Hz; theta 4–8 Hz; alpha 8–12 Hz; alpha1 8–10 Hz; alpha2 10–12 Hz; beta 12–20 Hz.	Habitual caffeine intake moderated caffeine-induced alpha1 reduction	Drug × habitual intake F(1,51) = 6.54, *p* = 0.014, partial η^2^ = 0.11; caffeine–placebo alpha1 difference vs. habitual intake r = −0.36, *p* = 0.007	
[36]	Randomized, double-blind, placebo-controlled crossover	Australia	22/22	Mean 20.5 y (17–36); 17 female, 5 male	Healthy nonsmoking university students	Moderate; ~2–4 cups coffee/day	≥4 h before first session; same pattern before second	Gelatin capsule; 250 mg	Identical placebo capsule	Randomized; double-blind; counterbalanced	Analysis focused on ~26–32 min	Alternating 2-min eyes closed/eyes open; final EO–EC–EO periods analyzed	19	International 10–20; linked ears; ground between Fpz and Fz	Alpha 8–13 Hz	Reduced global alpha amplitude	F(1,20) = 8.11, *p* < 0.01, η^2^ = 0.29; ~12% alpha reduction; eye-condition interaction F = 2.09, *p* = 0.164, η^2^ = 0.09	
[37]	Randomized, double-blind, placebo-controlled repeated-measures crossover	Canada	27/27	Schizophrenia: 21–38 y, 27.43 ± 3.88, 11 M/3 F; controls: 19–35 y, 23.23 ± 4.28, 9 M/4 F	14 clinically stable early-phase schizophrenia; 13 healthy controls	Habitual users; CCQ 1490.0 ± 1201.4 (schizophrenia) and 1250.1 ± 1398.8 (controls)	From midnight until morning testing	Identical pill; 200 mg	Identical placebo pill	Double-blind; order counterbalanced	30 min	3-min eyes open then 3-min eyes closed	64	10–10; analyses at F3/F4/P3/P4; bilateral mastoid electrodes applied	delta 1.5–4.0 Hz, theta 4.0–8.0 Hz, alpha was subdivided into alpha1 (8.0–10.5 Hz) and alpha2 (10.5–13.0 Hz), while beta activity ranged from 13.0 to 20.0 Hz.	Reduced alpha1, alpha2 and beta power, with some diagnosis- and eye-condition-specific effects	Eyes open: alpha1 g = 0.24, *p* = 0.003; alpha2 g = 0.27, *p* = 0.002; beta g = 0.36, *p* = 0.005. Eyes closed: beta g = 0.26, *p* = 0.030; schizophrenia frontal alpha2 g = 0.44, *p* = 0.008	
[38]	Randomized, double-blind, placebo-controlled crossover	Brazil	15/15	21–38 y; mean 26 ± 5; 6 male, 9 female	Healthy, right-handed, nonsmoking volunteers	Moderate; 1–2 cups coffee/day	10 h	Gelatin capsule; 400 mg	Placebo capsule	Randomized; double-blind	30 min	5-min alert resting eyes closed	20	International 10–20; monopolar; linked earlobes; ground Fpz	delta 1.0–3.5 Hz; theta, 4.0–7.5 Hz; alpha, 8.0–12.0 Hz; and beta, 13–25 Hz.	Reduced log absolute EEG power, including alpha power	Overall 3.076 ± 0.84 placebo vs. 2.834 ± 0.74 caffeine; F(1478) = 16.8, *p* < 0.0001. Alpha F(1118) = 5.36, *p* < 0.05	Reaction time and Stroop: no significant caffeine–placebo differences
[39]	Randomized single-crossover within-subject pre/post	Saudi Arabia	12/12	20–30 y; mean 25.2 ± 2.8; 12 male	Healthy nonsmoking men	Moderate; 100–300 mg/day	≥12 h	Freshly brewed black coffee; 162 mg	Pre-caffeine baseline	Randomized	15 min	15-min resting state	32-channel system; 9 electrodes recorded; 7 analyzed	International 10–20; analyzed C3, Cz, C4, CP5, CP1, CP2, CP6; online left mastoid, offline average of remaining channels	delta 1–4 Hz, theta 4–8 Hz, alpha 8–13 Hz, beta 13–30 Hz	Reduced alpha power and increased beta power	Alpha −5.1 ± 0.8 to −6.9 ± 0.9 dB, t(11) = 2.31, *p* = 0.041, d = 0.67; beta −4.7 ± 1.2 to −2.3 ± 1.1 dB, t(11) = 3.59, *p* = 0.004, d = 1.04	
[40]	Open repeated-measures within-subject caffeine-withdrawal study	United States	13/13; reintroduction *n* = 10	18–48 y; ~4 male, 9 female	Healthy volunteers	≥300 mg/day	Withdrawal assessed on days 1, 2 and 4; 4-day abstinence period	Coffee reintroduction on day 4	Usual-caffeine baseline and withdrawal days 1, 2 and 4	Open-label	Reintroduction QEEG covered first ~30 min after coffee	Resting QEEG	21	International 10–20; monopolar; linked ears	delta was defined as frequencies below 3.5 Hz, theta 3.5–7.5 Hz, alpha 7.5–12.5 Hz, and beta frequencies above 12.5 Hz	Withdrawal increased theta/delta and reduced beta-related measures; changes tended to reverse after reintroduction	FP1 theta absolute-power Z-score 0.02 baseline, 0.58 day1, 0.92 day2, 0.86 day4; F = 6.85, *p* < 0.0001; 92.3% showed frontal theta voltage increase reported	Subjective withdrawal symptom severity (1+ to 4+), including headache, drowsiness, fatigue and impaired concentration
[41]	Randomized, double-blind, placebo-controlled crossover during sleep deprivation	Switzerland	12/12	Mean 25.3 y (20–30); 12 male	Healthy male students	<300 mg/day	2 weeks	Two 200-mg doses; 400 mg cumulative	Placebo	Randomized; double-blind	~1, 4, 7 h after first dose; ~1 and 4 h after second	Each session: 3 min eyes closed then 5 min eyes open; theta analysis based on eyes open	1 derivation reported	C3–A2	Theta 5–8 Hz	Reduced waking theta activity	1 h reduction: 22.5 ± 6.0% after evening dose and 31.8 ± 5.1% after morning dose; treatment × time F(1,10) = 4.8, *p* = 0.05; treatment F(1,10) = 29.0, *p* < 0.001	Stanford Sleepiness Scale: 3.2 ± 0.1 caffeine vs. 3.7 ± 0.2 placebo; F(1,11) = 5.8, *p* < 0.04
[42]	7-week double-blind within-subject crossover with chronic maintenance and acute challenge	United States	16/16	Mean 36.3 y (24–54); 12 female, 4 male	Generally healthy regular caffeine users	Mean 215 mg/day (82–404); eligibility ≥50 mg/day	Acute abstinence ~24 h after last active caffeine dose	Opaque gelatin capsules; 200 mg anhydrous caffeine; maintenance 200 mg twice daily (400 mg/day)	Matched placebo capsules: lactose + ~4.5 mg quinine sulfate	Double-blind; maintenance sequence counterbalanced	~90 min after afternoon capsule	3-min resting eyes closed	16	International 10–20; ear-tip reference	delta (0.4–3.9 Hz), theta (4.0–7.9 Hz), alpha1 (8.0–9.9 Hz), alpha2 (10.0–13.9 Hz), beta1 (14.0–24.9 Hz), and beta2 (25.0–40.0 Hz).	Theta highest and beta-2 lowest during acute caffeine abstinence	Theta F(3,45) = 5.81, *p* = 0.002; beta-2 F(3,45) = 5.93, *p* = 0.002; figures ±1 SEM	Withdrawal/mood symptoms: increased tiredness, fatigue, sluggishness and weariness; reduced vigor/energy
[43]	Single-blind randomized controlled pilot; parallel groups	Türkiye	37/37	18–25 y; mean 22.24 ± 0.33; 22 female, 15 male	University students	Low <3 mg/kg/day (*n* = 17) vs. high >3 mg/kg/day (*n* = 20)		Caffeinated coffee; 200 mg	Decaffeinated coffee	Randomized allocation; single-blind	1 h	5-min resting eyes open and 5-min resting eyes closed	19	International 10–20; Mitsar-EEG-201	delta, 0.5–3.9 Hz; theta, 4.0–7.4 Hz; alpha1, 7.5–9.9 Hz; alpha2, 10.0–13.9 Hz; beta1, 14.0–19.9 Hz; and beta2, 20.0–30.0 Hz.	Acute caffeine effects differed by habitual-use group: lower delta/theta and higher beta2 in low consumers; lower alpha1/alpha2 in high consumers	LC eyes closed: delta F = 19.504, *p* < 0.0001; theta F = 6.976, *p* = 0.009; beta2 F = 10.459, *p* = 0.001. HC eyes open: alpha1 F = 11.310, *p* = 0.001; alpha2 F = 17.835, *p* < 0.0001	MoCA: LC + caffeine increased 23.5 ± 1.43 SE to 26.1 ± 0.93, *p* = 0.038
[44]	Randomized crossover under usual-sleep and sleep-restriction conditions	Ireland	15/15	17–19 y; mean 18; 4 male, 11 female	Healthy volunteers	160–445 mg/day; mean 261 mg/day (~2–4 cups coffee/day)	Avoided other caffeine throughout 4-week study; placebo condition ~1 week abstinence	B.P. anhydrous caffeine in gelatin capsules; 1.75 mg/kg per dose, three times daily	Identical gelatin capsules with maize starch	Randomized Latin-square order	~50 min	5 min eyes closed, 5 min eyes open, and two 15-min sustained-attention tasks	32	International 10–20; average of all connected inputs	delta, 0.5–3.4 Hz; theta, 3.5–7.5 Hz; alpha, 7.6–12.0 Hz; and beta, 12.1–30.0 Hz	Reduced delta and increased beta power in several regions; no main theta or alpha effect	Delta: left frontal F(1,14) = 8.06, *p* < 0.05; mid-posterior F = 7.31, *p* < 0.05. Beta: left frontal F = 9.14, *p* < 0.01; left central F = 11.54, *p* < 0.01; right posterior F = 4.89, *p* < 0.05; interaction partial η^2^ up to 0.27	Random/Fixed SART accuracy; no significant caffeine effect, F(1,14) = 2.62, *p* > 0.05
[45]	Non-randomized parallel-group experimental study	United Kingdom	20/20	Poor sleepers: 37.3 ± 11.2; normal sleepers: 34.4 ± 12.6; each group 5 male/5 female	Otherwise healthy; poor sleepers vs. normal sleepers	Poor sleepers 436.3 ± 224.1 mg/day; normal sleepers 484.9 ± 359.6 mg/day	24 h	Caffeine citrate capsule; 2.5 mg/kg	Poor sleepers vs. normal sleepers receiving the same caffeine dose	Non-randomized	30-min intervals for 4 h after dosing	Resting eyes open/eyes closed; CNV reaction-time task; auditory oddball	Resting spectral EEG: 1 left-occipital channel; evoked potentials vertex-linked mastoids	Left occipital referenced to linked mastoids; CNV/AEP vertex-linked mastoids	delta: 0–4 Hz, theta: 5–7 Hz, alpha: 8–12 Hz, and beta: 13–35 Hz.	No significant effect on CNV, AEPs, peak alpha frequency, or resting delta/theta/alpha/beta power		DSST and tapping rate: no significant post-caffeine change; poor sleepers performed worse overall
[46]	Comparative non-randomized pharmacokinetic/electrophysiological study	Tunisia	8/8	Caffeine-sensitive: 38.7 ± 6.7 y (31–48); controls: 34.7 ± 6.3 y (29–45); all male	Healthy; 4 caffeine-sensitive with nocturnal insomnia and 4 non-sensitive controls	53.7 ± 11.9 mg/day sensitive vs. 456.7 ± 61.7 mg/day controls	≥36 h	Caffeine dissolved in 150 mL warm water; 140 mg	Caffeine-sensitive vs. non-sensitive participants	Non-randomized	Baseline and 1, 2, 3, 4, 5, 6 h post-dose		8 bipolar derivations	Silver/AgCl; analyses focused on C4–O2 and C3–O1	delta1 as 0.7–2 Hz, delta2 as 2–4 Hz, theta as 4–8 Hz, alpha as 8–12 Hz, beta1 as 12–18 Hz, and beta2 as 18–30 Hz	Increased alpha/beta and reduced theta/delta; beta/delta changes more persistent in caffeine-sensitive group	Between-group delta/beta difference at 5 h, *p* < 0.05; other time points *p* > 0.05	Caffeine-related insomnia/prolonged sleep latency in sensitive participants
[47]	Two controlled repeated-measures crossover trials	United Kingdom	Trial 1 *n* = 12; Trial 2 *n* = 12; one participant in both; EEG mainly Trial 2 *n* = 12	Trial 1: 21–43 y, 9 F/3 M; Trial 2: 21–32 y, 5 F/7 M	Healthy volunteers		No caffeine-containing beverages on pre-trial day; none before testing on experimental day	Anhydrous caffeine in gelatin capsules; Trial 1: 75, 150, 300 mg; Trial 2: 100 mg	Lactose dummy; cyclizine conditions also included	Balanced Latin-square; double-blind	2.8 and 6.1 h	Relaxed eyes closed	2	Fz and Pz; Jasper system; indifferent mastoid electrode	delta, 2.3–4.0 Hz; theta, 4.0–7.5 Hz; alpha, 7.5–13.5 Hz; and beta, 13.5–26 Hz	At 2.8 h, 100 mg caffeine increased delta-band energy; alpha/beta not significantly different from lactose	Delta 57.0 vs. 54.1 arbitrary units (caffeine vs. lactose), *p* < 0.05; no significant difference by 6.1 h	Auditory vigilance, reaction time and tapping improved in Trial 1 at some doses; Trial 2 100 mg showed no significant vigilance/reaction-time benefit
[48]	Randomized, double-blind, placebo-controlled crossover	United Kingdom	9/9	18–40 y; 4 male, 5 female	Healthy adults	428 ± 146 mg/day (230–670)	24 h	Anhydrous caffeine in matching capsules; 250 mg and 500 mg	Placebo capsule	Randomized; double-blind	1, 3 and 5 h	Eyes open during auditory reaction-time task	2	Cz and T3	theta, defined as 4–7.5 Hz; alpha, defined as 8.0–13.5 Hz; and beta, defined as 14–32 Hz	Dose-related decreases in theta and alpha log power; beta showed a paradoxical dose pattern	0.05 critical differences: theta 0.268; alpha 0.264; beta 0.635; total power 0.217	No significant caffeine–placebo differences in tapping, reaction time, DSST, symbol copying or cancellation; alertness increased subjectively
[49]	Randomized, double-blind, placebo-controlled repeated-measures crossover	United States	12/12	21–32 y; 12 male	Healthy men		48 h instructed in one caffeine and one placebo session; poor adherence	Two caffeine citrate capsules; 200 mg caffeine citrate total	Two placebo capsules	Randomized; double-blind	30 min	Eyes closed; 2-min resting EEG before each photic-stimulation period	4 recorded; O1/O2 analyzed	O1, O2, C3, C4; Cz reference; International 10–20	analyzed narrow 1-Hz frequency bands centered on the frequencies used for sine-wave-modulated photic stimulation. The fundamental-frequency bands were 4.5–5.5 Hz, 9.5–10.5 Hz, 14.5–15.5 Hz, 17.5–18.5 Hz, and 23.5–24.5 Hz,	Reduced power at 15 Hz and reduced resting/photic responses at 10 Hz; reduced photic response at 24 Hz	15 Hz F(1,11) = 7.35; 10 Hz three-way interaction F(3,33) = 2.81; 24 Hz drug × stimulus interaction F(3,33) = 3.21	
[50]	Within-subject pre/post 2 × 4 crossover	Switzerland	20/20	Mean 33.4 ± 7.0 y (23–44); 20 female	Healthy nonsmoking regular coffee drinkers	6.4 ± 3.5 cups/day (3–16)	12 h	Anhydrous caffeine in 150 mL decaffeinated coffee; 1.5, 3.0, 6.0 mg/kg	Decaffeinated coffee, 0 mg/kg caffeine	Order fully balanced	~30 min	5-min resting phases and 20-min rapid information processing task	1	Cz; International 10–20; combined ears reference; mid-forehead ground	delta, 1.0–3.9 Hz; theta, 4.0–7.8 Hz; alpha, 7.9–11.7 Hz; and beta, 11.8–24.7 Hz	No significant band-power effect; increased dominant alpha and beta frequency at higher doses	Dominant alpha frequency F(3,50) = 6.30, *p* < 0.01; dominant beta frequency F(3,53) = 5.44, *p* < 0.01	RIP processing rate improved at 1.5 and 3.0 mg/kg; reaction time decreased at 1.5 and 6.0 mg/kg
[51]	Randomized, double-blind, placebo-controlled repeated-measures crossover	Australia	18/18	Mean 21 y (17–36); 13 female, 5 male	Healthy nonsmoking undergraduates	Moderate; ~2–4 cups coffee/cola equivalents/day	≥12 h	Gelatin capsule; 250 mg	Placebo capsule	Randomized; double-blind	Mean 32.0 min (29–35)	2-min resting eyes closed	17	International 10–20; linked ears; ground between Fpz and Fz	delta, 1.5–3.5 Hz; theta, 4–7.5 Hz; alpha, 8–13 Hz; and beta, 13.5–25 Hz	Reduced global alpha power and increased mean alpha frequency	Alpha 21.3 vs. 16.2 μV^2^, F(1,17) = 7.60, *p* < 0.05; mean alpha frequency 10.16 vs. 10.30 Hz, F(1,17) = 4.44, *p* < 0.05	
[52]	Randomized, double-blind, placebo-controlled repeated-measures crossover	Australia	24/24	Mean 21.5 y (17–36); 17 female, 7 male	Healthy nonsmoking university students	Moderate; ~2–4 cups coffee/day	≥4 h	Gelatine capsule; 250 mg	Identical placebo capsule	Randomized; double-blind	Recording started mean 3.9 ± 3.7 min; later block mean 52.3 ± 7.3 min	Alternating 2-min eyes closed/eyes open; only eyes-closed epochs analyzed; intervening oddball/CPT	19	International 10–20; linked ears; ground between Fpz and Fz	delta, 1.5–3.5 Hz; theta, 4–7.5 Hz; alpha, 8–13 Hz; and beta, 13.5–25 Hz	Reduced global alpha power; beta also reduced; no significant global delta/theta effect	At 33 min alpha 32.9 µV^2^ (SD 35.2) vs. 45.2 µV^2^ (SD 52.8), F(1,23) = 3.81, one-tailed *p* < 0.05; standardized alpha F = 3.45, *p* = 0.038; beta F = 4.75, *p* = 0.020	
[53]	Randomized, double-blind crossover	South Korea	11/11	Mean 31 ± 9 y; 5 female, 6 male	Healthy young adults		24 h	Caffeinated coffee; 3 mg/kg; mean 181 ± 34 mg	Decaffeinated coffee	Randomized; double-blind	30–46 min	2 min EC, 2 min EO, 10 min EC, 2 min EO; QEEG based on two 5-min EC segments	19 derived EEG channels	21 gold-disk electrodes; International 10–20; C3/C4 physical references; derived data averaged-ears reference	Delta1 was defined as 0.5–2.0 Hz, Delta2 as 2.5–4.0 Hz, Theta1 as 4.5–6.0 Hz, Theta2 as 6.5–8.0 Hz, Alpha1 as 8.5–11.0 Hz, Alpha2 as 11.5–14.0 Hz, Beta as 14.5–25.0 Hz, and Gamma as 25.5–50.0 Hz.	Lower Delta2/Theta1 absolute power; relative Delta2/Theta1 lower, Beta and Alpha2 higher depending on segment	Topographic differences *p* < 0.05 or *p* < 0.01; dissimilarity index absolute 0.057→0.102 and relative 0.057→0.100	
[54]	Randomized, double-blind, placebo-controlled parallel-group study after sleep deprivation	Brazil	10/10	25–35 y; mean 30.4 ± 5.2; 5 male, 5 female	Healthy, right-handed, nonsmoking adults	Moderate; 1–2 cups coffee/day	10 h	Capsule; 400 mg	Placebo capsule	Randomized; double-blind	1 h	5-min resting eyes closed	20	International 10–20; monopolar; linked earlobes; ground Fpz	delta (1.0–3.5 Hz), theta (4.0–7.5 Hz), alpha (8.0–12.0 Hz), and beta (12.5–25 Hz)	Reduced alpha absolute/relative power and theta relative power after caffeine	Alpha absolute interaction F = 4.20, *p* = 0.023; relative delta F = 5.02, *p* = 0.012; theta F = 13.78, *p* < 0.001; alpha F = 4.20, *p* = 0.023	Reaction time decreased numerically after caffeine but no treatment × moment interaction; Stroop and Digit Span nonsignificant
[55]	Randomized, double-blind, placebo-controlled crossover	United States	14/14 main analyses; 13 peak-frequency analysis	Panic disorder: mean 36.0 ± 6.2 y, 2 M/5 F; controls: mean 32.1 ± 5.0 y, 3 M/4 F	7 panic-disorder patients; 7 healthy controls		14 days	Liquid caffeine base; 7 mg/kg	Identical-tasting placebo	Randomized; double-blind	30–45 min and 75–90 min	15-min resting eyes closed	28	International 10–20 plus Oz, FC5/6, CP5/6, CP1/2, PO1/2; common-average reference	delta as 0.4–5.9 Hz, theta as 6.3–8.2 Hz, slow alpha as 8.6–10.5 Hz, fast alpha as 10.9–12.5 Hz, slow beta as 12.8–18.4 Hz, medium beta as 18.8–21.0 Hz, and fast beta as 21.4–30.0 Hz. In addition, the background peak frequency was determined in the occipital region within the 7.8–13.0 Hz range.	Increased occipital peak alpha frequency; reduced slow-alpha and central slow-beta amplitude	Peak alpha drug × time F = 7.71, *df* = 2.22, *p* < 0.01; +0.32 Hz at ~30 min and +0.40 Hz at ~75 min. Slow-alpha interaction F = 6.49, *p* < 0.01; slow-beta F = 4.22, *p* < 0.05	Anxiety increased; Zung drug × time F = 27.05, *p* < 0.0005; greater response in panic disorder
[56]	Randomized, double-blind, placebo-controlled repeated-measures crossover	Australia	30/30	8–13 y; mean 10.5; 11 female, 19 male	Healthy children	Light-to-moderate; ~1–2 cans caffeinated-cola equivalents/week	≥4 h	Gelatin capsule; 80 mg; ~1.3–3.3 mg/kg	Placebo capsule	Randomized; double-blind	Mean 31.5 min (25–46)	3-min resting eyes closed	19	International 10–20; linked ears; ground between Fpz and Fz	delta: 1.5–3.5 Hz, theta: 4–7.5 Hz, alpha: 8–13 Hz, and beta: 13.5–25 Hz	Reduced delta, theta, alpha and beta power; increased mean alpha frequency	Delta F(1,29) = 3.40, *p* < 0.05; theta F = 2.96, *p* < 0.05; alpha F = 8.78, *p* < 0.005; beta F = 9.23, *p* < 0.005; alpha frequency 9.74→9.81 Hz, F = 3.17, *p* < 0.05	Skin conductance increased 9.2 ± 3.3 to 10.7 ± 4.7 μS, F(1,29) = 4.04, *p* < 0.05
[57]	2 × 2 caffeine × smoking pre-post crossover	Switzerland	20 total; behavioral *n* = 20; EEG ~18–19	Mean 28.3 y (21–39); all female	Healthy women; regular smokers and coffee drinkers	5.4 cups/day (3–13)	From 20:00 evening before session	250 mg caffeine in 150 mL decaffeinated coffee	Decaffeinated coffee without added caffeine	Treatment order balanced	Analyzed rest began >20 min post-dose	5-min resting period	3	Cz, P3, P4; International 10–20; combined ears reference; mid-forehead ground	delta (δ): 0–4 Hz, theta (θ): 4–8 Hz, alpha (α): 8–12 Hz, and beta (β): 12–25 Hz.	Decreased theta and increased beta power at parietal sites	Theta: P3 F(1,18) = 9.70, *p* < 0.01; P4 F = 6.43, *p* < 0.05. Beta: P3 F = 31.70, *p* < 0.001; P4 F = 9.36, *p* < 0.01	Numerical Stroop: shorter reaction times and reduced interference
[58]	Single-centre randomized, double-blind, placebo-controlled single-dose crossover during sleep deprivation	France	12/12	19–27 y; 12 male	Healthy young men	Low-to-moderate; <5 cups/glasses caffeinated beverages/day	24 h	Slow-release caffeine; two 300-mg capsules; 600 mg total	Placebo	Randomized; double-blind	~4, 9, 14 and 19 h	5-min resting eyes closed	4 leads	F4–T4, F3–T3, T4–O2, T3–O1; vertex reference	delta, 0–4 Hz; theta, 4–8 Hz; alpha, 8–12 Hz; and beta, 12–40 Hz, with the beta range further subdivided into 12–16 Hz, 16–20 Hz, 20–30 Hz, and 30–40 Hz.	Decreased relative delta/theta; increased alpha and beta relative power	Beta increase ~100–200%, peaking ~4 h; generally *p* < 0.05; figures mean ± SEM	Improved choice-reaction/Stroop times, CPT correct responses and body sway; reduced subjective sedation
[59]	Double-blind, placebo-controlled within-subject crossover with maintenance/withdrawal conditions	Ireland	22/22	17–44 y; mean 19; 5 male, 17 female	Healthy university volunteers	159–665 mg/day; mean 277 mg/day (~2–4 cups coffee)	Six-day placebo/caffeine maintenance phases; all other caffeine avoided throughout 4 weeks	Gelatin capsules; 1.75 mg/kg per dose, three times daily	Identical maize-starch capsule	Double-blind; condition order counterbalanced	~50 min after day-7 challenge dose	5 min eyes closed, 5 min eyes open, and two 15-min SART tasks	32	International 10–20; Ag/AgCl; average of all connected inputs	delta band as 0.5–3.4 Hz, theta as 3.5–7.5 Hz, alpha as 7.6–12.0 Hz, and beta as 12.1–30.0 Hz	Modest theta/alpha effects; no delta or beta effects	Theta left frontal F(3,63) = 5.29, *p* < 0.05; theta condition interaction F(6126) = 2.45, *p* < 0.05; alpha left frontal F = 3.20, *p* < 0.05; right central F = 4.74, *p* < 0.01; partial η^2^ 0.01–0.18	SART: no significant caffeine effect, F(3,63) = 2.43, *p* > 0.05, partial η^2^ = 0.10
[60]	Comparative within-subject pre–post pharmacological EEG study	Russia	25/25	18–28 y; men and women				Oral caffeine sodium benzoate; 100 mg	Pre-drug baseline; phenazepam 1 mg active comparison		50–65 min	5–7 min quiet wakefulness, eyes closed	19	International 10–20; monopolar Ag/AgCl; linked earlobes	delta (δ), 1.5–4 Hz; theta (θ), 4–7 Hz; lower alpha (α1), 7–10 Hz; upper alpha (α2), 10–13 Hz; lower beta (β1), 13–18 Hz; upper beta (β2), 18–30 Hz; and gamma (γ), 30–40 Hz.	Reduced integrated/parieto-occipital power and increased coherence; band-specific beta2/gamma increases and alpha1/theta/delta reductions	ANOVA with Greenhouse–Geisser correction and Fisher LSD; *p*-values reported	
[61]	Repeated-measures within-subject caffeine × nicotine factorial study	United States	12/12	19–31 y; mean 22.75 ± 3.72; 12 male		86.25–390 mg/day; mean 225.5 ± 82.69	From midnight before testing	Anhydrous caffeine in caffeine-free cola; two 150-mg doses 90 min apart; 300 mg total	Caffeine-free cola with 3 mg quinine; nicotine conditions crossed	Counterbalanced; double-blind except own-cigarette condition	~49 min after dose 1; ~19 and ~49 min after dose 2	3 min eyes open then 3 min eyes closed	19	International 10–20; nose-tip reference; Fpz ground	delta (1.5–4.0 Hz), theta (4.0–8.0 Hz), alpha1 (8.0–10.5 Hz), alpha2 (10.5–13.0 Hz), beta1 (13.0–20.0 Hz), and beta2 (20.0–30.0 Hz).	Reduced delta, theta, alpha1 and beta1 power	Delta F(1,11) = 8.00, *p* = 0.016, η^2^ = 0.42; theta F = 24.26, *p* < 0.001, η^2^ = 0.69; alpha1 F = 20.24, *p* < 0.001, η^2^ = 0.65; beta1 F = 54.36, *p* < 0.001, η^2^ = 0.83	Visual rapid information processing: target detections increased, F(1,11) = 11.43, *p* = 0.006, η^2^ = 0.54
[62]	Randomized, double-blind, placebo-controlled crossover	Netherlands	40 recruited/38 analyzed	18–25 y; mean 21.90 ± 2.05; all female	Healthy participants	Low; ≤100 mg/day; mean ~53 mg/day	12 h	Oral capsule; 200 mg	Indistinguishable filler-only capsule	Randomized; counterbalanced; double-blind	30 min	8-min resting EEG alternating 1-min eyes open/eyes closed	3	F3, Fz, F4; International 10–20; offline left/right mastoid reference	theta = 4–7 Hz and beta = 13–30 Hz	Theta/beta ratio unchanged; theta and beta power reduced	TBR F(1,37) = 0.130, *p* = 0.721, partial η^2^ = 0.003; theta F = 20.526, *p* < 0.001, partial η^2^ = 0.357; beta F = 48.297, *p* < 0.001, partial η^2^ = 0.566	Pictorial Emotional Stroop interference: no overall caffeine effect; moderation by baseline TBR and trait anxiety
[63]	Two double-blind counterbalanced crossover driving experiments	United Kingdom	16/16 (8 per experiment)	Mean 23 ± 2 SE; 8 male, 8 female	Healthy medication-free graduate students/experienced drivers	Moderate; 2–4 cups caffeinated coffee/day	~11.5 h	200 mg anhydrous caffeine in 200 mL decaffeinated coffee + milk	Same drink without added caffeine	Double-blind; counterbalanced	~25–30 min	Simulated monotonous driving task	1	C3–A1; EOG channels referenced to A2	theta, 4–7 Hz, and alpha, 8–11 Hz	No overall EEG effect; lower 4–11 Hz theta + alpha power during part of Study 1	Study 1 s 30-min period t(7) = 1.99, one-tailed *p* < 0.04; Study 2 no significant EEG effect	Sleep-related driving incidents/lane drifting reduced, especially after 5 h sleep restriction; subjective sleepiness reduced
[64]	Double-blind, placebo-controlled crossover during prolonged wakefulness	France	37 analyzed overall/34 EEG	18–55 y; mean 30.2 ± 4.4; 15 female, 22 male	Healthy; no medical, psychiatric or sleep disorders	Mean 212 ± 66 mg/day; low 0–50, moderate 51–300, high >300 mg/day	No pre-experimental abstinence	Caffeine powder in decaffeinated beverage; 2.5 mg/kg per administration, twice daily (5 mg/kg/day)	Placebo in decaffeinated beverage	Double-blind	45 min	10-min psychomotor vigilance task	19	International 10–20; bridged mastoids initially, common-average re-reference	theta = 4–8 Hz, alpha = 8–12 Hz, and beta = 12–20 Hz.			PVT reaction time shortened: t = −5.12, Holm-adjusted *p* < 0.001; acute-treatment F = 26.72, *p* = 0.001
[65]	Randomized, counterbalanced, single-blind within-subject placebo-controlled dose-response study	Australia	10/10 participants			Regular users; ≥1 cup coffee/tea/day	12 h	No Doze tablets; 100-mg increments to cumulative 100–600 mg	25-mg vitamin B-complex tablets	Randomized; counterbalanced; single-blind	Trials separated by 15 min	Four cognitive tasks; 10-s eyes-closed relaxation between tasks	4	Fz, F3, F4, Cz; International 10–20	delta: 0.5–4 Hz, theta: 4.5–7.5 Hz, alpha: 8–12 Hz, and beta: 12.5–30 Hz	Correlation dimension (D2) showed a nonlinear quadratic dose-response	Treatment × session × task F(21,245.3) = 3.65, *p* = 0.001; quadratic + linear R^2^ = 0.12–0.74 across caffeine task conditions	
[66]	Randomized, double-blind, placebo-controlled crossover	France	10/10	Mean 27.2 y; 6 male, 4 female	Healthy, right-handed; no medical treatment		Avoided tea, coffee, chocolate and cola on day before session	Oral caffeine; 400 mg	Placebo	Randomized; double-blind	1 h	2.5 min resting eyes closed followed by 2.5 min active wakefulness eyes open	16	Bihemispheric symmetrical Giannitrapani montage; common-average reference; silver electrodes	delta, 1.56–3.52 Hz; theta, 3.91–7.42 Hz; alpha, 7.81–14.06 Hz; beta 1, 14.45–28.13 Hz; and beta 2, 28.52–49.61 Hz.	Increased mean alpha frequency; decreased alpha amplitude, relative alpha, theta amplitude and total RMS amplitude	Alpha frequency up to +0.34 Hz (11/16 channels); alpha amplitude down ~13 µV (11/16); relative alpha down up to 5% (7/16); theta down up to 4 µV (6/16); *p* ≤ 0.05 or *p* ≤ 0.01	
[67]	Double-blind, placebo-controlled crossover	Netherlands	16/16	19–29 y; mean 21.5 ± 2.9; 8 female, 8 male	Healthy, right-handed undergraduates; nonsmokers	5.9 ± 2.6 cups caffeinated coffee/day	≥12 h	Caffeine dissolved in decaffeinated coffee; 3 mg/kg	3 mg/kg lactose in decaffeinated coffee	Double-blind; treatment order balanced	>45 min	1-min eyes-open background EEG plus visual selective-attention task	5 derivations	Fz, Cz, Pz, Oz to linked earlobes + bipolar C3–C4; International 10–20	The delta (δ) band was defined as 0–3.1 Hz, theta (θ) as approximately 3.2–6.2 Hz, and the alpha range was subdivided into lower alpha (6.3–9.4 Hz) and higher alpha (9.4–12.5 Hz). Frequencies above the conventional alpha range included a sigma (σ) range around 12.5–15.6 Hz, while the beta (β) range was defined as approximately 15.7–25 Hz. In addition to band power, the investigators separately determined alpha peak frequency, defined as the frequency showing the greatest power within 7.8–13.6 Hz.	Reduced background EEG power, largest in lower-alpha range	Significant effects: 0–3.1 Hz F = 10.4; 6.3–15.6 Hz F ≈ 7.4–9.0; 15.7–25 Hz F ≈ 5.7–12.5; 3.2–6.2 Hz F = 0.6	Target RT 404.6 ± 29.7 to 382.9 ± 33.2 ms; −21.7 ms, F = 34.6, *p* < 0.0001; hit rate higher, F = 12.1, *p* < 0.005
[68]	Randomized, double-blind, placebo-controlled repeated-measures crossover	Canada	Total reported inconsistently: *n* = 25 (Methods) vs. *n* = 27 (Table 1)	Controls: 19–35 y, 23.15 ± 4.18, 9 M/4 F; patients: 23–38 y, 28.17 ± 3.59, Methods 9 M/3 F, Table 11 M/3 F	Clinically stable early-phase schizophrenia/psychosis plus healthy controls	Habitual use required; CCQ 1490.0 ± 1201.4 psychosis vs. 1250.1 ± 1398.8 controls	From midnight until morning session	Caffeine pill with water; 200 mg	Physically identical placebo pill	Double-blind; order counterbalanced	30 min	3-min eyes open then 3-min eyes closed	64	10–10; ActiCAP/ActiCHamp; mastoid online reference		Microstate D duration/contribution decreased and A/B occurrence increased in psychosis group	Microstate D duration g = 1.00, *p* = 0.014; A occurrence g = 0.78, *p* = 0.017; B occurrence g = 1.07, *p* = 0.001; D contribution g = 1.40, *p* = 0.001	
[69]	Balanced repeated-measures within-subject crossover	United States	16/16	21–40 y; mean 27.5; 5 female, 11 male	Neurologically normal volunteers	3.7 cups tea/week + 3.8 cups coffee/week	≥24 h	50 mg caffeine in 100 mL water; also caffeine + 100 mg L-theanine condition	Water placebo; L-theanine-only condition also included	Fully counterbalanced; participants blinded	30 min	Cued visuospatial attention task	164	164 scalp electrodes; offline nasion reference; alpha analyses used selected electrode subsets	alpha frequency band, defined as 8–14 Hz	Caffeine alone reduced the alpha cueing effect; tonic alpha was not significantly reduced by caffeine alone	Alpha cueing effect *p* < 0.02; caffeine-alone tonic alpha *p* = 0.18; combined treatment tonic alpha *p* < 0.02	Caffeine alone improved d′, *p* < 0.016, d = 0.42; combined treatment improved hit rate and d′
[70]	Double-blind within-subject repeated-measures caffeine × nicotine study	United States	6/6	25–32 y; mean 28.2; 6 male	Healthy regular smokers/coffee drinkers; nicotine-dependent	3–10 cups coffee/day; mean 5.1	12 h	Anhydrous caffeine in 3 gelatin capsules with ~240 mL water; 150 or 300 mg	Lactose placebo; nicotine gum conditions and nondeprived control also included	Double-blind; order balanced	Baseline; 25, 50, 125, 150 min	1-min spontaneous eyes closed	4	Fz, Pz, C3, C4; linked ears	delta, 0.25–3.75 Hz; theta, 4–7 Hz; alpha, 7.25–14 Hz; and beta, 14.25–25 Hz.	Decreased frontal theta and prevented deprivation-related alpha-frequency decrease	Theta F(2,10) = 8.21, *p* < 0.01; alpha frequency F(2,10) = 4.23, *p* < 0.05	Deprivation slowed column addition and reduced digit recall; nicotine × caffeine × time interaction for digit recall F(4,20) = 3.43, *p* < 0.05
[71]	Randomized, double-blind, placebo-controlled triple-crossover caffeine challenge	United Kingdom	36/36 (replacement maintained 12/group)	Mean age: controls 34, GAD 36, PD 35; M:F 6:6, 9:3, 7:5	12 GAD, 12 panic disorder, 12 healthy controls	Mean 360 mg/day controls; 330 mg/day GAD; 490 mg/day PD	1 week	Matching white capsules; anhydrous caffeine 250 mg or 500 mg	Placebo	Randomized; balanced order; double-blind	1, 3 and 5 h	Eyes-open alpha activity			delta was defined as 2.5–4 Hz, theta as 4.5–7.5 Hz, alpha as 8–13 Hz, and beta as 13.5–26 Hz.	Smaller caffeine-related alpha decrease in GAD and PD versus controls; dose-related alpha/beta decreases across groups	GAD vs. controls F(1108) = 4.38, *p* < 0.05; PD vs. controls F(1108) = 5.13, *p* < 0.05	Impairment on DSST and four-letter cancellation; increased self-rated anxiety
[71]	Placebo-controlled between-subject experimental study	Switzerland	40/40	Mean 33.7 y caffeine group, 35.1 y placebo group; all female		~5 cups/day (5.0 caffeine group; 5.1 placebo)	12 h	Caffeine in decaffeinated coffee; 3.3 mg/kg	Decaffeinated coffee without added caffeine		30 min	5-min rest, 20-min RIP stress-coping task, 5-min rest	1	Cz; International 10–20; combined ears reference; mid-forehead ground	delta (1.0–3.9 Hz), theta (4.0–7.8 Hz), alpha (7.9–11.7 Hz), and beta (11.8–24.7 Hz).	Increased dominant alpha/beta frequency and delta power; decreased beta power	Alpha frequency F(1,31) = 5.27, *p* < 0.05; beta frequency F(1,30) = 5.82, *p* < 0.05; delta F(1,31) = 8.26, *p* < 0.01; beta F(1,32) = 4.86, *p* < 0.05	RIP reaction time reduction greater during active than passive coping, F(1,31) = 7.67, *p* < 0.01; no general cognitive improvement
[72]	Within-subject repeated-measures crossover	Australia	30/30 EEG; actigraphy *n* = 28	18–25 y; mean 23.2 ± 2.22; 20 female, 10 male	Healthy nonprofessional drivers	Neither caffeine avoiders nor heavy caffeine users	At least one night	Stay Alert chewing gum; 100 mg	Taste-matched decaffeinated gum; light-only and caffeine + light conditions also included	Randomized; counterbalanced	Gum ~15 min before driving session	30-min simulated-driving task; eyes-open task condition	2	C4–A1 and O1–A2; International 10–20; mastoid references	alpha = 8–13 Hz and theta = 4–8 Hz.	Caffeine alone did not significantly change alpha or theta power; caffeine + light reduced alpha	Caffeine alone: alpha β = 0.008, 95% CI −0.003 to 0.019, *p* = 0.137; theta β = 0.007, 95% CI −0.012 to 0.027, *p* = 0.477	Lateral lane SD reduced by caffeine: β = −0.123 m, 95% CI −0.153 to −0.092, *p* < 0.0001
[73]	Randomized, double-blind, placebo-controlled repeated-measures crossover	United States	12 enrolled/10 analyzed	24–33 y; mean 25.6; 4 male, 6 female	Healthy volunteers	<50 mg/day during previous 2 months		Oral capsule; 200 mg	Identical cornstarch capsule	Randomized; double-blind	~40 min	Two 5-min simultaneous EEG-fMRI rest recordings: eyes closed and eyes open	64	MR-compatible 10–20 system; FCz reference; AFz ground	delta (1–4 Hz), theta (4–7 Hz), alpha (7–13 Hz), and beta (13–30 Hz)	Caffeine-related EEG vigilance changes strongly covaried with global fMRI signal changes in eyes-closed condition	Global fMRI change correlations: delta R = 0.92, *p* = 0.0002; theta R = 0.77, *p* = 0.01; alpha R = −0.82, *p* = 0.004; vigilance R = −0.91, *p* = 0.0002	
[74]	Randomized, double-blind, single-dose three-way crossover	United States	12/12	Table: 22–41 y, 5 male, 7 female; Methods recruited 20–46 y	Healthy ambulatory volunteers; no medical disease/medications	10/12 regular caffeine users	24 h	Opaque capsules; 250 mg and 500 mg caffeine	Placebo capsule	Randomized; double-blind; EEG segments reviewed by blinded observer	0.5, 1, 1.5, 2, 2.5, 3, 4, 5, 6 and 8 h		4	Fz, Cz, Pz, Oz; nose reference	alpha band as 8–11 Hz and the beta band as 12–30 Hz	Both doses generally reduced total/alpha/beta EEG amplitude, mainly central/parietal	Cz beta 4-h effect area: 8.75 placebo, −3.69 (250 mg), 6.03 (500 mg), F = 10.02, *p* < 0.005; Pz total 22.94, −10.84, −13.31, F = 11.64, *p* < 0.002	DSST and tapping speed: 250 mg improved performance at several time points; 500 mg less enhancement
[75]	Randomized, double-blind, placebo-controlled counterbalanced crossover	United States	16/16	21–32 y; mean 26; 8 female, 8 male	Healthy nonsmoking adults	Moderate; 1–4 cups coffee/day		Unmarked gelatin capsule; 200 mg	Powdered-sugar placebo capsule	Randomized; double-blind; counterbalanced	Recording periods began 0.5, 1.5, 2.5 and 3.5 h	Low/high-load n-back task plus quiet rest eyes open/eyes closed	28	Digitally linked mastoids; spectral analyses focused on AFz, Pz, Oz	individual peak frequency occurring between 8 and 12 Hz at Pz. During resting EEG, delta was defined as 2–4 Hz and posterior theta as 4–6 Hz, both measured at Pz. Resting occipital alpha was measured at Oz as the average power within a 1-Hz band centered on the peak frequency within the 8–12 Hz range	No resting EEG change; reduced parietal alpha during working-memory task	Task alpha F(4,60) = 4.91, *p* < 0.01; multivariate discrimination *p* < 0.01 at 0.5–1 h, peak *p* < 0.001 at 1.5–2 h	No significant change in working-memory reaction time or accuracy
[76]	Randomized, double-blind, placebo-controlled crossover during 40-h wakefulness	Switzerland	23 enrolled/22 analyzed	Sensitive 24.1 ± 0.9 y; insensitive 25.5 ± 0.7 y; all male	Healthy young men; caffeine-sensitive vs. insensitive	58.3 ± 24.4 mg/day sensitive; 96.0 ± 27.1 mg/day insensitive	2 weeks	Capsules; 200 mg at 11 and 23 h wakefulness; 400 mg total	Placebo capsules	Randomized; double-blind	Waking EEG every 3 h; post-dose intervals embedded in protocol	3 min eyes closed then 5 min eyes open; analyses based on eyes open	4 bipolar derivations	F3–C3, F4–C4, P3–O1, P4–O2; sleep staging C3–A2	delta activity at 0.25–1.75 Hz, alpha at 7.75–11.75 Hz, and beta at 12.25–17.75 Hz; most importantly, they operationally defined theta power as 6.25–8.25 Hz for their detailed analyses of sleep deprivation and caffeine effects.	Reduced waking theta power, particularly in caffeine-sensitive participants	Main caffeine F(1,20) = 22.6, *p* < 0.001; caffeine × derivation × sensitivity F(1,20) = 6.3, *p* < 0.03; sensitive regional effect F(1,11) = 11.0, *p* < 0.007	PVT speed improved, especially in sensitive participants; improvement correlated inversely with sleep-deprivation impairment, r ≈ −0.67 to −0.69, *p* < 0.001
[77]	Randomized, double-blind, counterbalanced within-participant crossover	United States	27 recruited/21 analyzed	18–40 y; mean 26; 8 female	Medically healthy adults	Very low: 14/21 no daily coffee/tea; remaining mostly 1 cup/day; two participants 2–3 cups coffee/day	≥24 h	Caffeine solution in 200 mL water; 50 mg; also 50 mg caffeine + 100 mg L-theanine	200 mL water placebo; L-theanine-only condition also included	Randomized; counterbalanced; double-blind	~25–185 min	Visual Go/No-Go SART task	168 acquired; 160 analyzed	Custom BioSemi ActiveTwo scalp array; nose reference	Alpha 8–14 Hz	Decreased alpha activity, with effect diminishing after ~1 h	Initial alpha reduction ~12%; caffeine main effect F(1,57.0) = 7.5, *p* < 0.01; caffeine × time F(1250.9) = 9.9, *p* < 0.01	Omission errors −50%, commission errors −30% (both *p* < 0.0001); reaction time ~3% faster, *p* < 0.05

AEP, auditory evoked potential; ANOVA, analysis of variance; CCQ, Caffeine Consumption Questionnaire; CI, confidence interval; CNV, contingent negative variation; CPT, Continuous Performance Test; DSST, Digit Symbol Substitution Test; EC, eyes closed; EEG, electroencephalography; EEG–fMRI, simultaneous electroencephalography and functional magnetic resonance imaging; EOG, electrooculography; EO, eyes open; fMRI, functional magnetic resonance imaging; GAD, generalized anxiety disorder; HC, high habitual caffeine-consumption group; KLT, *Konzentrationsleistungstest* (concentration-performance test); LC, low habitual caffeine-consumption group; LSD, least significant difference; M, male; F, female; MoCA, Montreal Cognitive Assessment; MR, magnetic resonance; PD, panic disorder; PVT, Psychomotor Vigilance Task; QEEG, quantitative electroencephalography; RIP, Rapid Information Processing task; RMS, root mean square; RT, reaction time; SART, Sustained Attention to Response Task; SD, standard deviation; SE, standard error; SEM, standard error of the mean; TBR, theta/beta ratio; TMT-A, Trail Making Test Part A; TMT-B, Trail Making Test Part B; D2, correlation dimension; P300, positive event-related potential component occurring approximately 300 ms after stimulus presentation; d′, sensitivity/discriminability index; F, F statistic; t, t statistic; *p*, *p*-value; r/R, correlation coefficient; d, Cohen’s d; g, Hedges’ g; η^2^, eta-squared; partial η^2^, partial eta-squared; R^2^, coefficient of determination; df, degrees of freedom; y, years; h, hours; min, minutes; s, seconds; ms, milliseconds; mg, milligrams; kg, kilograms; mL, milliliters; Hz, hertz; dB, decibels; µV, microvolts; µV^2^, squared microvolts; µS, microsiemens.

**Table 2:** Risk of bias of included studies (RoB2).

Study	Bias Arising from the Randomization Process	Bias Due to Deviations from Intended Interventions	Bias Due to Missing Outcome Data	Bias in Measurement of the Outcome	Bias in Selection of the Reported Result	Overall Judgment
[31]	Some concerns	Low risk	Low risk	Low risk	Some concerns	Some concerns
[33]	Some concerns	Low risk/Some concerns	Low risk	Low risk for EEG/P300/TSAT; some concerns for RPE	Some concerns	Some concerns
[34]	Some concerns	Low risk	Low risk	Some concerns	Some concerns	Some concerns
[35]	Some concerns	Low risk	Some concerns	Low risk	Some concerns	Some concerns
[36]	Low risk	Low risk	Low risk/Some concerns	Low risk	Some concerns	Some concerns
[37]	Some concerns	Some concerns	Low risk/Some concerns	Low risk	Some concerns	Some concerns
[38]	Some concerns	Low risk	Low risk/Some concerns	Low risk	Some concerns	Some concerns
[41]	Some concerns	Low risk	Low risk	Low risk	Some concerns	Some concerns
[42]	Some concerns	Low risk	Low risk/Some concerns	Low risk	Some concerns	Some concerns
[43]	Some concerns	Some concerns	Low risk	Some concerns	Some concerns to high risk	High risk
[44]	Some concerns	Some concerns	Low risk	Some concerns	Some concerns	Some concerns
[47]	Some concerns	Low risk	Some concerns/High for Trial 1 EEG	Some concerns	Some concerns	High risk
[48]	Some concerns	Low risk for EEG outcomes	Low risk	Low risk/some concerns	Some concerns	Some concerns
[49]	Some concerns	Some concerns	Some concerns	Low risk/Some concerns	Some concerns	Some concerns
[50]	Some concerns	Some concerns	Low risk	Low risk for EEG; some concerns for subjective/performance outcomes	Some concerns	Some concerns
[51]	Some concerns	Low risk	Some concerns	Low risk	Some concerns	Some concerns
[52]	Some concerns	Low risk	Low risk/Some concerns	Low risk	Some concerns	Some concerns
[53]	Some concerns	Low risk/Some concerns	Some concerns	Some concerns	High risk/Some concerns, leaning high	High risk
[54]	Some concerns/high risk	Some concerns	Low risk/some concerns	Low risk for qEEG/ERP; some concerns for mood outcomes	Some concerns/high concerns	High risk
[55]	Some concerns	Low risk/Some concerns	Some concerns	Low risk/some concerns	High risk	High risk
[56]	Some concerns	Low risk	Low risk	Low risk	Some concerns	Some concerns
[57]	Some concerns	Some concerns	Some concerns	Low risk for EEG/RT; some concerns for subjective ratings	High risk	High risk
[58]	Some concerns	Low risk/Some concerns	Low risk	Low risk for EEG; Some concerns for subjective outcomes	Some concerns	Some concerns
[59]	Some concerns	Low risk	Low risk/some concerns	Low risk	Some concerns	Some concerns
[61]	Some concerns	Some concerns	Low risk	Some concerns	Some concerns/high risk for exploratory EEG findings	High risk
[62]	Low risk	Some concerns	Low risk/some concerns	Low risk	Some concerns	Some concerns
[63]	Some concerns	Low risk	Low risk/Some concerns	Low risk for EEG and driving incidents; some concerns for subjective sleepiness	Some concerns	Some concerns
[64]	Some concerns	Low risk	Some concerns	Low risk	Some concerns	Some concerns
[65]	Some concerns	Some concerns	High risk	Some concerns	Some concerns	High risk
[66]	Some concerns	Low risk/some concerns	Low risk	Low risk	High risk	High risk
[67]	Some concerns	Low risk	Low risk/Some concerns	Low risk	Some concerns	Some concerns
[68]	Some concerns	Low risk	Some concerns	Low risk/Some concerns	Some concerns	Some concerns
[69]	Some concerns	Low risk/some concerns	Low risk	Low risk/some concerns	Some concerns	Some concerns
[70]	Some concerns	Low risk/some concerns	Low risk	Low risk	Some concerns	Some concerns
[78]	Some concerns	Some concerns	Some concerns	Some concerns	Some concerns	Some concerns
[72]	Some concerns	Some concerns	Low risk/some concerns	Low risk for objective outcomes; some concerns for KSS	Some concerns	Some concerns
[73]	Some concerns	Low risk	Some concerns	Low risk/Some concerns	Some concerns	Some concerns
[74]	Some concerns	Low risk	Low risk	Low risk	Some concerns	Some concerns
[75]	Some concerns	Low risk	Low risk for the caffeine EEG outcomes	Low risk	Some concerns	Some concerns
[76]	Some concerns	Low risk	Low risk (waking EEG)	Low risk	High risk	High risk
[77]	Some concerns	Low risk	Some concerns	Low risk	Some concerns	Some concerns

**Table 3:** Risk of bias of included studies (ROBINS-I).

Study	Bias Due to Confounding	Bias in Selection of Participants	Bias in Classification of Interventions	Bias Due to Deviations from Intended Interventions	Bias Due to Missing Data	Bias in Measurement of Outcomes	Bias in Selection of Reported Result	Overall Judgment
[32]	Serious	Moderate	Low to moderate	Serious	Moderate	Moderate	Serious	Serious
[39]	Serious	Moderate	Low to moderate	Moderate to serious	Moderate/unclear	Moderate	Serious	Serious
[40]	Serious	Moderate	Low to moderate	Serious	Low to moderate	Moderate	Serious	Serious
[45]	Serious	Moderate	Low to moderate	Moderate	Low	Moderate	Serious	Serious
[46]	Critical	Serious	Moderate	Moderate to serious	Low to moderate	Moderate	Serious	Critical
[60]	Serious	Moderate	Low to moderate	Serious	Moderate	Moderate to serious	Serious	Serious
[71]	Serious	Moderate to serious	Low	Moderate	Moderate	Moderate	Serious	Serious

## Data Availability

No new data were created or analyzed in this study. Data sharing is not applicable to this article.

## Supplementary Materials

The following supporting information can be downloaded at: https://www.mdpi.com/article/10.3390/nu18172898/s1.
