# Supplementary material for: The Caffeinated Brain Part 3: The Effect of Caffeine on Electroencephalography (EEG) During Wakefulness—A Systematic and Mechanistic Review
Source: Nutrients. 2026 Sep 3;18(17):2898. doi: 10.3390/nu18172898 (PMC13567186; doi:10.3390/nu18172898)
Supplement: Supplementary file 1 [file nutrients-18-02898-s001.zip › Supplementary table S1 nutrients-4458782.pdf]

Database / platform	Search strategy	Controlled vocabulary / field tags	Limits / filters	Search date	Records retrieved
PubMed/MEDLINE — NCBI PubMed	`((caffeine[Title/Abstract] OR coffee[Title/Abstract] OR caffeinated[Title/Abstract] OR methylxanthine[Title/Abstract]) AND (electroencephalography[Title/Abstract] OR EEG[Title/Abstract] OR qEEG[Title/Abstract] OR "quantitative EEG"[Title/Abstract] OR "event-related oscillations"[Title/Abstract] OR "spectral power"[Title/Abstract] OR "functional connectivity"[Title/Abstract] OR microstate[Title/Abstract] OR "brain oscillations"[Title/Abstract]) AND (wakefulness[Title/Abstract] OR awake[Title/Abstract] OR vigilance[Title/Abstract] OR alertness[Title/Abstract] OR "resting state"[Title/Abstract] OR "eyes open"[Title/Abstract] OR "eyes closed"[Title/Abstract] OR attention[Title/Abstract] OR cognition[Title/Abstract]))`	`[Title/Abstract]`; PubMed supports explicit field tags in square brackets.	Database inception–30 May 2026; English-language and peer-reviewed eligibility applied during selection unless an English filter was actually used at search stage	30 May 2026	150
Scopus — Elsevier	`TITLE-ABS-KEY((caffeine OR coffee OR caffeinated OR methylxanthine) AND (electroencephalography OR EEG OR qEEG OR "quantitative EEG" OR "event-related`	`TITLE-ABS-KEY` searches title, abstract and keywords in Scopus	Database inception–30 May 2026; English-language and peer-reviewed eligibility applied	30 May 2026	185

Database / platform	Search strategy	Controlled vocabulary / field tags	Limits / filters	Search date	Records retrieved
	oscillations" OR "spectral power" OR "functional connectivity" OR microstate OR "brain oscillations") AND (wakefulness OR awake OR vigilance OR alertness OR "resting state" OR "eyes open" OR "eyes closed" OR attention OR cognition))		during selection unless these filters were actually activated in Scopus		
Web of Science Core Collection — Clarivate	TS=((caffeine OR coffee OR caffeinated OR methylxanthine) AND (electroencephalography OR EEG OR qEEG OR "quantitative EEG" OR "event-related oscillations" OR "spectral power" OR "functional connectivity" OR microstate OR "brain oscillations") AND (wakefulness OR awake OR vigilance OR alertness OR "resting state" OR "eyes open" OR "eyes closed" OR attention OR cognition))	TS= = Topic search (title, abstract, author keywords and Keywords Plus in WoS Core Collection)	Database inception–30 May 2026; document-language/publication-type restrictions should be reported only if actually applied	30 May 2026	155
Embase — [confirm: Elsevier Embase.com or Ovid]	If Embase.com was used: ('caffeine'/exp OR caffeine:ti,ab,kw OR coffee:ti,ab,kw OR caffeinated:ti,ab,kw OR methylxanthine:ti,ab,kw) AND ('electroencephalography'/exp OR electroencephalography:ti,ab,kw OR eeg:ti,ab,kw OR qeeg:ti,ab,kw OR 'quantitative eeg':ti,ab,kw OR 'event-related oscillations':ti,ab,kw OR 'spectral power':ti,ab,kw OR 'functional connectivity':ti,ab,kw OR microstate:ti,ab,kw OR 'brain oscillations':ti,ab,kw)	Emtree explosion /exp; title :ti; abstract :ab; keyword :kw. Embase supports Emtree terms, Boolean operators and field labels.	Database inception–30 May 2026; human/English filters should be reported here only if actually applied	30 May 2026	120

Database / platform	Search strategy	Controlled vocabulary / field tags	Limits / filters	Search date	Records retrieved
	`AND ('wakefulness'/exp OR wakefulness:ti,ab,kw OR awake:ti,ab,kw OR vigilance:ti,ab,kw OR alertness:ti,ab,kw OR 'resting state':ti,ab,kw OR 'eyes open':ti,ab,kw OR 'eyes closed':ti,ab,kw OR attention:ti,ab,kw OR cognition:ti,ab,kw)`				
APA PsycINFO — [confirm actual platform: EBSCOhost, Ovid, ProQuest, or APA PsycNet]	Core strategy: `(caffeine OR coffee OR caffeinated OR methylxanthine) AND (electroencephalography OR EEG OR qEEG OR "quantitative EEG" OR "event-related oscillations" OR "spectral power" OR "functional connectivity" OR microstate OR "brain oscillations") AND (wakefulness OR awake OR vigilance OR alertness OR "resting state" OR "eyes open" OR "eyes closed" OR attention OR cognition);` apply the platform-specific title/abstract/subject-heading syntax corresponding to the platform actually used	APA PsycINFO is available through several platforms, including APA PsycNet, EBSCO, Ovid and ProQuest, each with different command syntax.	Database inception–30 May 2026; English-language eligibility; report actual database filters if used	30 May 2026	70
Cochrane Library — Wiley; CENTRAL/search manager	`([mh Caffeine] OR caffeine:ti,ab,kw OR coffee:ti,ab,kw OR caffeinated:ti,ab,kw OR methylxanthine:ti,ab,kw) AND ([mh Electroencephalography] OR electroencephalography:ti,ab,kw OR EEG:ti,ab,kw OR qEEG:ti,ab,kw OR "quantitative EEG":ti,ab,kw OR "event-related oscillations":ti,ab,kw OR "spectral power":ti,ab,kw OR "functional connectivity":ti,ab,kw OR`	`[mh ...]` = MeSH descriptor; `:ti,ab,kw` = title, abstract and keyword fields. Cochrane's field guide documents combined `:ti,ab,kw` searching.	Database inception–30 May 2026; no additional limits unless actually used	30 May 2026	50

Database / platform	Search strategy	Controlled vocabulary / field tags	Limits / filters	Search date	Records retrieved
	`microstate:ti,ab,kw OR "brain oscillations":ti,ab,kw) AND ([mh Wakefulness] OR wakefulness:ti,ab,kw OR awake:ti,ab,kw OR vigilance:ti,ab,kw OR alertness:ti,ab,kw OR "resting state":ti,ab,kw OR "eyes open":ti,ab,kw OR "eyes closed":ti,ab,kw OR attention:ti,ab,kw OR cognition:ti,ab,kw)`				
Manual reference-list searching	Reference lists of relevant reviews and all eligible articles were examined for additional potentially relevant reports.	Not applicable	Same eligibility criteria as electronic searches	Up to 30 May 2026	0
Total identified before deduplication	—	—	—	—	730
