# Supplementary material for: The Caffeinated Brain Part 3: The Effect of Caffeine on Electroencephalography (EEG) During Wakefulness—A Systematic and Mechanistic Review
Source: Nutrients. 2026 Sep 3;18(17):2898. doi: 10.3390/nu18172898 (PMC13567186; doi:10.3390/nu18172898)
Supplement: Supplementary file 1 [file nutrients-18-02898-s001.zip › Supplementary table S2 nutrients-4458782.pdf]

Supplementary Table S2. Reports excluded following full-text assessment and primary reasons for exclusion.

Number of study	Study	Reason for exclusion
1.	Thölke P, Arcand-Lavigne M, Lajnef T, Frenette S, Carrier J, Jerbi K. Caffeine induces age-dependent increases in brain complexity and criticality during sleep. Commun Biol. 2025 Apr 30;8(1):685. doi: 10.1038/s42003-025-08090-z. PMID: 40307472; PMCID: PMC12044076.	Sleep
2.	Pauchon B, Beauchamps V, Gomez-Mérino D, Erblang M, Drogou C, Beers PV, Guillard M, Quiquempoix M, Léger D, Chennaoui M, Sauvet F. Caffeine Intake Alters Recovery Sleep after Sleep Deprivation. Nutrients. 2024 Oct 11;16(20):3442. doi: 10.3390/nu16203442. PMID: 39458438; PMCID: PMC11510014.	Sleep
3.	Baur DM, Dornbierer DA, Landolt HP. Concentration-effect relationships of plasma caffeine on EEG delta power and cardiac autonomic activity during human sleep. J Sleep Res. 2024 Oct;33(5):e14140. doi: 10.1111/jsr.14140. Epub 2024 Jan 14. PMID: 38221756.	Sleep
4.	Landolt HP, Dijk DJ, Gaus SE, Borbély AA. Caffeine reduces low-frequency delta activity in the human sleep EEG. Neuropsychopharmacology. 1995 May;12(3):229-38. doi: 10.1016/0893-133X(94)00079-F. PMID: 7612156.	Sleep

5.	Landolt HP, Werth E, Borbély AA, Dijk DJ. Caffeine intake (200 mg) in the morning affects human sleep and EEG power spectra at night. Brain Res. 1995 Mar 27;675(1-2):67-74. doi: 10.1016/0006-8993(95)00040-w. PMID: 7796154.	Sleep
6.	Landolt, H.P.; Dijk, D.J.; Gaus, S.E.; Borbely, A.A. CAFFEINE REDUCES LOW-FREQUENCY DELTA-ACTIVITY IN THE HUMAN SLEEP EEG. NEUROPSYCHOPHARMACOLOGY, Vol.12(3), pp.229-238.	Sleep
7.	Carrier J, Paquet J, Fernandez-Bolanos M, Girouard L, Roy J, Selmaoui B, Filipini D. Effects of caffeine on daytime recovery sleep: A double challenge to the sleep-wake cycle in aging. Sleep Med. 2009 Oct;10(9):1016-24. doi: 10.1016/j.sleep.2009.01.001. Epub 2009 Apr 1. PMID: 19342294.	Sleep
8.	Carrier J, Fernandez-Bolanos M, Robillard R, Dumont M, Paquet J, Selmaoui B, Filipini D. Effects of caffeine are more marked on daytime recovery sleep than on nocturnal sleep. Neuropsychopharmacology. 2007 Apr;32(4):964-72. doi: 10.1038/sj.npp.1301198. Epub 2006 Aug 23. PMID: 16936703.	Sleep
9.	35) Chen X, Zhang L, Yang D, Li C, An G, Wang J, Shao Y, Fan R, Ma Q. Effects of Caffeine on Event-Related Potentials and Neuropsychological Indices After Sleep Deprivation. Front Behav Neurosci. 2020 Jun 22;14:108. doi: 10.3389/fnbeh.2020.00108.	ERP

	PMID: 32714162; PMCID: PMC7347038.	
10.	Pan J, Takeshita T, Morimoto K. Acute caffeine effect on repeatedly measured P300. Environ Health Prev Med. 2000 Apr;5(1):13-7. doi: 10.1007/BF02935910. PMID: 21432205; PMCID: PMC2723445.	ERP
11.	Kawamura N, Maeda H, Nakamura J, Morita K, Nakazawa Y. Effects of caffeine on event-related potentials: comparison of oddball with single-tone paradigms. Psychiatry Clin Neurosci. 1996 Aug;50(4):217-21. doi: 10.1111/j.1440-1819.1996.tb02745.x. PMID: 9201779.	ERP
12.	Bailey K, Amlung MT, Morris DH, Price MH, Von Gunten C, McCarthy DM, Bartholow BD. Separate and joint effects of alcohol and caffeine on conflict monitoring and adaptation. Psychopharmacology (Berl). 2016 Apr;233(7):1245-55. doi: 10.1007/s00213-016-4208-y. Epub 2016 Jan 27. PMID: 26815360; PMCID: PMC4803583.	ERP
13.	39) Ruijter J, De Ruiter MB, Snel J. The effects of caffeine on visual selective attention to color: an ERP study. Psychophysiology. 2000 Jul;37(4):427-39. PMID: 10934901.	ERP
14.	40) Deslandes AC, Veiga H, Cagy M, Piedade R, Pompeu F, Ribeiro P. Effects of caffeine on visual evoked potential (P300) and neuromotor performance. Arq Neuropsiquiatr. 2004 Jun;62(2B):385-90. doi: 10.1590/s0004-282x2004000300002. Epub 2004 Jul 20. PMID: 15273831.	ERP

15.	Barry RJ, Johnstone SJ, Clarke AR, Rushby JA, Brown CR, McKenzie DN. Caffeine effects on ERPs and performance in an auditory Go/NoGo task. Clin Neurophysiol. 2007 Dec;118(12):2692-9. doi: 10.1016/j.clinph.2007.08.023. Epub 2007 Oct 1. PMID: 17905651.	ERP
16.	Březinová V. Effect of caffeine on sleep: EEG study in late middle age people. Br J Clin Pharmacol. 1974 Jun;1(3):203-8. doi: 10.1111/j.1365-2125.1974.tb00237.x. PMID: 22454948; PMCID: PMC1402564.	Sleep
17.	Paterson LM, Nutt DJ, Ivarsson M, Hutson PH, Wilson SJ. Effects on sleep stages and microarchitecture of caffeine and its combination with zolpidem or trazodone in healthy volunteers. J Psychopharmacol. 2009 Jul;23(5):487-94. doi: 10.1177/0269881109104852. Epub 2009 Apr 7.	Combination with other substances
18.	Tieges Z, Richard Ridderinkhof K, Snel J, Kok A. Caffeine strengthens action monitoring: evidence from the error-related negativity. Brain Res Cogn Brain Res. 2004 Sep;21(1):87-93. doi: 10.1016/j.cogbrainres.2004.06.001. PMID: 15325416.	ERP
19.	Lorist MM, Snel J. Caffeine effects on perceptual and motor processes. Electroencephalogr Clin Neurophysiol. 1997 May;102(5):401-13. doi: 10.1016/s0921-884x(97)95729-5. PMID: 9191584.	ERP
20.	51) Lorist, M.M., Snel, J.; Kok, A. Influence of caffeine on information processing stages in well rested and fatigued	ERP

	subjects. *Psychopharmacology* **113**, 411–421 (1994).	
21.	Saville CWN, de Morree HM, Dundon NM, Marcora SM, Klein C. Effects of caffeine on reaction time are mediated by attentional rather than motor processes. Psychopharmacology (Berl). 2018 Mar;235(3):749-759. doi: 10.1007/s00213-017-4790-7. Epub 2017 Dec 23. PMID: 29273820; PMCID: PMC5847000.	ERP
22.	Dixit A, Vaney N, Tandon OP. Evaluation of cognitive brain functions in caffeine users: a P3 evoked potential study. Indian J Physiol Pharmacol. 2006 Apr-Jun;50(2):175-80. PMID: 17051738.	ERP
23.	Diukova A, Ware J, Smith JE, Evans CJ, Murphy K, Rogers PJ, Wise RG. Separating neural and vascular effects of caffeine using simultaneous EEG-FMRI: differential effects of caffeine on cognitive and sensorimotor brain responses. Neuroimage. 2012 Aug 1;62(1):239-49. doi: 10.1016/j.neuroimage.2012.04.041. Epub 2012 Apr 28. PMID: 22561357; PMCID: PMC3778750.	ERP
24.	Jochumsen, M.; Lavesen, E.R.; Griem, A.B.; Falkenberg-Andersen, C.; Jensen, S.K.G. The Effect of Caffeine on Movement-Related Cortical Potential Morphology and Detection. *Sensors* **2024**, *24*, 4030.	ERP
25.	Barthel, T., Mechau, D., Wehr, T. *et al.* Readiness potential in different states of physical activation and after ingestion of taurine and/or caffeine containing drinks. *Amino Acids* **20**, 63–73 (2001).	ERP

26.	Lesar M, Sajovic J, Novaković D, Primožič M, Vetrih E, Sajovic M, Žnidaršič A, Rogelj P, Daffertshofer A, Levnajić Z, Drevenšek G. The complexity of caffeine's effects on regular coffee consumers. Heliyon. 2025 Jan 9;11(2):e41471. doi: 10.1016/j.heliyon.2024.e41471. PMID: 39897922; PMCID: PMC11786655.	ERP
27.	Francis AM, Anderson TJ, McKearney KJ, Schryver B, Tibbo PG, Fisher DJ. Understanding caffeine's impact on sustained attention in early phase psychosis: Unveiling cognitive insights through the use of EEG. Psychiatry Res Neuroimaging. 2025 Aug;351:112006. doi: 10.1016/j.pscychresns.2025.112006. Epub 2025 Jun 21.	ERP
28.	Lunsford-Avery JR, Kollins SH, Kansagra S, Wang KW, Engelhard MM. Impact of daily caffeine intake and timing on electroencephalogram-measured sleep in adolescents. J Clin Sleep Med. 2022 Mar 1;18(3):877-884. doi: 10.5664/jcsm.9736. PMID: 34710040; PMCID: PMC8883093.	Sleep
29.	63) Lorist MM, Snel J, Kok A, Mulder G. Influence of caffeine on selective attention in well-rested and fatigued subjects. Psychophysiology. 1994 Nov;31(6):525-34. doi: 10.1111/j.1469-8986.1994.tb02345.x. PMID: 7846213. sprawdzono, ERP	ERP
30.	64) Undy, J.W. Acute effects of caffeine on behavioural and ERP indices of attention in healthy, low consumers of caffeine	ERP
31.	Rao A, Hu H, Nobre AC. The effects of combined caffeine	ERP

	and glucose drinks on attention in the human brain. Nutr Neurosci. 2005 Jun;8(3):141-53. doi: 10.1080/10284150500096994. PMID: 16117181	
32.	Kraetsch HG, Hummel T, Lötsch J, Kussat R, Kobal G. Analgesic effects of propyphenazone in comparison to its combination with caffeine. Eur J Clin Pharmacol. 1996;49(5):377-82. doi: 10.1007/BF00203781. PMID: 8866632.	Combination with other substances
33.	Blanco-Díaz CF, Guerrero-Méndez CD, Bastos-Filho T, Jaramillo-Isaza S, Ruiz-Olaya AF. Effects of the concentration level, eye fatigue and coffee consumption on the performance of a BCI system based on visual ERP-P300. J Neurosci Methods. 2022 Dec 1;382:109722. doi: 10.1016/j.jneumeth.2022.109722. Epub 2022 Oct 5. PMID: 36208730.	ERP
34.	Bruce SE, Werner KB, Preston BF, Baker LM. Improvements in concentration, working memory and sustained attention following consumption of a natural citicoline-caffeine beverage. Int J Food Sci Nutr. 2014 Dec;65(8):1003-7. doi: 10.3109/09637486.2014.940286. Epub 2014 Jul 21. PMID: 25046515; PMCID: PMC4517431.	ERP
35.	Cropley, V., Croft, R., Silber, B. *et al.* Does coffee enriched with chlorogenic acids improve mood and cognition after acute administration in healthy elderly? A pilot study. *Psychopharmacology* **219**, 737–749 (2012).	ERP
36.	Drapeau C, Hamel-Hébert I, Robillard R, Selmaoui B,	Sleep

	Filipini D, Carrier J. Challenging sleep in aging: the effects of 200 mg of caffeine during the evening in young and middle-aged moderate caffeine consumers. J Sleep Res. 2006 Jun;15(2):133-41. doi: 10.1111/j.1365-2869.2006.00518.x. PMID: 16704567.	
37.	Robillard R, Bouchard M, Cartier A, Nicolau L, Carrier J. Sleep is more sensitive to high doses of caffeine in the middle years of life. J Psychopharmacol. 2015 Jun;29(6):688-97. doi: 10.1177/0269881115575535. Epub 2015 Mar 10. PMID: 25759402.	Sleep
38.	Beaumont M, Batéjat D, Coste O, Doireau P, Chauffard F, Enslen M, Lagarde D, Pierard C. Recovery after prolonged sleep deprivation: residual effects of slow-release caffeine on recovery sleep, sleepiness and cognitive functions. Neuropsychobiology. 2005;51(1):16-27. doi: 10.1159/000082851. PMID: 15627809.	Sleep
39.	79) de Morree HM, Klein C, Marcora SM. Cortical substrates of the effects of caffeine and time-on-task on perception of effort. J Appl Physiol (1985). 2014 Dec 15;117(12):1514-23. doi: 10.1152/japplphysiol.00898.2013. Epub 2014 Oct 23. PMID: 25342703.	ERP
40.	Conners CK. The acute effects of caffeine on evoked response, vigilance, and activity level in hyperkinetic children. J Abnorm Child Psychol. 1979 Jun;7(2):145-51. doi: 10.1007/BF00918895. PMID: 469108.	ERP

41.	Ruijter J, Lorist MM, Snel J, De Ruiter MB. The influence of caffeine on sustained attention: an ERP study. Pharmacol Biochem Behav. 2000 May;66(1):29-37. doi: 10.1016/s0091-3057(00)00229-x. PMID: 10837841.	ERP
42.	Ruijter J, de Ruiter MB, Snel J, Lorist MM. The influence of caffeine on spatial-selective attention: an event-related potential study. Clin Neurophysiol. 2000 Dec;111(12):2223-33. doi: 10.1016/s1388-2457(00)00462-4. PMID: 11090776.	ERP
43.	Barry RJ, De Blasio FM, Fogarty JS. Caffeine affects children's ERPs and performance in an equiprobable go/no-go task: Testing a processing schema. Psychophysiology. 2019 May;56(5):e13330. doi: 10.1111/psyp.13330. Epub 2019 Jan 18. PMID: 30657186.	ERP
44.	Trunk A, Stefanics G, Zentai N, Bacskay I, Felinger A, Thuróczy G, Hernádi I. Lack of interaction between concurrent caffeine and mobile phone exposure on visual target detection: an ERP study. Pharmacol Biochem Behav. 2014 Sep;124:412-20. doi: 10.1016/j.pbb.2014.07.011. Epub 2014 Jul 27. PMID: 25073015.	ERP
45.	Barry RJ, De Blasio FM, Cave AE. Caffeine Effects on ERP Components and Performance in an Equiprobable Auditory Go/NoGo Task. J Caffeine Res. 2014 Sep 1;4(3):83-92. doi: 10.1089/jcr.2014.0011. PMID: 25229010; PMCID: PMC4158968.	ERP
46.	Montenegro M, Veiga H, Deslandes A, Cagy M,	Combination with other substances

	McDowell K, Pompeu F, Piedade R, Ribeiro P. Neuromodulatory effects of caffeine and bromazepam on visual event-related potential (P300): a comparative study. Arq Neuropsiquiatr. 2005 Jun;63(2B):410-5. doi: 10.1590/s0004-282x2005000300009. Epub 2005 Jul 25. PMID: 16059590.	
47.	Maciejewska K, Grabowska K. Acute effect of energy boost dietary supplement on P3 waveform: double blind, placebo controlled study. Acta Neurobiol Exp (Wars). 2020;80(4):411-423. PMID: 33350994.	ERP
48.	Reeves RR, Struve FA, Patrick G. The effects of caffeine withdrawal on cognitive P300 auditory and visual evoked potentials. Clin Electroencephalogr. 1999 Jan;30(1):24-7. doi: 10.1177/155005949903000110. PMID: 9891189.	ERP
49.	Seidl R, Peyrl A, Nicham R, Hauser E. A taurine and caffeine-containing drink stimulates cognitive performance and well-being. Amino Acids. 2000;19(3-4):635-42. doi: 10.1007/s007260070013. PMID: 11140366.	ERP
50.	Lorist MM, Snel J, Mulder G, Kok A. Aging, caffeine, and information processing: an event-related potential analysis. Electroencephalogr Clin Neurophysiol. 1995 Sep;96(5):453-67. doi: 10.1016/0168-5597(95)00069-5. PMID: 7555918.	ERP
51.	Ho SC, Chung JW. The effects of caffeine abstinence on sleep: a pilot study. Appl Nurs Res. 2013 May;26(2):80-4. doi:	Sleep

	10.1016/j.apnr.2012.08.004. Epub 2012 Dec 4. PMID: 23218455.	
52.	LaJambe CM, Kamimori GH, Belenky G, Balkin TJ. Caffeine effects on recovery sleep following 27 h total sleep deprivation. Aviat Space Environ Med. 2005 Feb;76(2):108-13. PMID: 15742825.	Sleep
53.	Reichert CF, Veitz S, Bühler M, Gruber G, Deuring G, Rehm SS, Rentsch K, Garbazza C, Meyer M, Slawik H, Lin YS, Weibel J. Wide awake at bedtime? Effects of caffeine on sleep and circadian timing in male adolescents - A randomized crossover trial. Biochem Pharmacol. 2021 Sep;191:114283. doi: 10.1016/j.bcp.2020.114283. Epub 2020 Oct 15. PMID: 33069664.	Sleep
54.	Karacan I, Thornby JI, Anch M, Booth GH, Williams RL, Salis PJ. Dose-related sleep disturbances induced by coffee and caffeine. Clin Pharmacol Ther. 1976 Dec;20(6):682-9. doi: 10.1002/cpt1976206682. PMID: 186223.	Sleep
55.	Pajcin M, White JM, Banks S, Dorrian J, Paech GM, Grant CL, Johnson K, Tooley K, Aidman E, Fidock J, Kamimori GH, Della Vedova CB. Effects of strategic early-morning caffeine gum administration on association between salivary alpha-amylase and neurobehavioural performance during 50 h of sleep deprivation. Accid Anal Prev. 2019 May;126:160-172. doi: 10.1016/j.aap.2018.01.026. Epub 2018 May 7. PMID: 29402402.	Sleep

56.	Weibel J, Lin YS, Landolt HP, Garbazza C, Kolodyazhniy V, Kistler J, Rehm S, Rentsch K, Borgwardt S, Cajochen C, Reichert CF. Caffeine-dependent changes of sleep-wake regulation: Evidence for adaptation after repeated intake. Prog Neuropsychopharmacol Biol Psychiatry. 2020 Apr 20;99:109851. doi: 10.1016/j.pnpbp.2019.109851. Epub 2019 Dec 19. PMID: 31866308.	Sleep
57.	Bonnet MH, Arand DL. Metabolic rate and the restorative function of sleep. Physiol Behav. 1996 Apr-May;59(4-5):777-82. doi: 10.1016/0031-9384(95)02093-4. PMID: 8778866.	Sleep
58.	Wesensten NJ, Killgore WD, Balkin TJ. Performance and alertness effects of caffeine, dextroamphetamine, and modafinil during sleep deprivation. J Sleep Res. 2005 Sep;14(3):255-66. doi: 10.1111/j.1365-2869.2005.00468.x. PMID: 16120100.	Sleep
59.	Dongen, V., Hans, P. A., Price, N. J., Mullington, J. M., Szuba, M. P., Kapoor, S. C., & Dinges, D. F. (2001). Caffeine eliminates psychomotor vigilance deficits from sleep inertia. *Sleep*, 24(7), 813–819.	Sleep
60.	110) Rétey, J. V., Adam, M., Khatami, R., Luhmann, U. F. O., Jung, H. H., Berger, W., & Landolt, H. P. (2007). A genetic variation in the adenosine A2A receptor gene (ADORA2A) contributes to individual sensitivity to caffeine effects on sleep. *Clinical Pharmacology & Therapeutics*, 81(5), 692–698.	Sleep

61.	Youngberg MR, Karpov IO, Begley A, Pollock BG, Buysse DJ. Clinical and physiological correlates of caffeine and caffeine metabolites in primary insomnia. J Clin Sleep Med. 2011 Apr 15;7(2):196-203. PMID: 21509336; PMCID: PMC3077349.	Sleep
62.	Lorist MM, Snel J, Kok A, Mulder G. Acute effects of caffeine on selective attention and visual search processes. Psychophysiology. 1996 Jul;33(4):354-61. doi: 10.1111/j.1469-8986.1996.tb01059.x. PMID: 8753934.	ERP
63.	Wicht CA, De Pretto M, Mouthon M, Spierer L. Neural correlates of expectations-induced effects of caffeine intake on executive functions. Cortex. 2022 May;150:61-84. doi: 10.1016/j.cortex.2022.02.004. Epub 2022 Mar 11. PMID: 35366604.	ERP
64.	Ghisolfi ES, Schuch A, Strimitzer IM Jr, Luersen G, Martins FF, Ramos FL, Becker J, Lara DR. Caffeine modulates P50 auditory sensory gating in healthy subjects. Eur Neuropsychopharmacol. 2006 Apr;16(3):204-10. doi: 10.1016/j.euroneuro.2005.09.001. Epub 2005 Nov 8. PMID: 16278075.	ERP
65.	Tharion WJ, Kobrick JL, Lieberman HR, Fine BJ. Effects of caffeine and diphenhydramine on auditory evoked cortical potentials. Percept Mot Skills. 1993 Jun;76(3 Pt 1):707-15. doi: 10.2466/pms.1993.76.3.707. PMID: 8321577.	ERP
66.	Wolpaw JR, Penry JK. Effects of ethanol, caffeine, and	ERP

	placebo on the auditory evoked response. Electroencephalogr Clin Neurophysiol. 1978 May;44(5):568-74. doi: 10.1016/0013-4694(78)90124-4. PMID: 77763.	
67.	Kahathuduwa CN, Dassanayake TL, Amarakoon AMT, Weerasinghe VS. Acute effects of theanine, caffeine and theanine-caffeine combination on attention. Nutr Neurosci. 2017 Jul;20(6):369-377. doi: 10.1080/1028415X.2016.1144845. Epub 2016 Feb 12. PMID: 26869148.	ERP
68.	Nawarathna GS, Ariyasinghe DI, Dassanayake TL. High-dose L-theanine-caffeine combination improves neurobehavioural and neurophysiological measures of selective attention in acutely sleep-deprived young adults: a double-blind, placebo-controlled crossover study. Br J Nutr. 2025 Aug 14;134(3):195-204. doi: 10.1017/S0007114525104169. Epub 2025 Aug 12. PMID: 40789769; PMCID: PMC12491391.	ERP
69.	De Pauw K, Roelands B, Knaepen K, Polfliet M, Stiens J, Meeusen R. Effects of caffeine and maltodextrin mouth rinsing on P300, brain imaging, and cognitive performance. J Appl Physiol (1985). 2015 Mar 15;118(6):776-82. doi: 10.1152/japplphysiol.01050.2014. Epub 2015 Jan 22. PMID: 25614603.	ERP
70.	Werre PF, Mattie H, Berretty EW. Contingent negative variation, extraversion, reaction time and drug effects. *Personality and Individual*	ERP

	Differences. 2001;30:1083–1094.	
71.	Ruijter, J., Lorist, M. M., & Snel, J. (1999). The influence of different doses of caffeine on visual task performance. *Journal of Psychophysiology, 13*(1), 37–48.	ERP
72.	Werre PF, Mattie H, Fortgens C, Berretty EW, Sluiter W. Interaction between extraversion and drug-induced conditions as indicated by the contingent negative variation. Biol Psychol. 1994 Dec;39(1):45-56. doi: 10.1016/0301-0511(94)90056-6. PMID: 7880947.	ERP
73.	Münte TF, Heinze HJ, Künkel H, Scholz M. Personality traits influence the effects of diazepam and caffeine on CNV magnitude. Neuropsychobiology. 1984;12(1):60-7. doi: 10.1159/000118111. PMID: 6514179.	ERP
74.	van den Berg B, de Jong M, Woldorff MG, Lorist MM. Caffeine Boosts Preparatory Attention for Reward-related Stimulus Information. J Cogn Neurosci. 2021 Jan;33(1):104-118. doi: 10.1162/jocn_a_01630. Epub 2020 Sep 28. PMID: 32985946.	ERP
75.	Maciejewska KJ. Decoding event-related potentials: single-dose energy dietary supplement acts on earlier brain processes than we thought. Front Neuroinform. 2025 Jul 8;19:1563893. doi: 10.3389/fninf.2025.1563893. PMID: 40697956; PMCID: PMC12279804.	ERP
76.	Tieges Z, Snel J, Kok A, Plat N, Ridderinkhof R. Effects of caffeine on anticipatory control	ERP

	processes: evidence from a cued task-switch paradigm. Psychophysiology. 2007 Jul;44(4):561-78. doi: 10.1111/j.1469-8986.2007.00534.x. Epub 2007 May 31. PMID: 17539921.	
77.	Tieges Z, Snel J, Kok A, Wijnen JG, Lorist MM, Richard Ridderinkhof K. Caffeine improves anticipatory processes in task switching. Biol Psychol. 2006 Aug;73(2):101-13. doi: 10.1016/j.biopsycho.2005.12.005. Epub 2006 Mar 23. PMID: 16549227.	ERP
78.	Kuiper, M. The effect of habitual caffeine consumption on inhibition	ERP
79.	130) Neill MG, Fletcher EKS, Larson E, Fraser K, Ramsay S, Smirl JD, Emery CA. Neurophysiology of Downhill Mountain Bike Athletes-Benchmark Assessments of Event-Related Potentials. Sensors (Basel). 2025 Sep 1;25(17):5388. doi: 10.3390/s25175388. PMID: 40942821; PMCID: PMC12431017.	ERP
80.	Srivastava N, Sarkar D. Effect of caffeine on information processing using P3 evoked potential study. Int J Sci Res. 2024;13(2):36–38.	ERP
81.	Hirvonen J, Jääskeläinen IP, Näätänen R, Sillanaukee P. Adenosine A1/A2a receptors mediate suppression of mismatch negativity by ethanol in humans. Neurosci Lett. 2000 Jan 7;278(1-2):57-60. doi: 10.1016/s0304-3940(99)00897-6. PMID: 10643800.	ERP
82.	Martin FH, Garfield J. Combined effects of alcohol and caffeine on the late components of the event-related	ERP

	potential and on reaction time. Biol Psychol. 2006 Jan;71(1):63-73. doi: 10.1016/j.biopsycho.2005.01.004. PMID: 16360882.	
83.	Murd C, Aru J, Hiio M, Luiga I, Bachmann T. Caffeine enhances frontal relative negativity of slow brain potentials in a task-free experimental setup. Brain Res Bull. 2010 Apr 29;82(1-2):39-45. doi: 10.1016/j.brainresbull.2010.01.013. Epub 2010 Feb 1. PMID: 20123003.	ERP
84.	De Pauw K, Roelands B, Van Cutsem J, Marusic U, Torbeyns T, Meeusen R. Electro-physiological changes in the brain induced by caffeine or glucose nasal spray. Psychopharmacology (Berl). 2017 Jan;234(1):53-62. doi: 10.1007/s00213-016-4435-2. Epub 2016 Sep 23. PMID: 27664111.	ERP
85.	Bonnet MH, Arand DL. The use of prophylactic naps and caffeine to maintain performance during a continuous operation. Ergonomics. 1994 Jun;37(6):1009-20. doi: 10.1080/00140139408963714. PMID: 8026448.	Sleep
86.	Weibel J, Lin YS, Landolt HP, Kistler J, Rehm S, Rentsch KM, Slawik H, Borgwardt S, Cajochen C, Reichert CF. The impact of daily caffeine intake on nighttime sleep in young adult men. Sci Rep. 2021 Feb 25;11(1):4668. doi: 10.1038/s41598-021-84088-x. PMID: 33633278; PMCID: PMC7907384.	Sleep
87.	Weibel J, Lin YS, Landolt HP, Berthomier C, Brandewinder M, Kistler J, Rehm S, Rentsch KM, Meyer M, Borgwardt S,	Sleep

	Cajochen C, Reichert CF. Regular Caffeine Intake Delays REM Sleep Promotion and Attenuates Sleep Quality in Healthy Men. J Biol Rhythms. 2021 Aug;36(4):384-394. doi: 10.1177/07487304211013995. Epub 2021 May 23. PMID: 34024173; PMCID: PMC8276335.	
88.	Drake C, Roehrs T, Shambroom J, Roth T. Caffeine effects on sleep taken 0, 3, or 6 hours before going to bed. J Clin Sleep Med. 2013 Nov 15;9(11):1195-200. doi: 10.5664/jcsm.3170. PMID: 24235903; PMCID: PMC3805807.	Sleep
